# The therapeutic effectiveness of ^177^Lu-lilotomab in B-cell non-Hodgkin lymphoma involves modulation of G2/M cell cycle arrest

**DOI:** 10.1038/s41375-019-0677-4

**Published:** 2019-12-13

**Authors:** Alexandre Pichard, Sara Marcatili, Jihad Karam, Julie Constanzo, Riad Ladjohounlou, Alan Courteau, Marta Jarlier, Nathalie Bonnefoy, Sebastian Patzke, Vilde Stenberg, Peter Coopman, Guillaume Cartron, Isabelle Navarro-Teulon, Ada Repetto-Llamazares, Helen Heyerdahl, Jostein Dahle, Manuel Bardiès, Jean-Pierre Pouget

**Affiliations:** 1 Institut de Recherche en Cancérologie de Montpellier (IRCM), Inserm U1194, Université de Montpellier, Institut Régional du Cancer de Montpellier (ICM), Montpellier, F-34298 France; 2 UMR 1037 INSERM/UPS, Centre de Recherche en Cancérologie de Toulouse, Toulouse, F-31062 France; 3 Institut Régional du Cancer de Montpellier (ICM), Montpellier F-34298, France, Montpellier, France; 4 Nordic Nanovector ASA, Kjelsåsveien 168 B, 0884 Oslo, Norway; 5 Department of Radiation Biology, Institute for Cancer Research, OUH-Norwegian Radium Hospital, Oslo, Norway; 6 Département d’Hématologie, UMR–CNRS 5235, CHU de Montpellier, Montpellier, France

**Keywords:** Radiotherapy, Cancer immunotherapy, B-cell lymphoma

## Abstract

Some patients with B-cell non-Hodkin lymphoma Lymphoma (NHL) become refractory to rituximab (anti-CD20 antibody) therapy associated with chemotherapy. Here, the effect of the anti-CD37 antibody-radionuclide conjugate lutetium-177 (^177^Lu)-lilotomab (Betalutin^®^) was investigated in preclinical models of NHL. In SCID mice bearing DOHH2 (transformed follicular lymphoma, FL) cell xenografts, ^177^Lu-lilotomab significantly delayed tumor growth, even at low activity (100 MBq/kg). In athymic mice bearing OCI-Ly8 (diffuse large B-cell lymphoma, DLBCL) or Ramos (Burkitt’s lymphoma) cell xenografts, ^177^Lu-lilotomab activity had to be increased to 500 MBq/kg to show a significant tumor growth delay. Clonogenic and proliferation assays showed that DOHH2 cells were highly sensitive to ^177^Lu-lilotomab, while Ramos cells were the least sensitive, and U2932 (DLBCL), OCI-Ly8, and Rec-1 (mantle cell lymphoma) cells displayed intermediate sensitivity. The strong ^177^Lu-lilotomab cytotoxicity observed in DOHH2 cells correlated with reduced G2/M cell cycle arrest, lower WEE-1- and MYT-1-mediated phosphorylation of cyclin-dependent kinase-1 (CDK1), and higher apoptosis. In agreement, ^177^Lu-lilotomab efficacy in vitro, in vivo, and in patient samples was increased when combined with G2/M cell cycle arrest inhibitors (MK-1775 and PD-166285). These results indicate that ^177^Lu-lilotomab is particularly efficient in treating tumors with reduced inhibitory CDK1 phosphorylation, such as transformed FL.

## Introduction

B-cell non-Hodgkin lymphoma (NHL) originates from B lymphocytes at various stages of differentiation, from precursor to mature cells. Currently, most patients with B-cell NHL are treated with anti-CD20 monoclonal antibodies (mAb) (e.g., rituximab) and chemotherapy [1, 2]. The response rate to rituximab alone is rather modest [3], and after treatment, some lymphomas become refractory to this therapy [4–7]. The 5-year overall survival rate is reduced in patients with follicular lymphoma (FL) who experience disease progression or relapse within 2 years after first-line immuno-chemotherapy compared with those without relapse [8, 9]. Similar results were observed in diffuse large B-cell lymphoma (DLBCL) with dramatic outcome in patients who are refractory to immuno-chemotherapy [10]. Moreover, heavily pretreated, elderly and frail patients with FL often have comorbidities that limit their ability to tolerate chemotherapy and other myelosuppressive therapies [11]. Therefore, new treatments are needed for patients who are refractory to immuno-chemotherapy.

**Fig. 1:**
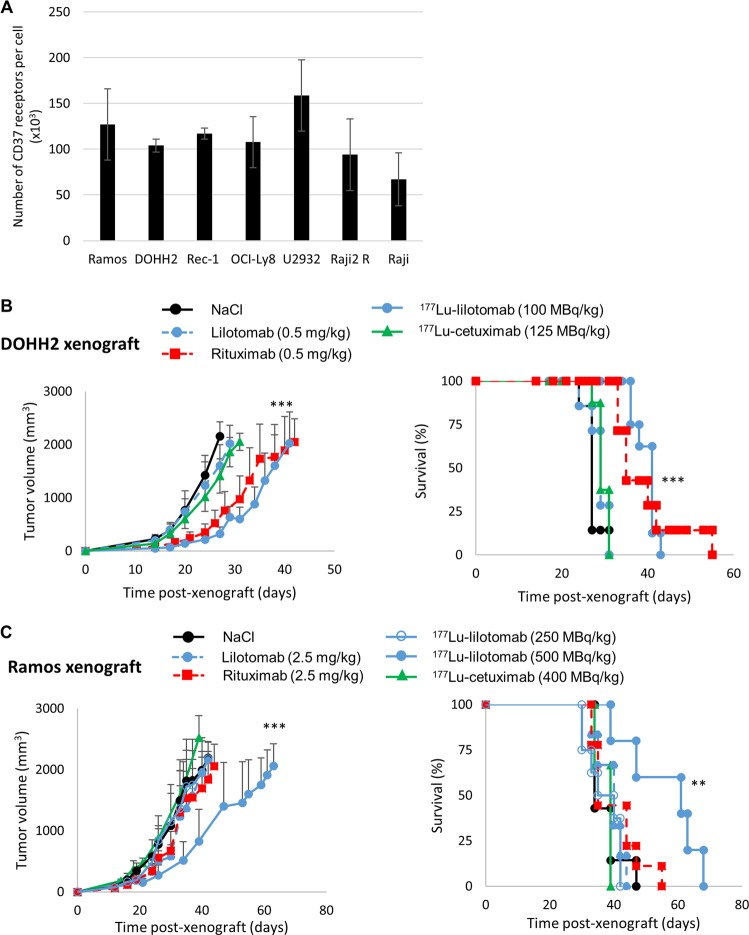
In vivo therapeutic efficacy of unlabeled antibodies and of ^177^Lu-lilotomab. **a** The number of CD37 receptors per cell was determined in all the cell lines by Scatchard analysis (*n* = 3) [26]. **b** SCID mice bearing DOHH2 cell xenografts received one intravenous injection of ^177^Lu-lilotomab (100 MBq/kg, 0.5 mg/kg), nonspecific ^177^Lu-cetuximab (125 MBq/kg, 0.6 mg/kg), or unlabeled mAbs (0.5 mg/kg) (*n* = 6–8/group). Tumor growth (left panel) was plotted as a function of time post xenograft, and Kaplan–Meyer survival curves were established (right panel). **c** Athymic mice bearing Ramos cell xenografts received one intravenous injection of ^177^Lu-lilotomab at 250 MBq/kg or 500 MBq/kg, ^177^Lu-cetuximab at 400 MBq/kg, or unlabeled mAbs (2.5 mg/kg) (*n* = 6–9/group). Tumor growth (left panel) was monitored as a function of time post xenograft, and Kaplan–Meyer survival curves were established (right panel); *p ≤ 0.05, **p ≤ 0.01, ***p ≤ 0.001 (compared with the NaCl-treated group).

Radioimmunotherapy (RIT), in which radiolabeled antibodies are used to combine radiation and antibody cytotoxic properties [12], shows significant efficacy in NHL [13, 14]. Two anti-CD20 mAbs, ibritumomab tiuxetan radiolabeled with yttrium-90 (Zevalin^®^, Spectrum Pharmaceuticals, USA) and tositumomab radiolabeled with iodine-131 (Bexxar^®^, GlaxoSmithKline, UK), were approved for NHL treatment by FDA in 2002 and 2003, respectively. However, Zevalin^®^ and Bexxar^®^ are used after several rounds of treatment with rituximab, and the remaining circulating rituximab may impair the efficacy of anti-CD20 RIT [15]. Therefore, a conjugate that targets a different antigen could be desirable. Lutetium-177 [^177^Lu]-lilotomab satetraxetan (Betalutin^®^, previously known as ^177^Lu-DOTA-HH1) is a next generation radioimmunoconjugate in which the murine mAb lilotomab targets CD37 receptors expressed on mature and malignant B cells [16, 17], but also, at lower levels, in T cells, macrophages/monocytes, granulocytes, and dendritic cells [18]. ^177^Lu is a beta-emitter with a mean beta energy of 0.133 MeV (mean and max beta-range in water: 0.23 and 1.9 mm). CD37 (tetraspanin TSPAN26) is a 31 kDa transmembrane protein that belongs, to the tetraspanin family, and CD20 is a member of the MS4A family [19]. Both proteins are involved in cell membrane organization and co-signaling [18, 20, 21]. CD37 has a bivalent role in the phosphatidylinositol 3′-kinase (PI3K)/AKT survival pathway in tumor suppression and in humoral immunity [22]. As CD37 is highly expressed in NHL cells (Fig. 1a), it represents an attractive molecule for targeted therapy [23–29]. The loss of CD37 expression predicts significantly lower survival rates in patients with DLBCL treated with rituximab and R-CHOP, particularly in those with germinal center B-cell like DLBCL [30]. ^177^Lu-lilotomab is currently tested in a clinical phase 1 study for the treatment of relapsed/refractory DLBCL (https://clinicaltrials.gov; NCT02658968), and in a phase 2b trial (PARADIGME) for the treatment of third-line CD20 immunotherapy-refractory FL (https://clinicaltrials.gov; NCT01796171) [31] with promising preliminary results. A first clinical report indicates that Betalutin^®^ is well tolerated and highly active in recurrent indolent NHL, especially in FL [32].

The aim of this study was to determine the molecular mechanisms involved in the therapeutic response to ^177^Lu-lilotomab in order to identify (i) NHL sub-types that could benefit most from this treatment, and (ii) relevant therapeutic associations.

## Materials and methods

### Cell lines and cell surface receptor quantification

The Ramos and Raji (Burkitt’s lymphoma, BL), the rituximab-resistant Raji cells (Raji2R), DOHH2 (transformed FL), U2932 and OCI-Ly8 (DLBCL), and Rec-1 (mantle cell lymphoma) cell lines were used in this study. The cell origins and cell culture methods are detailed in Supplementary Methodology. The Scatchard method was used for determining the level of CD20 and CD37 receptors.

### Antibody radiolabeling

The anti-CD37 lilotomab (Nordic Nanovector, Oslo, Norway) and cetuximab (anti-EGFR antibody) (Erbitux™, Lilly, Indianapolis, In, USA) conjugated with p-SCN-benzyl-DOTA (Macrocyclics, Plano, Tx, USA) were labeled with ^177^Lu (^177^Lu-mAbs) at the specific activity of 200 MBq/mg, as previously described [26, 27].

### Animals and tumor xenografts

Athymic nude-Foxn1 mice (athymic mice hereafter) were xenografted with Ramos or OCI-Ly8 cells, while C.B-17/lcrHan™Hsd-Prkdc^scid^ mice (SCID mice hereafter) were xenografted with DOHH2 cells (see Supplementary Methodology). Athymic and SCID mice were next intravenously injected (at 7 and 13 days post xenograft, respectively) with ^177^Lu-lilotomab, rituximab, NaCl, unlabeled lilotomab or the nonspecific ^177^Lu-cetuximab (Supplementary Methodology). Tumor growth was evaluated and the biodistribution of radiolabeled mAbs was determined as described in Supplementary Methodology.

### Clonogenic survival and proliferation assays

The clonogenic cell survival assay was used to assess ^177^Lu-lilotomab and unlabeled mAbs cytotoxicity in vitro in Ramos and DOHH2 cells (see Supplementary Methodology). Cell proliferation was also assessed in cells at 126 h post treatment (see Supplementary Methodology). Clonogenic survival and proliferation rates were calculated as the percentage of the value in nontreated cells (NT) set to 100%.

### Apoptosis, micronucleus assay, and cell cycle progression measurement

Apoptosis, micronuclei, and cell cycle progression were measured in cells exposed to rituximab, lilotomab, or ^177^Lu-lilotomab. The effect of WEE-1 and MYT-1 kinase inhibitors on the cell cycle was also assessed (see Supplementary Methodology).

### ATP and HMGB1 release

The release of the high mobility group box 1 (HMGB1) protein and ATP by treated cells was also quantified (see Supplementary Methodology).

### WEE-1 and MYT-1 inhibitors and western blotting

Protein expression was assessed in cells exposed to ^177^Lu-lilotomab in the presence or not of the selective WEE-1 kinase inhibitor MK-1775 (Selleckchem, Houston, USA) or of the dual WEE-1/MYT-1 inhibitor PD-166285 (EMD Merck Millipore/Calbiochem, Molsheim, France) (Supplementary Methodology).

### Treatment of patients’ tumor cell samples with ^177^Lu-lilotomab alone or in combination with MK-1775 or PD-166285

Samples from patients (*n* = 4) with DLBCL or FL obtained from the Centre for Biological Resources (CBR)/Hemodiag facility of Montpellier University Hospital (Jérôme Moreaux, responsable de la collection « Hématologie 8 » du Centre de Ressources Biologiques du CHU de Montpellier - http://www.chu-montpellier.fr (Identifiant BIOBANQUES - BB-0033-00031) were grown and incubated with ^177^Lu-lilotomab (0–6 MBq/mL) alone or with MK-1775 or PD-166285 (1 µM) for 18 h (Supplementary Methodology). The quantity and proportion of living tumor (CD45^+^/CD3^−^/CD19^+^/CD20^+^/kappa^+^) and nontumor cells were analyzed and the Bliss independence model was used to analyze the efficacy of the ^177^Lu-lilotomab + MK-1775/PD-166285 combination (Supplementary Methodology) [33].

### Statistical analysis

Detailed statistical analysis is described in Supplementary Methodology.

## Results

### ^177^Lu-lilotomab is more efficient in DOHH2 than Ramos tumor cell xenografts

Scatchard analysis confirmed CD37 expression in the tumor cell lines used for this study. No statistical difference (*p* = 0.35) was observed between Ramos and DOHH2 cells (Fig. 1a). Assessment of the therapeutic efficacy (tumor growth and survival) of ^177^Lu-lilotomab in SCID mice bearing DOHH2 cell xenografts showed that the median survival (MS) of mice treated with 100 MBq/kg ^177^Lu-lilotomab increased to 41 days (*p* < 0.001) compared with NaCl-treated animals (MS = 27 days) (Fig. 1b). The MS was also increased after treatment with rituximab, but to a lower extent (35 days; *p* < 0.001). Conversely, the MS was not significantly different in mice treated with 0.5 mg/kg lilotomab (29 days), with NaCl (*p* = 0.2252) or with ^177^Lu-cetuximab (nontargeting antibody) (29 days; *p* = 0.3363).

In athymic mice bearing Ramos cell xenografts, MS was not different in animals treated with 250 MBq/kg ^177^Lu-lilotomab (MS = 35 days), NaCl (MS = 34 days), 2.5 mg/kg rituximab (35 days), or lilotomab (40 days) (*p* = 0.9935, *p* = 0.5529, and *p* = 0.8119, respectively) (Fig. 1c). Conversely, the injection of 500 MBq/kg ^177^Lu-lilotomab increased the MS (61 days) compared with NaCl (*p* = 0.0091).

The MS was not significantly different in mice treated with ^177^Lu-cetuximab or NaCl (39 versus 34 days; *p* = 0.4773). No body weight loss was observed after treatment (Supplementary Fig. 1a).

### Similar tumor uptake of ^177^Lu-rituximab and ^177^Lu-lilotomab

The analysis of the biodistribution at various time points and SPECT-CT imaging at 48 h after injection of ^177^Lu-lilotomab and ^177^Lu-rituximab in Ramos and DOHH2 cell xenografts (Supplementary Fig. 1b, c) showed that radioactivity uptake was higher in Ramos than in DOHH2 cell tumors for both ^177^Lu-mAbs. This was mainly due to the larger size (100–200 mm^3^ versus 50–75 mm^3^) of the Ramos tumors (Supplementary Fig. 1b, c) at the time of injection. Conversely, the uptake of the two ^177^Lu-mAbs was similar in each model, although the number of CD20 receptors was higher than that of CD37 receptors in both DOHH2 and Ramos cells (Supplementary Fig. 1d). This could be explained by higher CD37 receptor internalization compared with CD20.

### Clonogenic assays confirm that ^177^Lu-lilotomab is more efficient in DOHH2 than in Ramos cells

**Fig. 2:**
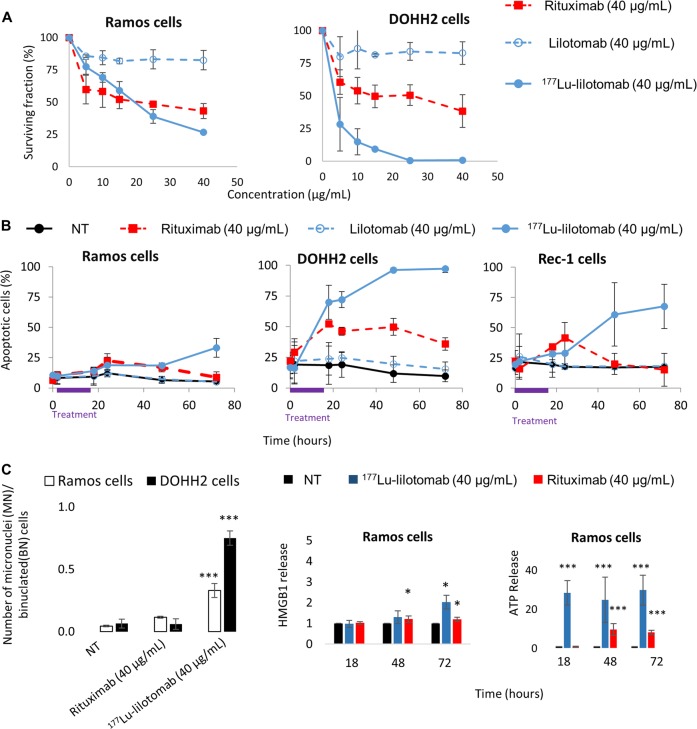
Clonogenic cell survival, apoptosis, micronucleus formation, and release of DAMPs. **a** Clonogenic survival was assessed in Ramos and DOHH2 cells at day 12 after exposure to rituximab or lilotomab (0–40 µg/mL), or ^177^Lu-lilotomab (0–6 MBq/mL; 0–40 µg/mL) for 18 h. **b** Apoptosis in Ramos, DOHH2 and Rec-1 cells was measured using the Annexin V-FITC/7-AAD Kit and flow cytometry at different time points during and after exposure to 40 µg/mL of lilotomab or rituximab, or 6 MBq/mL of ^177^Lu-lilotomab for 18 h and in nontreated cells (NT). **c** Micronucleus formation was measured in Ramos and DOHH2 cells at day 3 after exposure to 40 µg/mL rituximab or to 6 MBq/mL ^177^Lu-lilotomab (left panel). Immunogenic cell death was assessed by quantifying ATP and HMGB1 release from Ramos cells exposed to^177^Lu-lilotomab or rituximab (right panel). Results are the mean ± SD of three experiments performed in triplicate.

Incubation with 0–40 µg/mL rituximab or lilotomab reduced the clonogenic survival of DOHH2 and Ramos cells compared with NT (Fig. 2a). Cytotoxicity in both cell lines was further increased by using ^177^Lu-lilotomab at the same mAb concentration (0–6 MBq/mL; 0–40 µg/mL) (Fig. 2a). The strongest cytotoxic effect was observed in DOHH2 cells. As Rec-1 cells do not form colonies, a proliferation assay was used to test their sensitivity to ^177^Lu-lilotomab with intermediate results between Ramos and DOHH2 cells (Supplementary Fig. 2a). Proliferation was similarly reduced by ^177^Lu-lilotomab in rituximab-resistant (Raji2R) and parental Raji cells (Supplementary Fig. 2b).

### ^177^Lu-lilotomab strongly induces apoptosis in DOHH2 cells

The analysis of apoptosis induction by incubation with ^177^Lu-lilotomab or unlabeled antibodies showed that compared with NT, rituximab, but not lilotomab, induced apoptosis in the three cell lines with a peak at 18–24 h (Fig. 2b), particularly in DOHH2 cells, followed by Rec-1 cells, and finally Ramos cells. The percentage of apoptotic cells was further increased in DOHH2 and Rec-1 cells and to a lower extent in Ramos cells upon exposure to ^177^Lu-lilotomab (Fig. 2b).

### ^177^Lu-lilotomab strongly induces mitotic death

To obtain some insights into the mechanisms involved in clonogenic death of Ramos cells exposed to ^177^Lu-lilotomab, mitotic death was assessed using the micronucleus assay (Fig. 2c, left panel). Compared with NT, micronucleus formation was significantly higher in Ramos and DOHH2 cells exposed to ^177^Lu-lilotomab (*p* < 0.0001 for both), but not to rituximab (*p* = 0.0815 and *p* = 0.922, respectively).

### ^177^Lu-lilotomab induces release of ATP and of HMGB1

As immunogenic cell death might contribute to the in vivo ^177^Lu-lilotomab therapeutic efficacy, the release of HMGB1 and ATP, which act as damage-associated molecular patterns (DAMPs) that may stimulate the immune response, was investigated. ATP release was significantly increased particularly in Ramos cells incubated with ^177^Lu-lilotomab and to a lower extent, with rituximab (Fig. 2c, right panel). HMGB1 release was more modest and was mainly observed in DOHH2 cells exposed to ^177^Lu-lilotomab (Supplementary Fig. 2c). Moreover, preliminary experiments suggested that calreticulin surface expression was strongly induced at 48 h after incubation of Ramos and DOHH2 cells with ^177^Lu-lilotomab or rituximab (less important in DOHH2 cells) (data not shown).

These results indicate that Ramos and DOHH2 cells exposed to ^177^Lu-lilotomab (and to a lower extent to rituximab) release DAMPs (ATP, HMGB1, and calreticulin) that might contribute to the host immune antitumor response.

### ^177^Lu-lilotomab induces G2/M arrest in Ramos cells, but not in DOHH2 cells

**Fig. 3:**
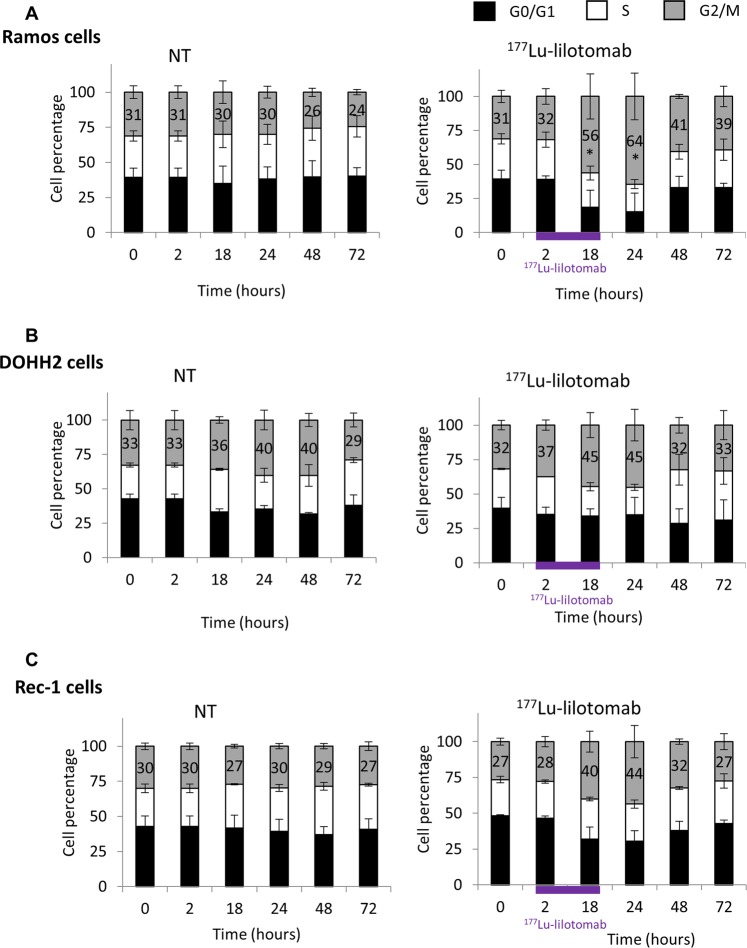
Cell cycle analysis. The percentage of cells in the G0/G1, S and G2/M phases (mean ± SD of three experiments in triplicate) was determined by flow cytometry in **a** Ramos, **b** DOHH2, and **c** Rec-1 cells at different time points during and after exposure to 0 and 6 MBq/mL of ^177^Lu-lilotomab for 18 h.

Cell cycle analysis showed that the percentage of Ramos cells in the G2/M phase was strongly increased between 18 h and 48 h after exposure to ^177^Lu-lilotomab compared with NT (×2.1 at 24 h; *p* = 0.0495) (Fig. 3a and Supplementary Fig. 3). This effect was not observed in ^177^Lu-lilotomab-treated DOHH2 cells (×1.1 versus NT at 24 h, *p* = 0.827) (Fig. 3b and Supplementary Fig. 3), and was intermediate in ^177^Lu-lilotomab-treated Rec-1 cells (×1.5 versus NT at 24 h, *p* = 0.1266) (Fig. 3c and Supplementary Fig. 3). A transient increase in the percentage of Ramos and DOHH2 cells in G1 at 18 h was observed upon incubation with rituximab (×1.5 and ×1.6 versus NT, respectively), but not with lilotomab (×1.1 for both cell lines versus NT) (Supplementary Fig. 4a, b). The two antibodies did not have any effect in Rec-1 cells (Supplementary Fig. 4c).

### ^177^Lu-lilotomab leads to decreased CDK1 expression and phosphorylation in DOHH2 cells

**Fig. 4:**
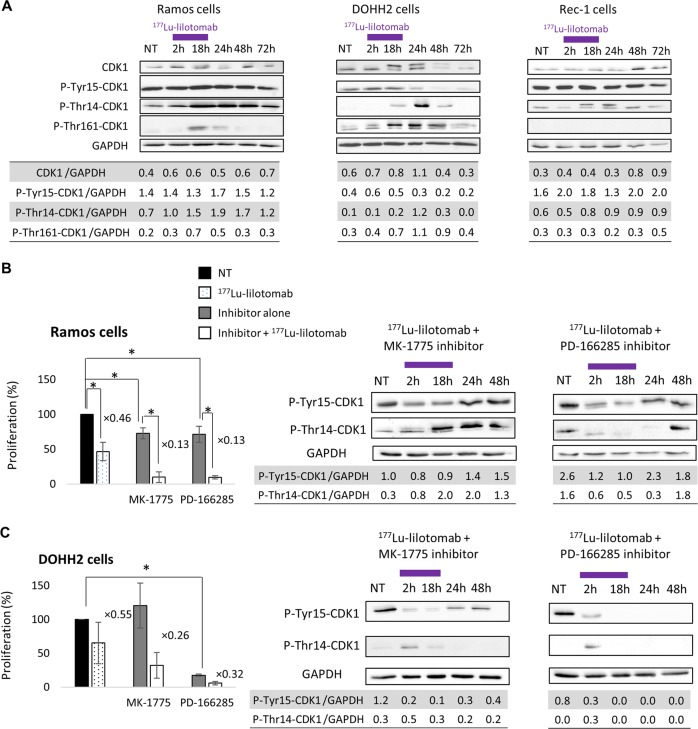
Protein expression and effect of G2/M arrest inhibitors on cell proliferation. **a** Expression of proteins involved in G2/M cell cycle arrest (CDK1, p-Thr161-CDK1, p-Tyr15-CDK1, p-Thr14-CDK1) was assessed by western blotting in Ramos, DOHH2 and Rec-1 cells during and after exposure to 0 (NT) and 6 MBq/mL of ^177^Lu-lilotomab for 18 h. Cell proliferation (flow cytometry) and CDK1 phosphorylation at Tyr15 and Thr14 (western blotting) were determined in **b** Ramos and **c** DOHH2 cells during and after exposure to 0 (NT) and 6 MBq/mL ^177^Lu-lilotomab alone, or with 1 µM MK-1775 (WEE-1 inhibitor) or 1 µM PD-166285 (WEE-1 and MYT-1 inhibitor). Proliferation was calculated as the percentage of the value in nontreated cells (left black bar) set to 100%. Data are the mean ± SD of three independent experiments in triplicate (*p < 0.05).

During the G2 phase of the cell cycle, CDK1, the master kinase that controls the G2/M transition, is activated by A- and B-type cyclins [34, 35]. CDK1 phosphorylation at Thr161 promotes G2/M cell cycle progression. Conversely, CDK1 phosphorylation on Tyr15 and Thr14 by WEE-1 and MYT-1, respectively [36, 37], blocks cells in G2/M [38]. Western blot analysis during/after incubation with ^177^Lu-lilotomab showed that the total CDK1 level remained stable in Ramos and Rec-1 cells (Fig. 4a), whereas in DOHH2 cells it progressively increased and then started to decrease from 48 h. Moreover, in Ramos and Rec-1 cells exposed to ^177^Lu-lilotomab, CDK1 phosphorylation at Tyr15 and Thr14 increased between 18 and 48 h, whereas phosphorylation at Thr161 was slightly and transiently induced (only in Ramos cells at 18–24 h; Fig. 4a). Conversely, in DOHH2 cells (Fig. 4a), after a slight and transient increase, CDK1 phosphorylation at Tyr15 strongly decreased at 24 and 48 h. Moreover phosphorylation at Thr14 and at Thr161 increased between 18 and 48 h. In OCI-Ly8 and U2932 cells (Supplementary Fig. 5a), CDK1 phosphorylation at Tyr15 increased between 18 and 48 h, and phosphorylation at Thr14 was slightly increased at 24 h.

Finally, CDK7 expression slightly increased in DOHH2, but not in Ramos or Rec-1 cells (Supplementary Fig. 5b). The expression of WEE-1, which mediates CDK1 phosphorylation at Tyr15 leading to G2/M arrest, decreased in Ramos, Rec-1 and DOHH2 cells exposed to ^177^Lu-lilotomab, and became undetectable in DOHH2 cells at 24 h (Supplementary Fig. 5b). These observations are in line with the accumulation of Ramos, but not of DOHH2 cells in the G2/M phase after exposure to ^177^Lu-lilotomab. Similarly, the expression of MYT-1, which is involved in CDK1 phosphorylation at Thr14, decreased in all three cell lines from 18 h (Supplementary Fig. 5b).

### Inhibition of the G2/M checkpoint sensitizes Ramos, Rec-1, OCI-Ly8, and U2932 cells to ^177^Lu-lilotomab

Ramos, Rec-1, OCI-Ly8, and U2932 cells were incubated with the WEE-1 inhibitor MK-1775 or the WEE-1/MYT-1 inhibitor PD-166285 to stop the induction of the G2/M checkpoint after the treatment with ^177^Lu-lilotomab.

MK-1775 (*p* = 0.0495) and PD-166285 (*p* = 0.0495) reduced Ramos cell proliferation compared with NT (Fig. 4b), and also enhanced the antiproliferative effect of ^177^Lu-lilotomab compared with ^177^Lu-lilotomab alone (*p* = 0.0495 for MK-1775; *p* = 0.0495 for PD-166285).

In DOHH2 cells, PD-166285 (*p* = 0.05) but not MK-1775 (*p* = 1) inhibited the cell proliferation compared with NT (Fig. 4c). ^177^Lu-lilotomab antiproliferative effect was increased by co-incubation with MK-1775 and PD-166285 compared with ^177^Lu-lilotomab alone, but this effect was not significant (*p* = 0.1213 and *p* = 0.1213, respectively).

However, in Ramos cells, CDK1 phosphorylation at Tyr15 only slightly decreased at 2 and 18 h upon co-incubation with MK-1775 and ^177^Lu-lilotomab (phosphorylation at Thr14 was unchanged). Conversely the decrease of phosphorylation at Tyr15 and Thr14 was more pronounced when PD-166285 was used (compare Fig. 4a, b). In DOHH2 cells, CDK1 phosphorylation at Tyr15 and Thr14 strongly decreased and remained low throughout (compare Fig. 4a, c).

**Fig. 5:**
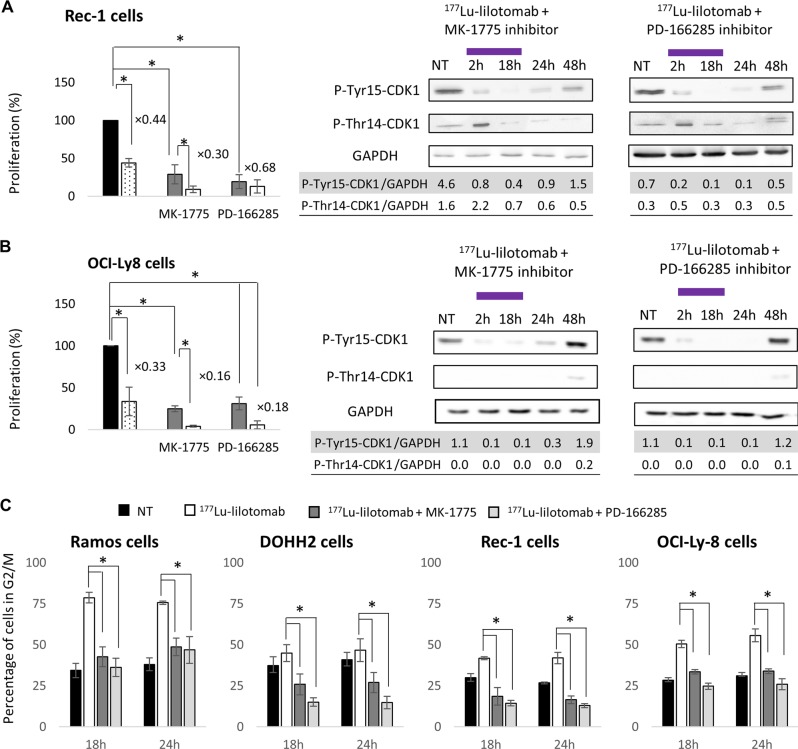
Effect of G2/M arrest inhibitors on cell proliferation and on cell cycle. Cell proliferation (flow cytometry) and CDK1 phosphorylation at Tyr15 and Thr14 (western blotting) were determined in **a** Rec-1, and **b** OCI-Ly8 cells during and after exposure to 0 (NT) and 6 MBq/mL ^177^Lu-lilotomab alone, or with 1 µM MK-1775 (WEE-1 inhibitor) or 1 µM PD-166285 (WEE-1 and MYT-1 inhibitor). Proliferation was calculated as the percentage of the value in nontreated cells (left black bar) set to 100%. Data are the mean ± SD of three independent experiments in triplicate (*p < 0.05). **c** The percentage of Ramos, DOHH2, Rec-1, and OCI-Ly8 cells in the G2/M cell cycle upon exposure or not (NT) to ^177^Lu-lilotomab, ^177^Lu-lilotomab + 1 µM MK-1775 (WEE-1 inhibitor), or PD-166285 (WEE-1 and MYT-1 inhibitor) was determined. Data are the mean ± SD of three independent experiments in triplicate.

A significant reduction in proliferation was also observed in Rec-1 cells after incubation with MK-1775 or PD-166285 (*p* = 0.0495 and *p* = 0.0495, respectively, compared with NT). The antiproliferative effect of ^177^Lu-lilotomab was increased by co-incubation with MK-1775 compared with ^177^Lu-lilotomab alone (*p* = 0.0495), but not with PD-166285 (*p* = 0.2752) (Fig. 5a). ^177^Lu-lilotomab antiproliferative effect in the presence of MK-1775 or PD-166285 was comparable in Ramos, Rec-1, and DOHH2 cells (*p* = 0.5127, *p* = 0.2752, and *p* = 0.1213, respectively).

Proliferation was reduced also in OCI-Ly8 (Fig. 5b) and U2932 cells (Supplementary Fig. 6a) after incubation with MK-1775 or PD-166285 compared with NT (*p* = 0.0495 and *p* = 0.0495, respectively, for OCI-Ly8 cells; *p* = 0.0495 and *p* = 0.0495, respectively, for U2932 cells). ^177^Lu-lilotomab antiproliferative effect also was increased by co-incubation with MK-1775 or with PD-166285 (*p* = 0.0495 versus ^177^Lu-lilotomab alone).

Moreover, co-incubation with MK-1775 or PD-166285 and ^177^Lu-lilotomab reduced the fraction of G2/M cells compared with ^177^Lu-lilotomab alone in all five cell lines (*p* = 0.0495; Fig. 5c and Supplementary Fig. 6b). Cell cycle analysis after treatment with inhibitors alone did not show any statistically significant difference compared with NT (Supplementary Fig. 6c).

### Inhibitors of G2/M checkpoint sensitizes Ramos and OCI-Ly8 tumor xenografts to ^177^Lu-lilotomab

**Fig. 6:**
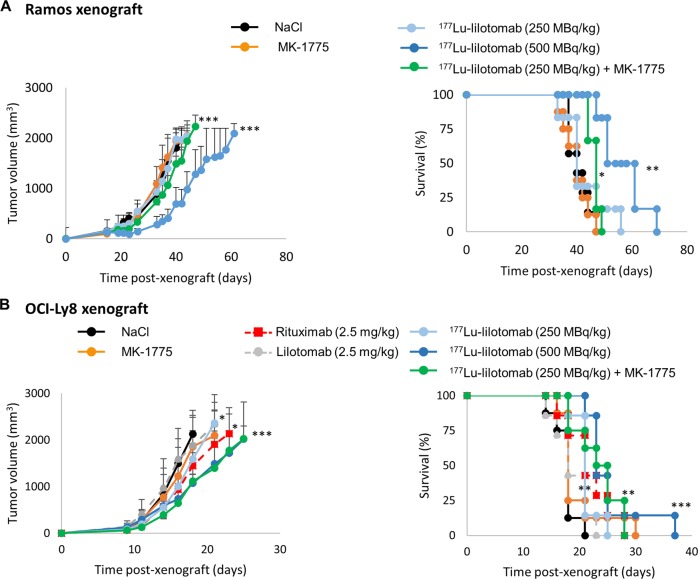
Effect of G2/M arrest inhibitors on tumor progression. **a** Athymic mice bearing Ramos cell xenografts received (i) one intravenous injection of ^177^Lu-lilotomab (250 MBq/kg or 500 MBq/kg), (ii) 30 mg/kg MK-1775 (twice a day) for 5 days; (iii) one injection of ^177^Lu-lilotomab (250 MBq/kg) + 30 mg/kg MK-1775 (twice a day) from day 1 to 5 post injection (*n* = 6–9/group). Tumor growth (left panel) was monitored as a function of time post xenograft, and Kaplan–Meyer survival curves were established (right panel). **b** Athymic mice bearing OCI-Ly8 cell xenografts received (i) one intravenous injection of ^177^Lu-lilotomab (250 MBq/kg or 500 MBq/kg), (ii) 30 mg/kg MK-1775 (twice a day) for 5 days, (iii) one injection of ^177^Lu-lilotomab (250 MBq/kg) + 30 mg/kg MK-1775 (twice a day) from day 1 to 5 post injection, (iv) unlabeled mAbs (2.5 mg/kg) (*n* = 6–8/group). Tumor growth (left panel) was monitored as a function of time post xenograft, and Kaplan–Meyer survival curves were established (right panel). *p ≤ 0.05, **p ≤ 0.01, ***p ≤ 0.001 (compared with the NT/NaCl-treated group).

In Ramos cell xenografts, the combination of 250 MBq/kg ^177^Lu-lilotomab and MK-1775 more strongly inhibited tumor growth (*p* = 0.001) and increased MS (from 40 to 47 days; *p* = 0.0156) compared with 250 MBq/kg ^177^Lu-lilotomab alone (Fig. 6a, Supplementary Fig. 7a).

In OCI-Ly8 cells xenografts (Fig. 6b, Supplementary Fig. 7b), neither lilotomab nor MK-1775 had any therapeutic efficacy (*p* = 0.475 and *p* = 0.625, respectively, compared with control). Conversely, ^177^Lu-lilotomab (250 MBq/kg) significantly inhibited tumor growth (*p* = 0.015) and increased MS (*p* = 0.0062). The ^177^Lu-lilotomab (250 MBq/kg) and MK-1775 combination reduced tumor growth more effectively than ^177^Lu-lilotomab alone (*p* = 0.05). The combination was as efficient as 500 MBq/kg ^177^Lu-lilotomab alone (*p* = 0.7070). No toxicity was associated with MK-1775 treatment in vivo (Supplementary Fig. 7c).

### ^177^Lu-lilotomab cytotoxicity in DLBCL and FL biopsies is improved by the association with G2/M checkpoint inhibitors

Flow cytometry analysis of cell surface markers (CD45, CD19, CD3, CD20, and kappa) (Supplementary Fig. 8a) in cells isolated from four patient biopsies after incubation or not with ^177^Lu-lilotomab alone or in combination with MK-1775 or PD-166285 for 18 h showed that the proportion of living malignant cells (CD3-negative and CD20 and kappa positive) was higher in nontreated FL (27.1–29.9%) than in nontreated DLBCL (0.36–0.52%) samples. It must be noted that for DLBCL biopsies, the number of living cells after ^177^Lu-lilotomab treatment was low (especially for DLBCL1) (Supplementary Table 1). At day 4, the proportion of tumor cells was reduced in all samples incubated with ^177^Lu-lilotomab (*p* = 0.044) (Supplementary Fig. 8b).

**Fig. 7:**
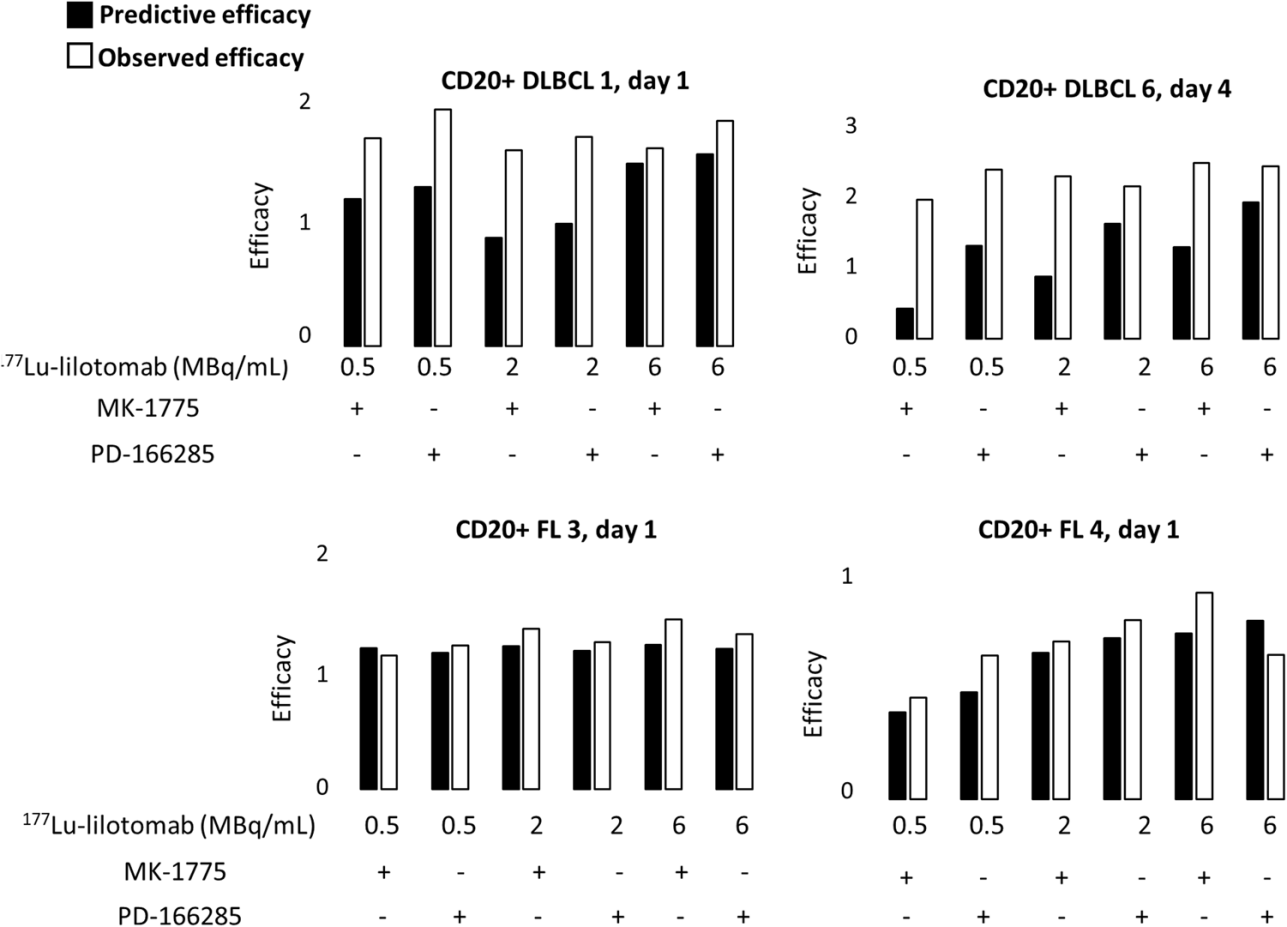
Theoretical and experimental efficacy of ^177^Lu-lilotomab alone or combined with G2/M arrest inhibitors in tumor samples from patients with NHL. Theoretical (using the Bliss independence mathematical model) and experimental antiproliferative effects of ^177^Lu-lilotomab and/or MK-1775 or PD-166285 (18 h incubation). The analysis was done at day 1 and day 4 post treatment.

Finally, the effect of the ^177^Lu-lilotomab and MK-1775 or PD-166285 combination was assessed using the Bliss independence model (see “Methods” section) at the end of exposure (day 1) and also 3 days later (day 4) (Fig. 7; Supplementary Fig. 9a). Comparison of the predicted and observed proliferation rates upon exposure to the drug combinations indicated that MK-1775 and PD-166285 increased ^177^Lu-lilotomab cytotoxicity through a synergistic mechanisms (*p* = 0.037).

## Discussion

**Fig. 8:**
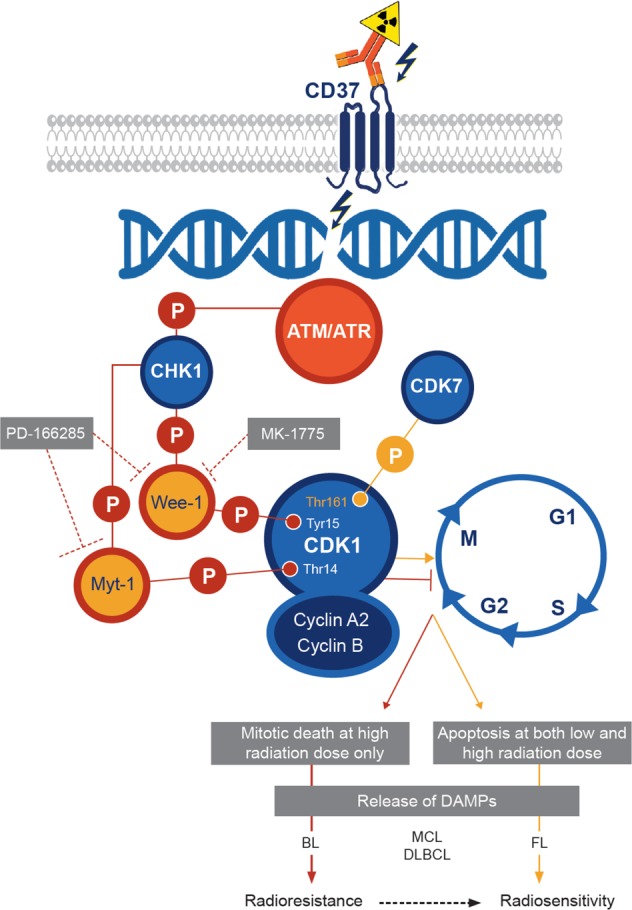
Proposed mechanism of ^177^Lu-lilotomab action. Beta particles emitted by ^177^Lu cause DNA double strand breaks that are recognized by the ATM/ATR sensor proteins. In Burkitt’s lymphoma (BL) and mantle cell lymphoma (MCL) cells, this leads to CHK1 phosphorylation and activation and consequently to phosphorylation of WEE-1 and MYT-1. This subsequently induces CDK1 phosphorylation at Tyr15 and Thr14, and G2/M cell cycle arrest to allow DNA repair. Conversely in follicular lymphoma (FL) cells, phosphorylation of CDK1 at Thr161 by CDK7 promotes G2/M cell cycle progression. Unrepaired DNA lesions lead to apoptosis.

In this study, we investigated the mechanisms involved in the therapeutic efficacy of the next generation radioimmunoconjugate ^177^Lu-lilotomab (Betalutin^®^) in preclinical B-cell NHL models and in patient biopsies (see model Fig. 8).

In SCID mice xenografted with the relatively radiosensitive DOHH2 cells, one injection of ^177^Lu-lilotomab at the MTA (100 MBq/kg, 0.5 mg/kg) significantly improved MS compared with NaCl or the corresponding amount of rituximab. Conversely, in mice xenografted with the more radioresistant Ramos cells, 250 MBq/kg ^177^Lu-lilotomab did not improve MS compared with NaCl or rituximab, although the radioactivity uptake was about 15 times higher in Ramos than in DOHH2 cell xenografts (Supplementary Fig. 1c). However, with 500 MBq/kg ^177^Lu-lilotomab (i.e., a tumor uptake 30 times higher than in DOHH2 cell tumors), MS was significantly improved and higher than with rituximab, which was ineffective in this model (even at 10 mg/kg, data not shown). Similarly, in OCI-Ly8 cell xenografts showing intermediate radioresistance between DOHH2 and Ramos cells, ^177^Lu-lilotomab efficacy was strongly enhanced when activity was increased to 500 MBq/kg (Fig. 6b). We would like to highlight that initially, all in vivo experiments (Ramos, OCI-Ly8, and DOHH2 cell xenografts) were planned in athymic nude mice. These experiments were possible for Ramos and OCI-Ly8 cells. Conversely, DOHH2 cell xenografts did not grow in athymic nude mice. Therefore, SCID mice were used because they display a lower immune response (lack of B cells) that facilitates the acceptance and growth of tumor xenografts. However, as SCID mice are also more radiation-sensitive, it was not possible to inject activities higher than 100–125 MBq/kg without observing toxic side effects.

We then confirmed in vitro the higher sensitivity of DOHH2 cells to ^177^Lu-lilotomab compared with Ramos cells, and investigated the underlying mechanisms using also Rec-1, OCI-Ly8, and U2932 cells that showed intermediate sensitivity to ^177^Lu-lilotomab compared with Ramos and DOHH2 cells. Our in vitro data indicate that resistance to ^177^Lu-lilotomab is mainly associated with arrest in the G2/M phase of the cell cycle upon exposure to this conjugate. This effect was strong in Ramos cells and progressively lower in U2932, Rec-1, and OCI-Ly8 cells, and not significant in DOHH2 cells. The lack of G2/M cell cycle arrest could explain the strong apoptosis induction and mitotic death observed with ^177^Lu-lilotomab.

Our results indicate that apoptosis induction alone cannot explain the therapeutic efficacy of high activity of ^177^Lu-lilotomab (500 MBq/kg) in Ramos tumor xenograft models, and that mitotic death (micronucleus assay) also is significantly involved in the cytotoxic effects of ^177^Lu-lilotomab, but not of rituximab. Hence, we hypothesized that mitotic death was responsible for ^177^Lu-lilotomab antitumor effect in vivo. Moreover, the observation that micronucleus formation is proportional to the radiation dose [39] could explain why ^177^Lu-lilotomab has an antitumor effect at 500 MBq/kg, but not at 250 MBq/kg.

We next investigated the level of CDK1, phosphorylated at Tyr15 and Thr14 (involved in G2/M cell cycle arrest) and of CDK1 phosphorylated at Thr161 (involved in cell cycle progression). CDK1 phosphorylation at Thr161 (located in the activation loop) is mediated by the CAK kinase, a trimetric protein complex consisting of CDK7, cyclin H, and MAT1. Conversely, CDK1 phosphorylation on Tyr15 and Thr14 by WEE-1 and MYT-1, respectively [36, 37], blocks cells in G2/M [38]. Cells can be released from this block by protein phosphatase-mediated dephosphorylation of these residues. These kinases are involved in the DNA-damage response through DNA repair pathways and cell cycle checkpoints that inhibit cell cycle progression during DNA repair [40–42]. In radiosensitive DOHH2 cells, CDK1 phosphorylation at Tyr15 and Thr14 decreased, whereas phosphorylation at Thr161 increased upon incubation with ^177^Lu-lilotomab. Conversely in Ramos, Rec-1, OCI-Ly8, and U2932 cells, CDK1 phosphorylation at Tyr15 and Thr14 remained high, whereas phosphorylation at Thr161 was low when measured in Ramos and Rec-1 cells. We confirmed in OCI-Ly8 and Ramos cell xenograft models (to a lower extent) as well as in four human NHL biopsies the role of CDK1 phosphorylation at Tyr15 and Thr14 in G2/M cell cycle arrest and in cell death by using WEE-1 and MYT-1 inhibitors. Specifically, WEE-1 inhibition and subsequently CDK1 phosphorylation at Tyr15 (by MK-1775) sensitized Ramos, Rec-1, OCI-Ly8, and U2932 cells to ^177^Lu-lilotomab. On the other hand, concomitant WEE-1 and MYT-1 inhibition and consequently CDK1 phosphorylation at Tyr15 and Thr14 (by PD-166285) did not have any additive effect on proliferation. A similar trend was observed in (CD45+, CD19+, CD3−, and CD20+) tumor cells isolated from human biopsies. This suggests that radiosensitivity (assessed by measuring cell proliferation) is mainly determined by WEE-1 activity. It must be noted that MK-1775 significantly reduced Ramos cell proliferation (Fig. 4b), but only slightly influenced CDK1 phosphorylation at Tyr15 compared with PD-166285. This might explain why MK-1775 was less efficient in delaying tumor growth in Ramos xenografts than in OCI-Ly8 xenografts (Fig. 6). Moreover, it indicates that modifications of protein expression/phosphorylation levels are not strictly accompanied by the same effects on tumor cell proliferation in vitro and in vivo.

For results obtained in patient samples, it must be kept in mind that radiation sensitivity is intimately linked to the proliferation index, which is lower in cells isolated from biopsies. Moreover, RIT with ^177^Lu-lilotomab would be proposed to patients who have become refractory to rituximab, and we have shown that ^177^Lu-lilotomab is similarly efficient in parental and in rituximab-resistant Raji cells (Raji2R). The combination of ^177^Lu-lilotomab with G2/M cell cycle arrest inhibitors could enhance the therapeutic efficacy, and may allow decreasing the injected amount of radioactivity.

These results are of a particular relevance because inhibitors of proteins required for cell cycle progression (e.g., CHK1/2, CDK4/CDK6, CDK1, and WEE-1) have gained interest for the treatment of solid and hematological tumors, and are currently assessed in clinical trials [43, 44]. Particularly, MK-1775 enhances the efficacy of SRC inhibitors in BL [45], and the combination of CHK1 and WEE-1 inhibitors is synergistic in mantle cell lymphoma [46]. Finally, in our in vivo experimental approach, the host immunological response was reduced because we used immunodeficient mice, although some ADCC effects could be expected because natural killer cells are active in both mouse strains. Nevertheless, our results indicate that Ramos and DOHH2 cells exposed to ^177^Lu-lilotomab generate DAMPs that can further stimulate the immune system. In a clinical setting, the immunological response could be enhanced by using the chimeric version of lilotomab that can activate ADCC [47].

## Conclusion

We showed that ^177^Lu-lilotomab is more efficient than rituximab in transformed FL preclinical models. ^177^Lu-lilotomab is also efficient in BL cells (more aggressive NHL sub-type), but much higher doses are required. Moreover, we found that reduced CDK1-mediated G2/M cell cycle arrest upon incubation with ^177^Lu-lilotomab is predictive of its efficacy. Release of Ramos, Rec-1, U2932, and OCI-ly8 cells from G2/M cell cycle arrest using a WEE-1 pharmacological inhibitor (MK-1775) sensitizes these cells to ^177^Lu-lilotomab. These results support preliminary data from clinical studies showing that ^177^Lu-lilotomab is particularly active in relapsed indolent lymphoma [32].

In conclusion, our study brings novel insights into ^177^Lu-lilotomab mechanisms of action in NHL treatment and its sensitization by drugs targeting CDK1 inhibitory kinases. These findings could be also used to improve patient selection, which is essential for increasing RIT efficacy and safety.

## Supplementary information

Supplementary Figure Legends

Supplementary Figure 1

Supplementary Figure 2

Supplementary Figure 3

Supplementary Figure 4

Supplementary Figure 5

Supplementary Figure 6

Supplementary Figure 7

Supplementary Figure 8

Supplementary Figure 9

Supplementary Table

Supplementary Methodology

Dataset 1

Dataset 2
