# Supplementary material for: The therapeutic effectiveness of 177Lu-lilotomab in B-cell non-Hodgkin lymphoma involves modulation of G2/M cell cycle arrest
Source: Leukemia. 2019 Dec 13;34(5):1315–28. doi: 10.1038/s41375-019-0677-4 (PMC7192854; doi:10.1038/s41375-019-0677-4)
Supplement: Supplementary file 1 — Supplementary Figure Legends [file 41375_2019_677_MOESM1_ESM.docx]

**Supplementary Figure Legends. Pichard *et al.***

**Supplementary Figure 1. Mice weight and ^177^Lu-lilotomab and ^177^Lu-rituximab biodistribution. (A)** Weekly monitoring of body weight of athymic and SCID mice bearing subcutaneous Ramos or DOHH2 cell tumor xenografts, respectively, and treated with ^177^Lu-lilotomab, ^177^Lu-cetuximab, unlabeled lilotomab, or rituximab. **(B)** Athymic and SCID mice bearing subcutaneous Ramos and DOHH2 cell tumor xenografts, respectively, underwent SPECT-CT imaging at 48h after injection of 2.5 mg/kg (athymic mice) or 0.5 mg/kg (SCID mice) ^177^Lu-lilotomab or ^177^Lu-rituximab. **(C)** After mouse sacrifice at various time points (1h, 24h, 48h, 72h, 144h; 5 mice/group/time point) following injection of 2.5 mg/kg (athymic mice) or 0.5 mg/kg (SCID mice) ^177^Lu-lilotomab or ^177^Lu-rituximab, tumors and organs were collected, weighed, radioactivity measured using a gamma counter, and the cumulated uptake of radioactivity calculated. **(D)** The number of CD20 and CD37 receptors per cell in Ramos and DOHH2 cells was determined by Scatchard analysis.

**Supplementary Figure 2.** **(A)** Proliferation of Ramos, Rec-1 and DOHH2 was assessed 72h after exposure or not to ^177^Lu-lilotomab (0-6 MBq/mL; 0-40 µg/mL) for 18h. **(B)** Proliferation rate of Raji and rituximab-resistant Raji cells (Raji 2R) was assessed at 72h and 144h after exposure or not to the indicated amounts of ^177^Lu-lilotomab (0-6 MBq/mL; 0-40 µg/mL) for 18h. **(C)** Immunogenic cell death was assessed by quantifying ATP and HMGB1 release from DOHH2 cells exposed to^177^Lu-lilotomab or rituximab. Results are the mean ± SD of three experiments performed in triplicate.

**Supplementary Figure 3.** Cell cycle analysis. Original flow cytometry images for Ramos, DOHH2, Rec-1, OCI-ly8, and U2932 cells exposed or not (NT) to MK-1775, PD166285, ^177^Lu-lilotomab or the combination of ^177^Lu-lilotomab+ MK-1775, or PD166285.

**Supplementary Figure 4. Cell cycle analysis.** Cell cycle phase distribution was assessed by flow cytometry. **(A)** DOHH2, **(B)** Ramos, and **(C)** Rec-1 cells were incubated with 40µg/mL of lilotomab or rituximab for 18h. The percentage of cells in G0/G1, S and G2/M phases was then determined (mean ± SD of three experiments performed in triplicate).

**Supplementary Figure 5. CDK1 phosphorylation and expression of proteins (WEE-1, MYT-1 and CDK7)** involved in G2/M cell cycle arrest was assessed by western blotting in OCI-Ly8 and U2932 cells (A), and in Ramos, Rec-1 and DOHH2 cells (B) respectively, during and after exposure to 0 (NT) and 6 MBq/mL of ^177^Lu-lilotomab for 18h.

**Supplementary Figure 6. Protein expression and role of G2/M cell cycle arrest inhibitors**

**(A)** Cell proliferation (flow cytometry) and CDK1 phosphorylation at Tyr15 and Thr14 (western blotting) were determined in U2932 cells during and after exposure to 0 (NT) and 6 MBq/mL ^177^Lu-lilotomab alone or with 1 µM MK-1775 (WEE-1 inhibitor) or PD-166285 (WEE-1 and MYT-1 inhibitor). **(B)** The percentage of U2932 cells in the G2/M cell cycle upon exposure or not (NT) to ^177^Lu-lilotomab or ^177^Lu-lilotomab + 1 µM MK-1775 (WEE-1 inhibitor) or PD-166285 (WEE-1 and MYT-1 inhibitor) was determined. **(C)** The percentage of Ramos, DOHH2, Rec-1, U2932, and OCI-ly8 cells in the G2/M cell cycle upon exposure or not (NT) to 1 µM MK-1775 (WEE-1 inhibitor) or PD-166285 (WEE-1 and MYT-1 inhibitor) was determined. Data are the mean ± SD of three independent experiments in triplicate; p ≤0.05, **p ≤0.01, ***p ≤0.001 (compared with non-treated (NT) control group).

**Supplementary Figure 7. Toxicity and efficacy of MK1775 and PD166285.** Mass of **(A)** Ramos and (B) OCI-Ly8 cell xenografts collected at day 18 and 42 from mice treated with NaCl, 2.5 mg/kg rituximab or lilotomab, ^177^Lu-lilotomab (250MBq/kg or 500MBq/kg), MK-1775 (30mg/kg twice a day for 5 days), or the combination ^177^Lu-lilotomab (250MBq/kg) + MK1775. (C) Weekly monitoring of body weight of athymic mice bearing subcutaneous Ramos and OCI-Ly8 cell xenografts and treated with ^177^Lu-lilotomab (250MBq/kg), MK1775 or the combination**.**

**Supplementary Figure 8. Efficacy of ^177^Lu-lilotomab on CD20^+^/kappa^+^ cells grown from patients’ biopsies. (A)** Example of flow cytometric analysis of cells isolated from a biopsy of a patient with DLBCL1 using antibodies against CD45, CD3, CD20 receptors and Ig light chain (kappa). Cells were activated with 50 ng/mL CD40L (His-tagged) and 5 µg/mL anti-His-tag antibody. **(B)** Flow cytometric analysis of the proportion of CD20^+^/kappa^+^ cells in tumor samples from patients with DLBCL or FL. Cells were exposed or not (NT) to the indicate concentrations of ^177^Lu-lilolotmab for 18h and the analysis was done 3 days later (day 4).

**Supplementary Figure 9. Theoretical and experimental efficacy of ^177^Lu-lilotomab alone or combined with G2/M arrest inhibitors in tumor samples from patients with NHL**

Theoretical (using the Bliss independence mathematical model) and experimental additive anti-proliferative effects of ^177^Lu-lilotomab + MK1775 or PD 166285 (18h incubation) on tumor cells isolated from four different patients. Analysis was done on day 1 and 4 post-treatment.
