# Supplementary figures and images for: The therapeutic effectiveness of 177Lu-lilotomab in B-cell non-Hodgkin lymphoma involves modulation of G2/M cell cycle arrest
Source: Leukemia. 2019 Dec 13;34(5):1315–28. doi: 10.1038/s41375-019-0677-4 (PMC7192854; doi:10.1038/s41375-019-0677-4)

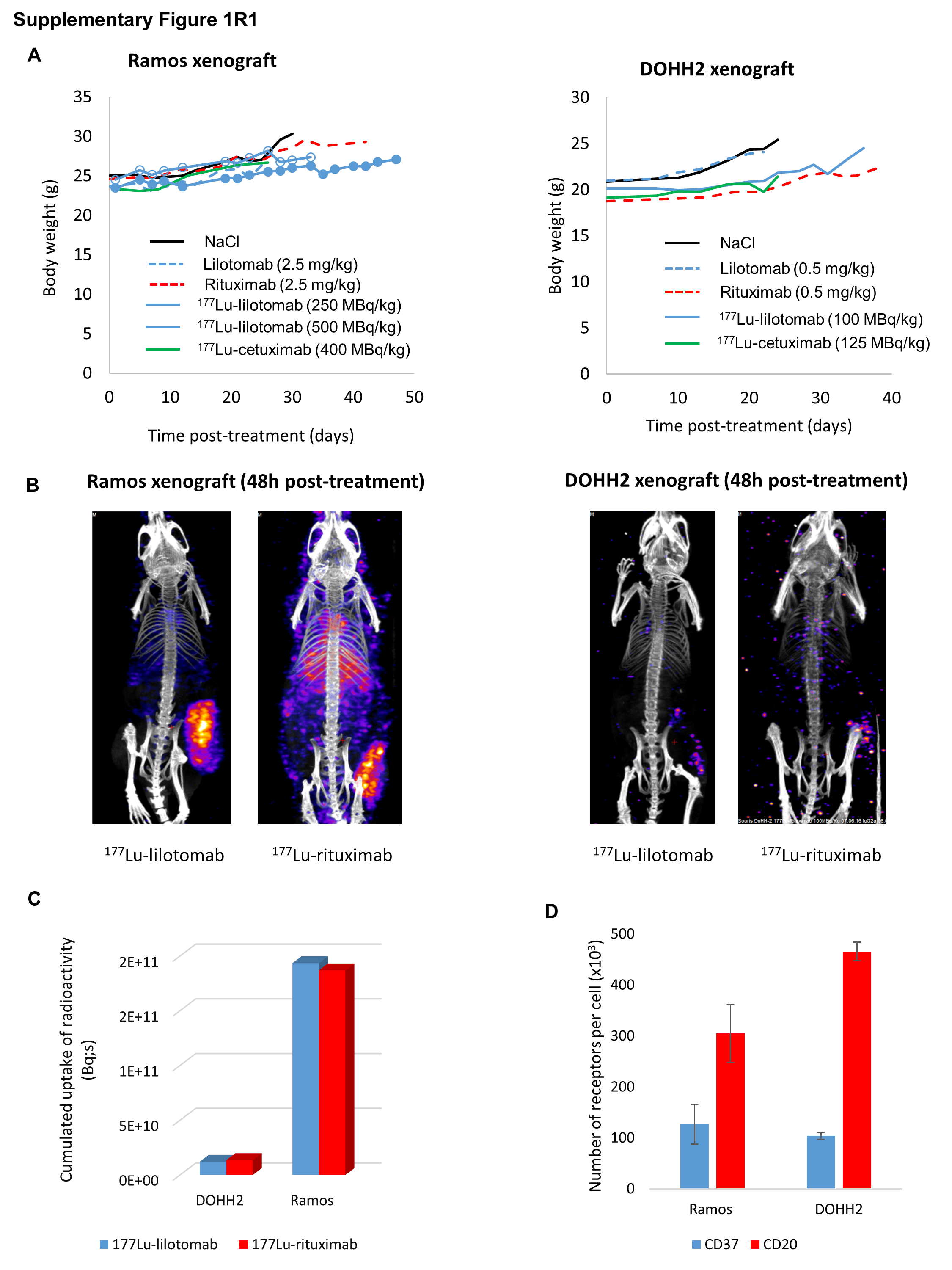

Supplement: Supplementary file 2 — Supplementary Figure 1 [file 41375_2019_677_MOESM2_ESM.tif]

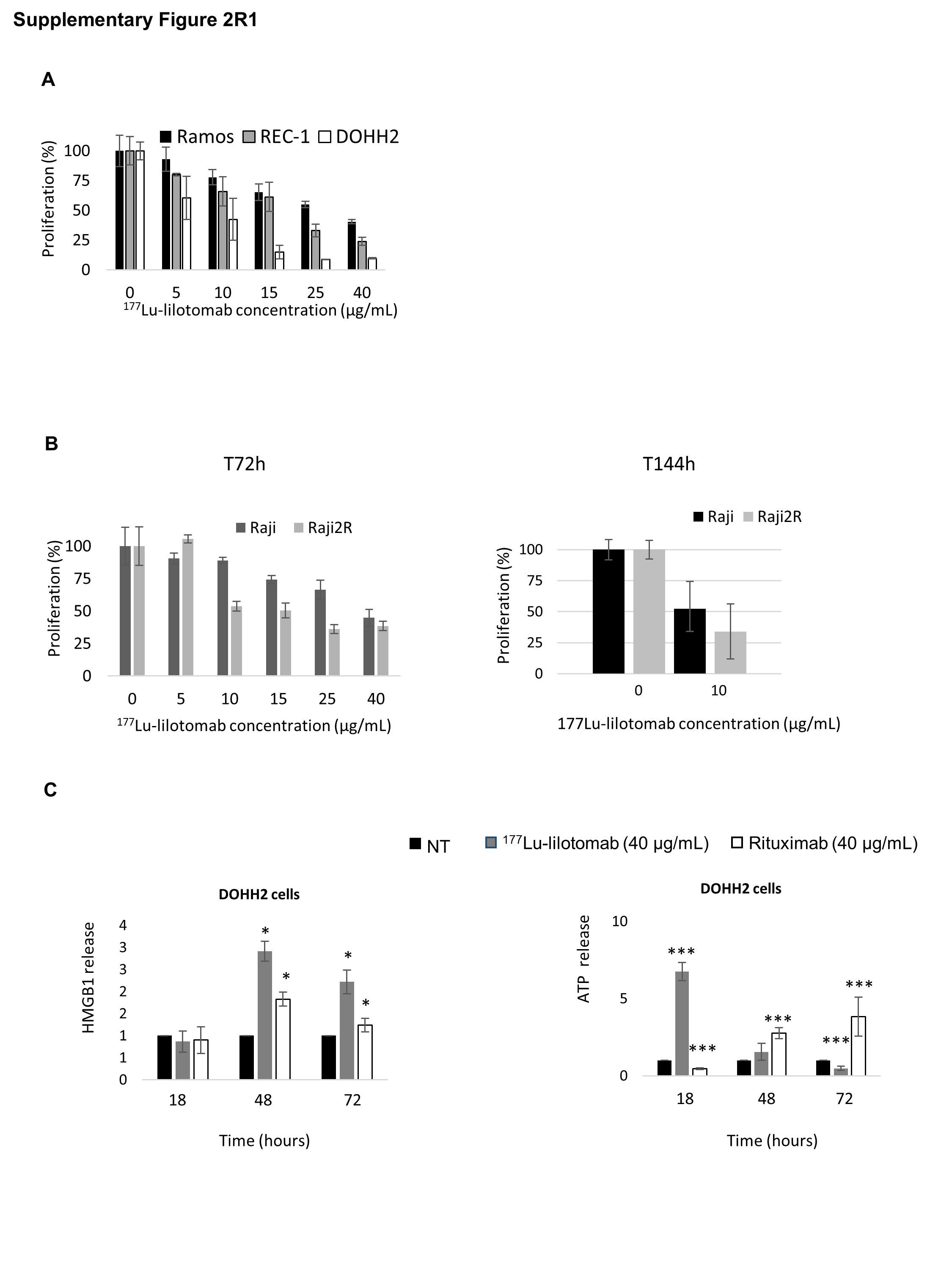

Supplement: Supplementary file 3 — Supplementary Figure 2 [file 41375_2019_677_MOESM3_ESM.tif]

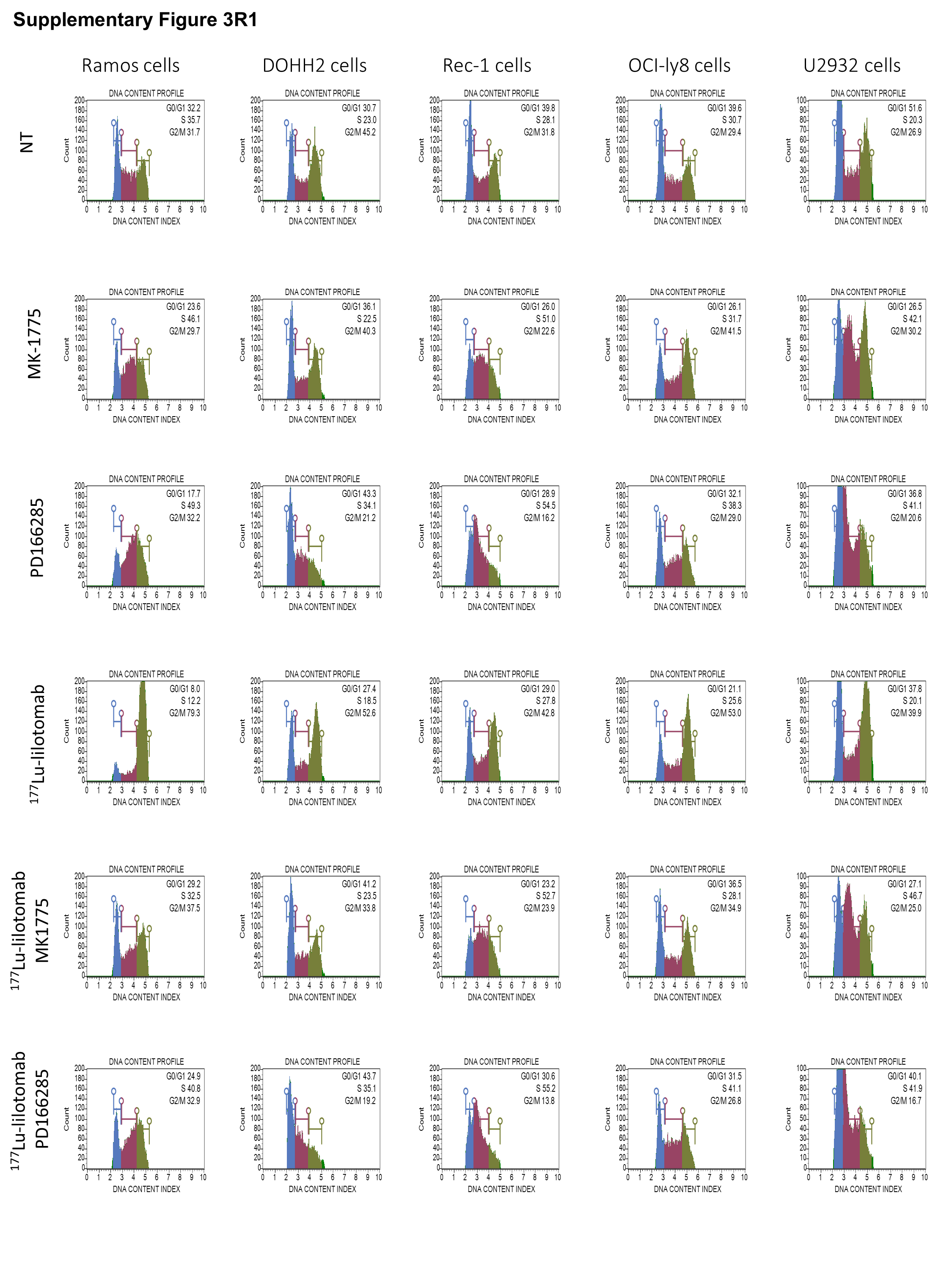

Supplement: Supplementary file 4 — Supplementary Figure 3 [file 41375_2019_677_MOESM4_ESM.tif]

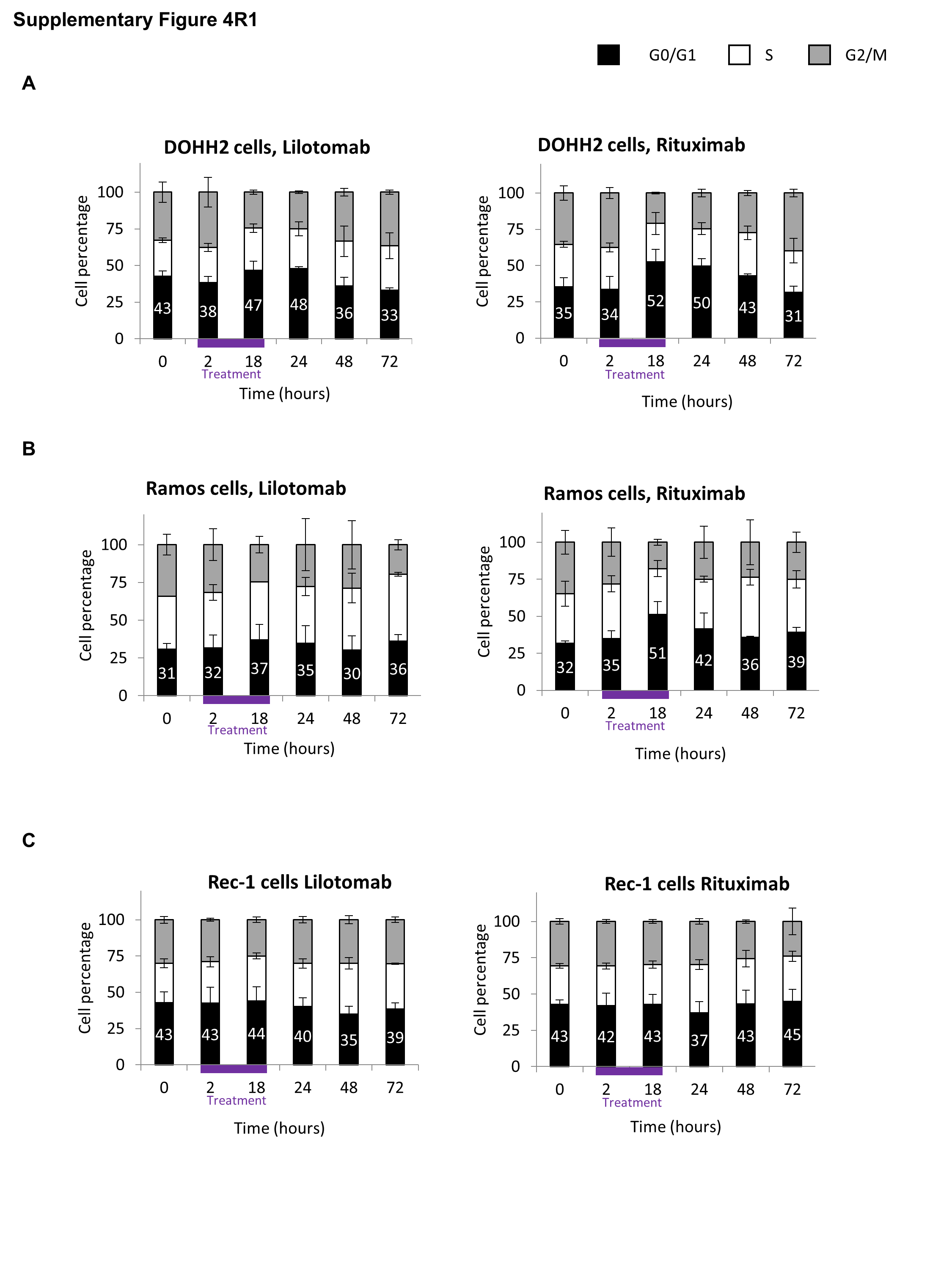

Supplement: Supplementary file 5 — Supplementary Figure 4 [file 41375_2019_677_MOESM5_ESM.tif]

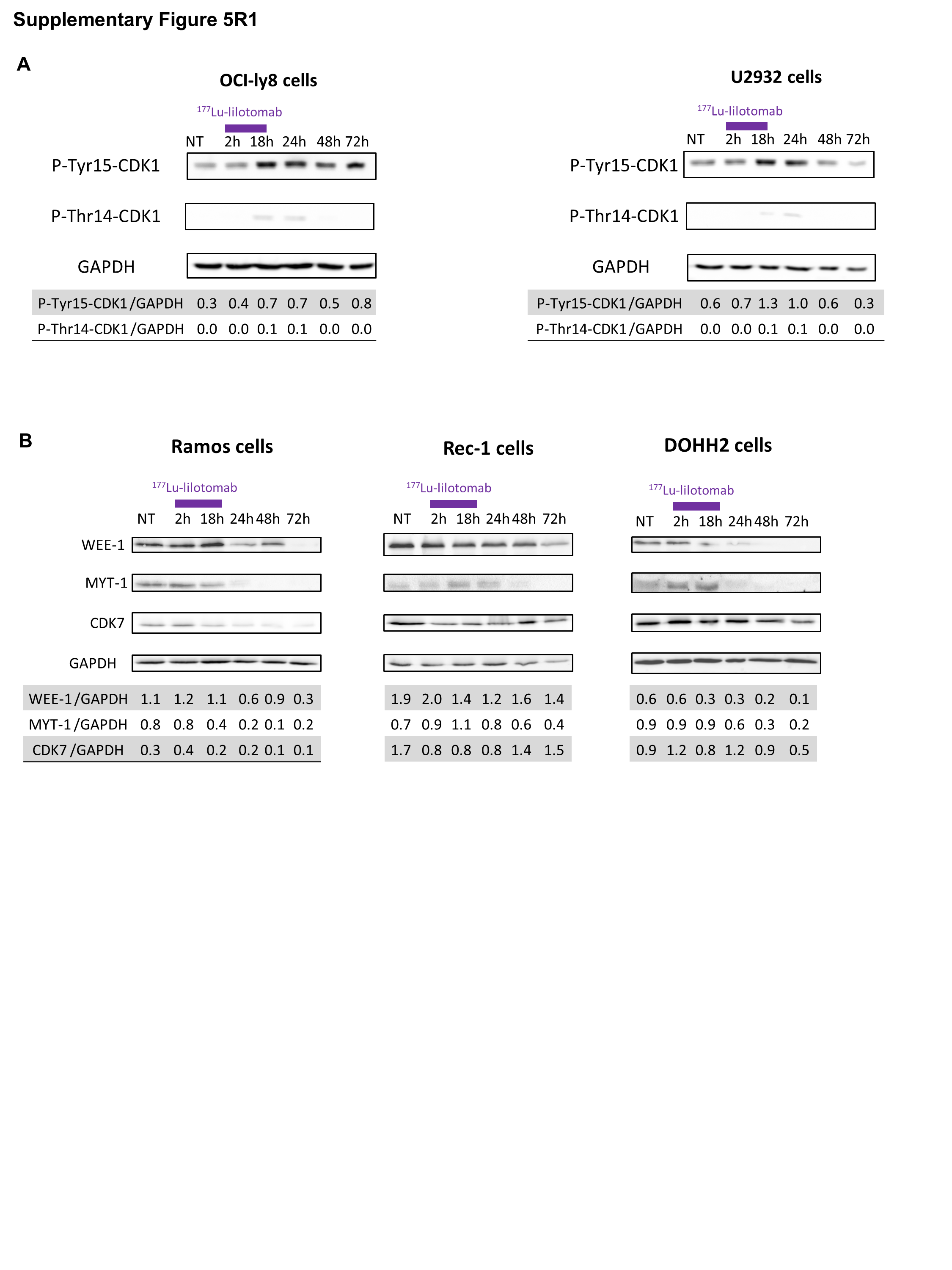

Supplement: Supplementary file 6 — Supplementary Figure 5 [file 41375_2019_677_MOESM6_ESM.tif]

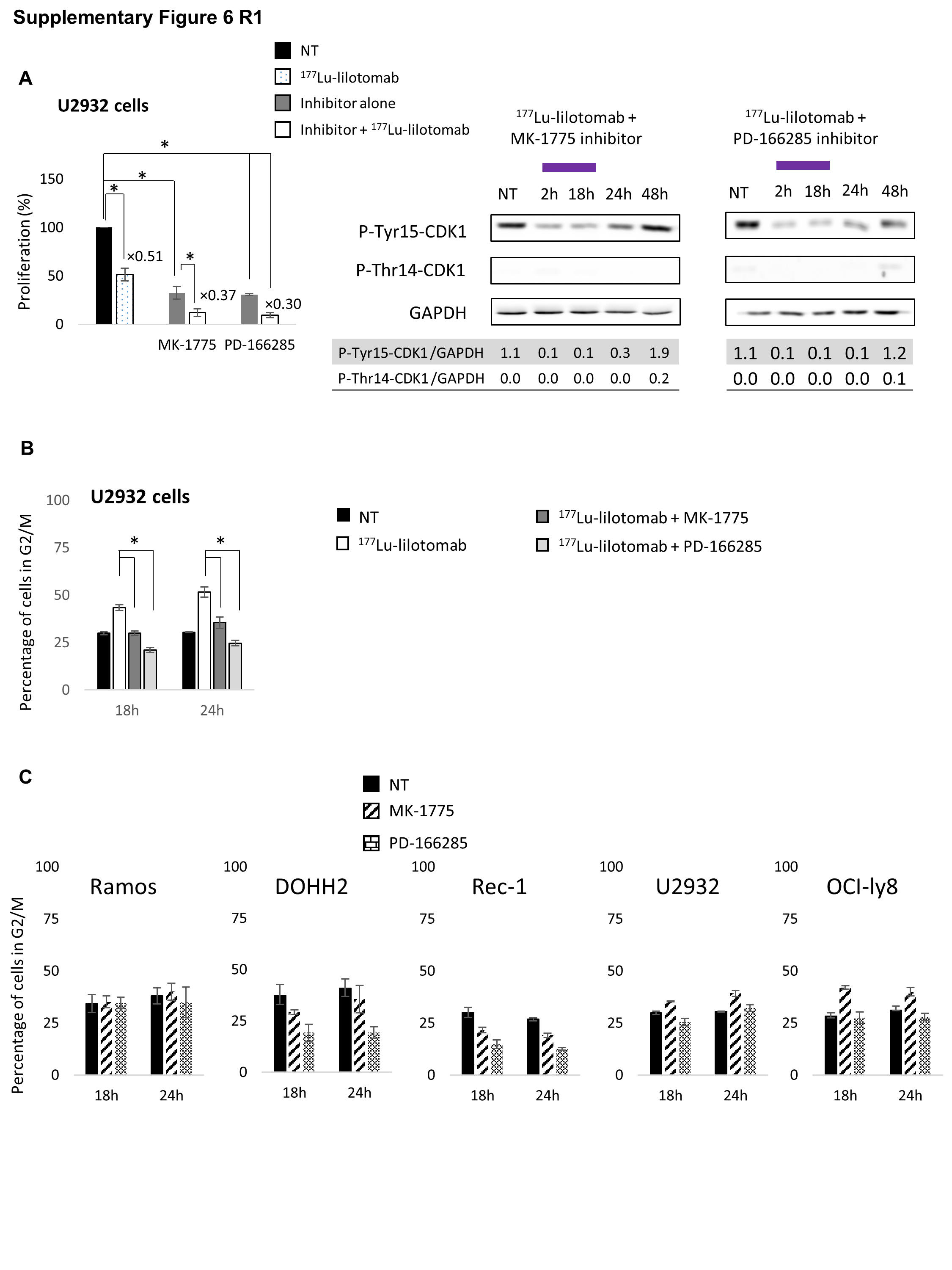

Supplement: Supplementary file 7 — Supplementary Figure 6 [file 41375_2019_677_MOESM7_ESM.tif]

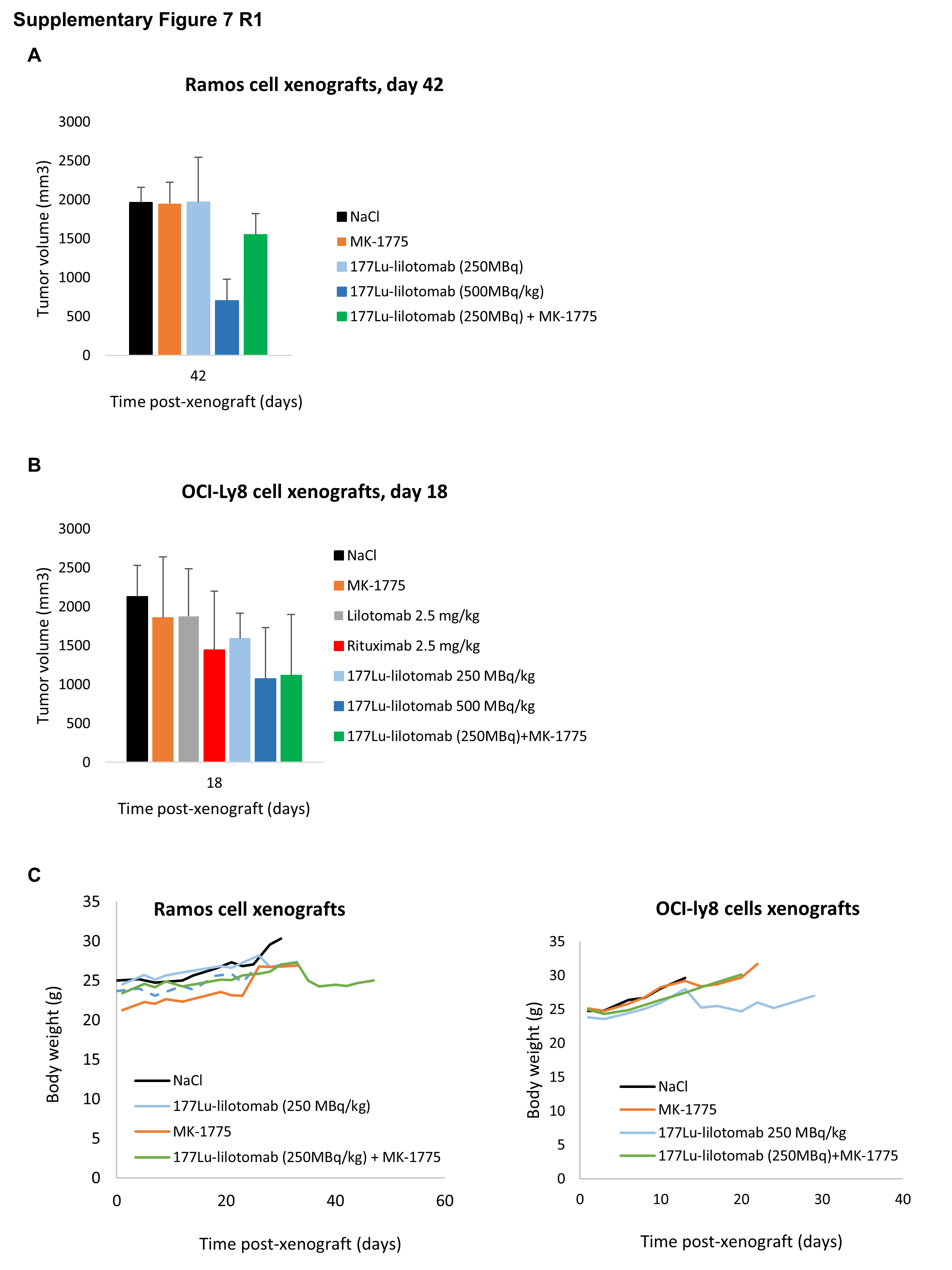

Supplement: Supplementary file 8 — Supplementary Figure 7 [file 41375_2019_677_MOESM8_ESM.tif]

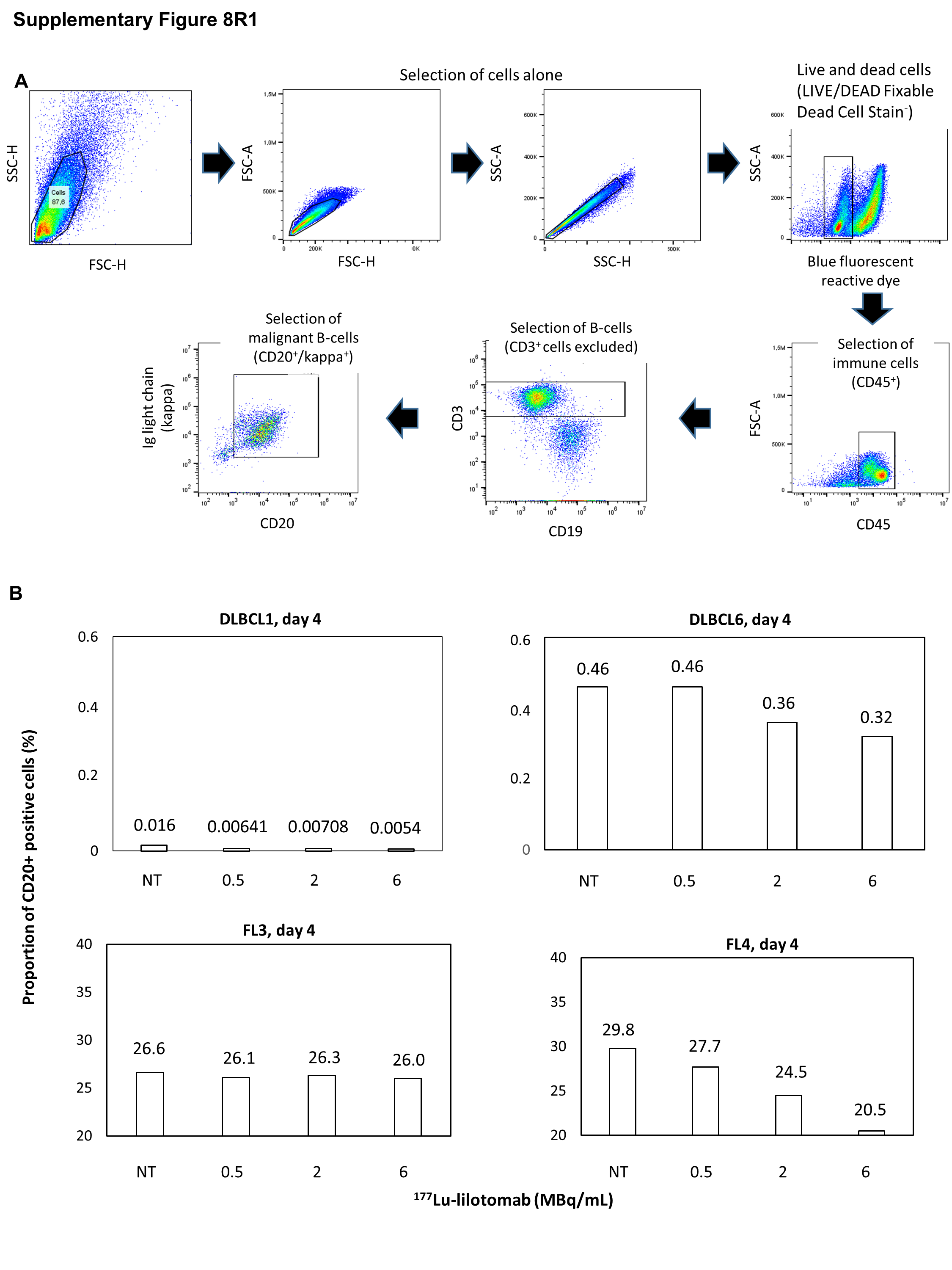

Supplement: Supplementary file 9 — Supplementary Figure 8 [file 41375_2019_677_MOESM9_ESM.tif]

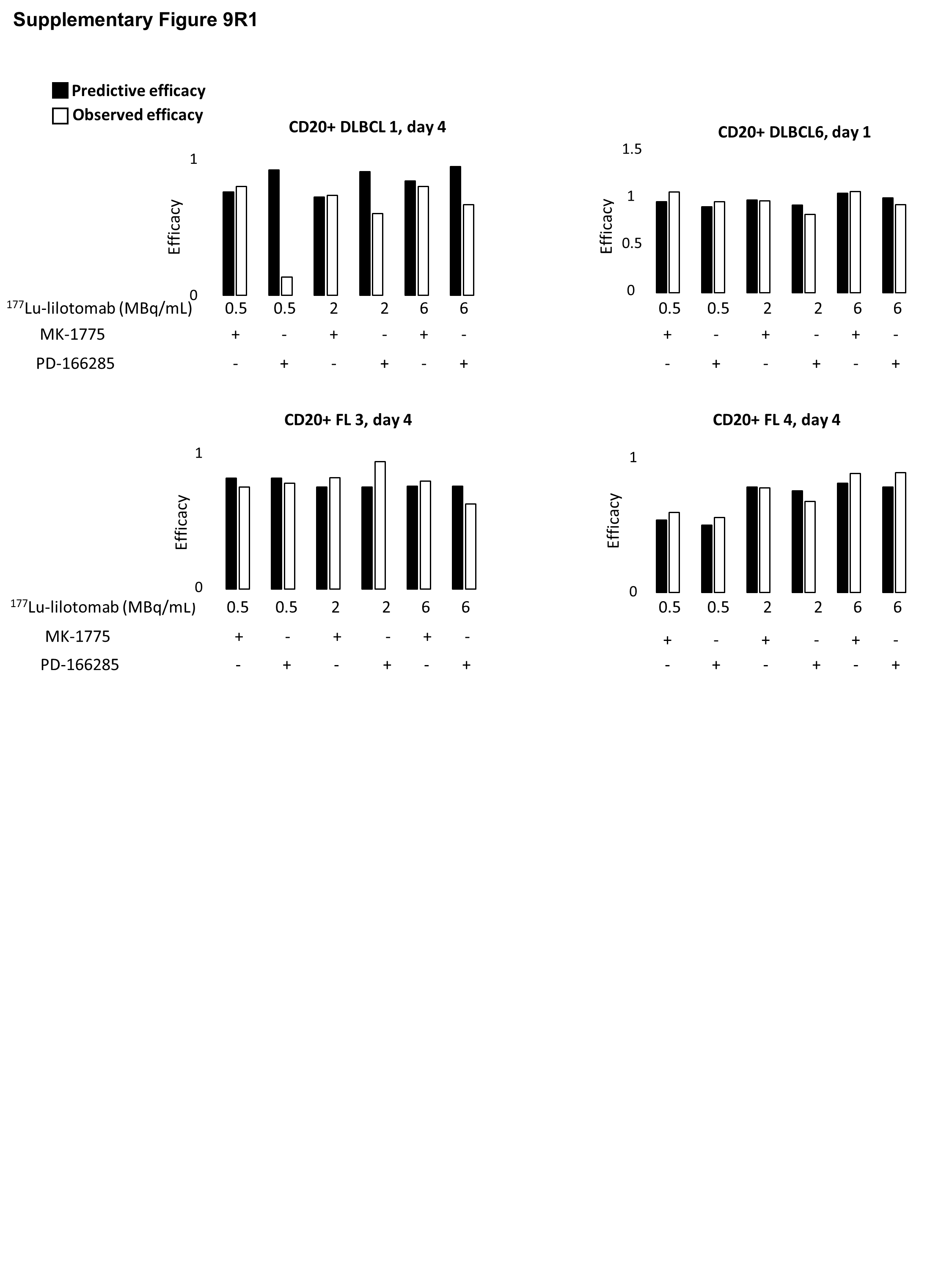

Supplement: Supplementary file 10 — Supplementary Figure 9 [file 41375_2019_677_MOESM10_ESM.tif]
