# Supplementary material for: The therapeutic effectiveness of 177Lu-lilotomab in B-cell non-Hodgkin lymphoma involves modulation of G2/M cell cycle arrest
Source: Leukemia. 2019 Dec 13;34(5):1315–28. doi: 10.1038/s41375-019-0677-4 (PMC7192854; doi:10.1038/s41375-019-0677-4)
Supplement: Supplementary file 11 — Supplementary Table [file 41375_2019_677_MOESM11_ESM.docx]

Supplementary Table 1: Number and percentage of living cells collected from patient biopsies
