# Supplementary material for: The therapeutic effectiveness of 177Lu-lilotomab in B-cell non-Hodgkin lymphoma involves modulation of G2/M cell cycle arrest
Source: Leukemia. 2019 Dec 13;34(5):1315–28. doi: 10.1038/s41375-019-0677-4 (PMC7192854; doi:10.1038/s41375-019-0677-4)
Supplement: Supplementary file 12 — Supplementary Methodology [file 41375_2019_677_MOESM12_ESM.docx]

**Supplementary Methodology. Pichard *et al.***

**Cell lines and cell surface receptor quantification**

The Ramos and Raji (Burkitt’s lymphoma, BL), DOHH2 (transformed FL), U2932 (DLBCL) and Rec-1 (mantle cell lymphoma) cell lines were obtained from ATCC/ICLC, ECACC and DSMZ. The OCI-Ly8 (DLBCL) cell line was from the Institute of Oncology Research, Bellinzona, Switzerland, and rituximab-resistant Raji cells (RAJI2R) that express CD20 and CD37 were kindly provided by the Roswell Park Cancer Institute, New York, US ([33](#_ENREF_33)). Cells were grown at 37°C in a humidified atmosphere of 95% air/5% CO_2_ in RPMI medium supplemented with 10% heat-inactivated fetal bovine serum (FBS), 0.1 U/ml penicillin and 100 µg/ml streptomycin. Mycoplasma contamination was routinely tested using the MycoTect assay from Life technologies (Thermo Fisher Scientific, Waltham, MA) and recently authenticated.

Typically, 5×10^5^ cells grown in tubes containing 100µL culture medium were incubated with increasing amounts (0-6.25 nM; average specific activity of 200 MBq/mg) of ^177^Lu-lilotomab or ^177^Lu-rituximab at room temperature for 1h. Radioactivity was gamma-counted before washing the cells twice with PBS to remove unbound radioactivity. Cells were then resuspended in 0.2 mL of culture medium and an aliquot was used for cell counting and radioactivity measurement. The ratio between bound and free radioactivity was determined, and expressed as a function of the bound radioactivity.

**Biodistribution of radiolabeled antibodies and tumor uptake**

Mice bearing subcutaneous Ramos or DOHH2 cell xenografts received one intravenous injection of ^177^Lu-lilotomab, ^177^Lu-rituximab or ^177^Lu-cetuximab at antibody concentrations similar to those used for therapy. At various time points (1h, 24h, 48h, 72h, 144h) after injection, five mice in each group were sacrificed and tumors and organs collected, weighed and radioactivity measured using a gamma counter. The percentage of the injected activity per gram of tissue was then expressed for all samples (%IA/g) and used to calculate the cumulative number of decays (Bq.s) per organ and tumor.

**Clonogenic survival and proliferation assays**

A standard clonogenic cell survival assay was used to assess ^177^Lu-mAb cytotoxicity *in vitro* in Ramos and DOHH2 cells (but not in Rec-1 cells because they do not form colonies). Typically, 1×10^6^ cells/mL were grown in 12-well plates containing 1 mL of RPMI medium and incubated with increasing activities (0-6 MBq/mL) of ^177^Lu-mAbs at 37°C/5% CO_2_ for 18h. Next, cells were collected, centrifuged, and washed twice with medium before resuspension in 5 mL of RPMI medium for counting. Then, 1 500 to 45 000 cells were mixed with 4.5 mL of MethoCult^®^ medium (StemCell Technologies, Grenoble, France) and seeded (1.5 mL/dish). The number of seeded cells/dish ranged between 500 and 15 000, depending on the antibody and test activity. Cells were cultured for 12 to 16 days (d). Colonies containing 50 or more cells were scored, and the surviving fraction calculated relative to non-treated (NT) cells.

For cell proliferation assessment, the number of living cells at 126h post-treatment was determined in 100µL cell suspensions using the Count & Viability Assay Kit and the Muse^®^ Cell Analyzer (Merck Millipore, Molsheim, France). Proliferation was calculated as the percentage of the value in NT cells set to 100%. Experiments were repeated at least three times in triplicate.

**Apoptosis measurement**

1×10^6^ Ramos, Rec-1 and DOHH2 cells were grown in 12-well plates containing 1 mL of medium and incubated with 0 and 6 MBq/mL of ^177^Lu-mAbs or the overestimated corresponding amounts (0 and 40 µg/mL) of unlabeled antibodies for 18h. Cells were harvested at 0h, 2h, 18h, 1d, 2d, and 3d, and apoptotic cells were detected using the Muse^®^ Annexin V and Dead Cell Assay Kit with 7-AAD (Merck Millipore) and a Muse^®^ flow cytometer.

**Micronucleus Assay**

Micronucleus formation was tested using the method described in (*21*). Briefly, 1 × 10^6^ Ramos or DOHH2 cells were grown in triplicate in 12-well plates (1 ml of medium/well). Cells were incubated with 6 MBq/mL ^177^Lu-lilotomab or 40 µg/mL rituximab for 18h. After treatment, cells were washed twice with PBS and cytochalasin B (2µg/mL; Sigma Aldrich, St Louis, Mo) was added to the culture medium to block cytokinesis for 48h. Cells were then fixed in acetic acid: ethanol (1:3) before staining with Vectashield (Vector laboratories, San Diego, CA, USA) and analysis under a fluorescence microscope. At least 100 cells were examined, and only micronuclei in binucleated cells were considered. Experiments were repeated three times in triplicate.

**Calreticulin exposure**

1× 10^6^ cells/mL were incubated with rituximab (40µg/mL) or 6 MBq/mL ^177^Lu-lilotomab for 18h. Cells were collected, washed in cold PBS/10% FBS, and stained with DAPI (Beckman Coulter) for 30min. After washing in PBS/10% FBS, cells were fixed with 0.25% paraformaldehyde for 5min. After washing, cells were incubated with an anti–calreticulin antibody conjugated to Alexa Fluor 647 (ab196159, Abcam) for 1h, followed by washing, and fixation in 1% paraformaldehyde for 5min. Calreticulin exposure was assessed using a Cytoflex^®^ Cytometer (Beckman Coulter). The fluorescent intensity of calreticulin-positive cells was gated relative to DAPI–negative cells.

**ATP and HMGB1 release**

ATP and HMGB1 release in cell supernatant was quantified in 1× 10^6^ cells/mL after incubation with rituximab (40µg/mL) or 6 MBq/mL ^177^Lu-lilotomab for 18h and two washes with PBS. Supernatants were collected and centrifuged to remove dying cells. ATP release was measured with the ATPlite Luminescence Assay System (PerkinElmer), according to the manufacturer's instructions. HMGB1 release was quantified with the HMGB1 ELISA Kit (IBL International), according to the manufacturer's instructions.

**Cell cycle analysis**

Cell cycle was assessed in 1×10^6^ cells grown in 12-well plates and exposed to 0 and 6 MBq/mL of ^177^Lu-lilotomab, or to the slightly overestimated corresponding range (0 and 40 µg/mL) of lilotomab or rituximab for 18h. Cells were harvested at 0h, 2h, 18h, 1d, 2d, 3d, and fixed in 70% ethanol at −20 °C for at least 3h. After staining with the Muse^®^ Cell Cycle Assay Kit (Merck Millipore, Molsheim, France) using propidium iodide in the dark at room temperature for 30min, cell cycle distribution was analyzed using a Muse^®^ flow cytometer. The percentage of cells in G0/G1, S and G2/M was calculated (mean of three experiments in triplicate). The effect of WEE-1 and MYT-1 kinase inhibitors on the cell cycle was also assessed.

**WEE-1 and MYT-1 inhibitors and western blotting**

Protein expression was assessed by western blotting using 1×10^6^ cells/mL grown in 25 cm² flasks after exposure to 0 and 6 MBq/mL of ^177^Lu-lilotomab for 18h. For protein expression analysis after incubation with 1 µM of the selective WEE-1 kinase inhibitor MK-1775 (Selleckchem, Houston, USA) or of the dual WEE-1/MYT-1 inhibitor PD-166285 **(**EMD Merck Millipore/Calbiochem, Molsheim, France), the tested activities were 6 MBq/mL for Ramos, 2 MBq/mL for Rec-1, U2932 and OCI-Ly8, and 0.5 MBq/mL for DOHH2 cells.

Cells were harvested at 0h, 2h, 18h, 1d and 2d, rinsed and lysed in RIPA buffer (Santa Cruz; Santa Cruz, USA) at 4°C for 30min. After centrifugation and supernatant collection, 30 µg of proteins were separated by SDS-PAGE (12% poly-acrylamide gels) and electrotransferred onto nitrocellulose membranes. Membranes were incubated with anti-CDK1, anti-p-CDK1 (Tyr15) (clone 10A11), anti-p-CDK1 (Thr14), anti-p-CDK1 (Thr161), anti-CDK7, anti-WEE-1 (clone D10D2), anti-MYT-1, and anti-human GAPDH (1/1000, Cell Signaling Technologies, Leiden, The Netherlands) primary antibodies. Secondary antibodies were horseradish peroxidase-conjugated anti-mouse (Jackson ImmunoResearch, West Grove, USA) and anti-rabbit (Cell Signaling Technology, Danvers, USA) IgGs. Proteins were detected using an enhanced chemiluminescence system according to the manufacturer's instructions (Clarity™, BioRad, Marnes-La-Coquette France). Protein expression was quantified with a PXi analyzer (Ozyme, St Quentin en Yvelines, France).
