# Supplementary material for: The therapeutic effectiveness of 177Lu-lilotomab in B-cell non-Hodgkin lymphoma involves modulation of G2/M cell cycle arrest
Source: Leukemia. 2019 Dec 13;34(5):1315–28. doi: 10.1038/s41375-019-0677-4 (PMC7192854; doi:10.1038/s41375-019-0677-4)
Supplement: Supplementary file 13 — Dataset 1 [file 41375_2019_677_MOESM13_ESM.pptx]

## Slide 1
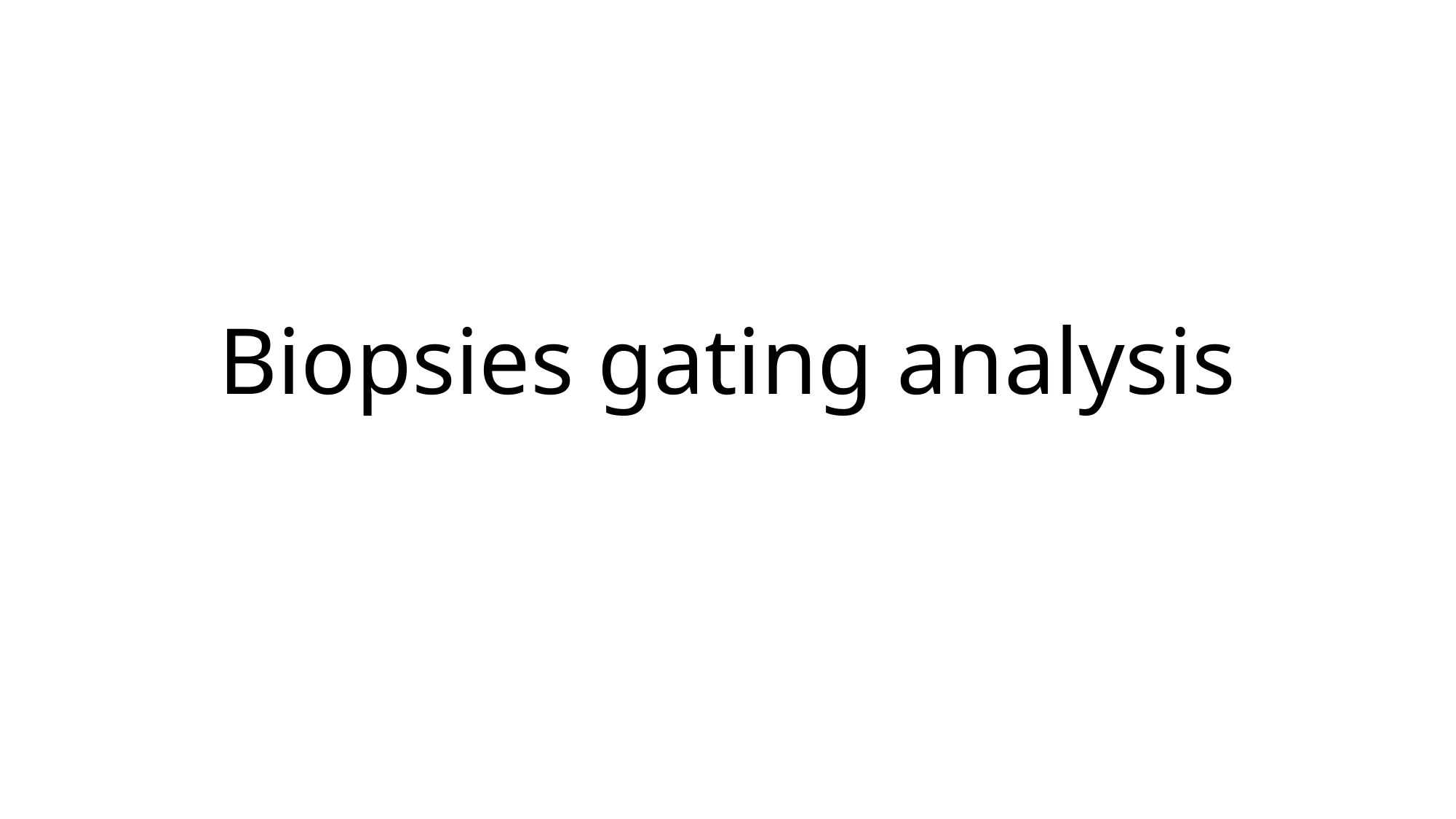

# Biopsies gating analysis

## Slide 2
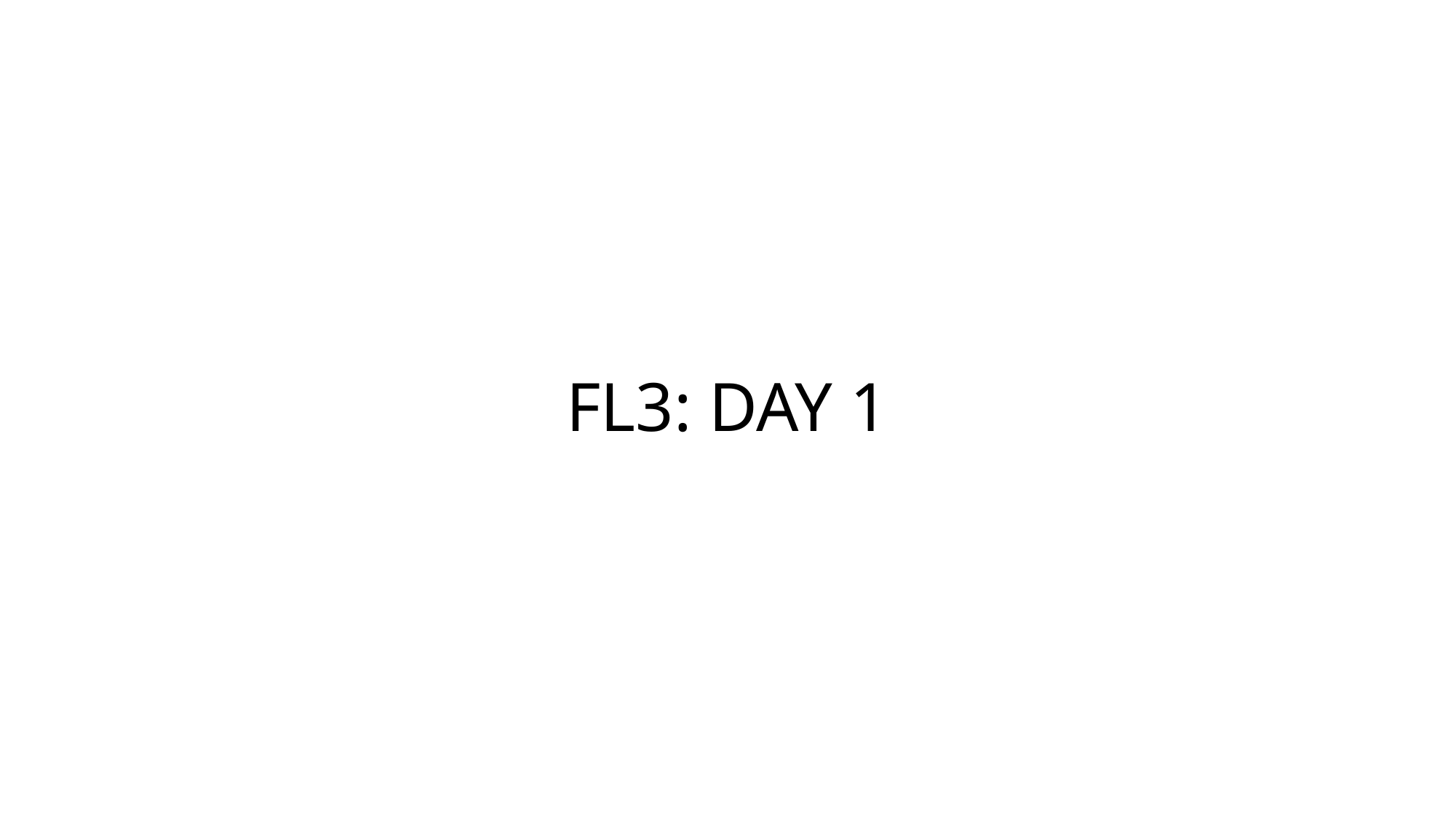

# FL3: DAY 1

## Slide 3
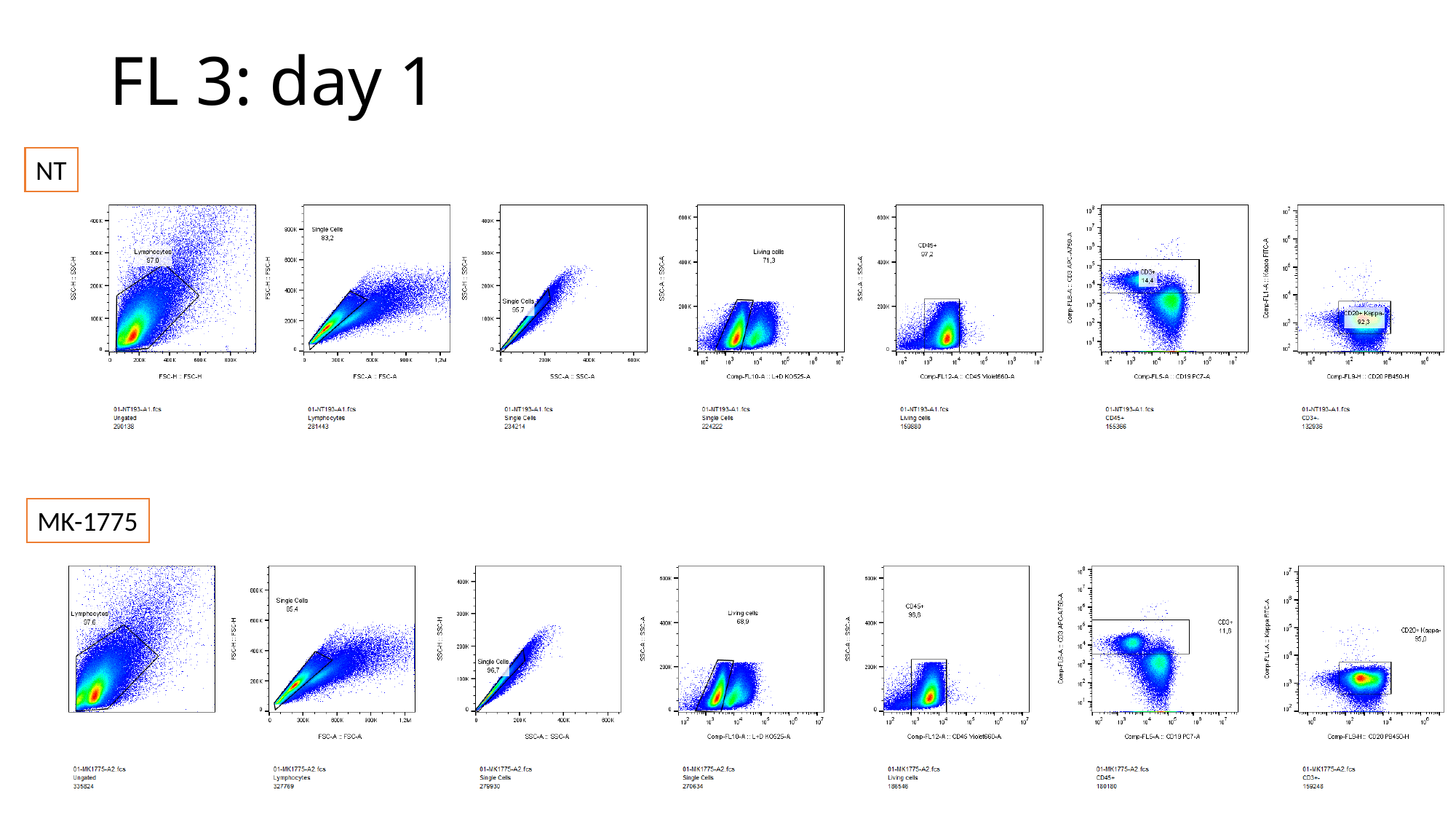

# FL 3: day 1
NT
MK-1775

## Slide 4
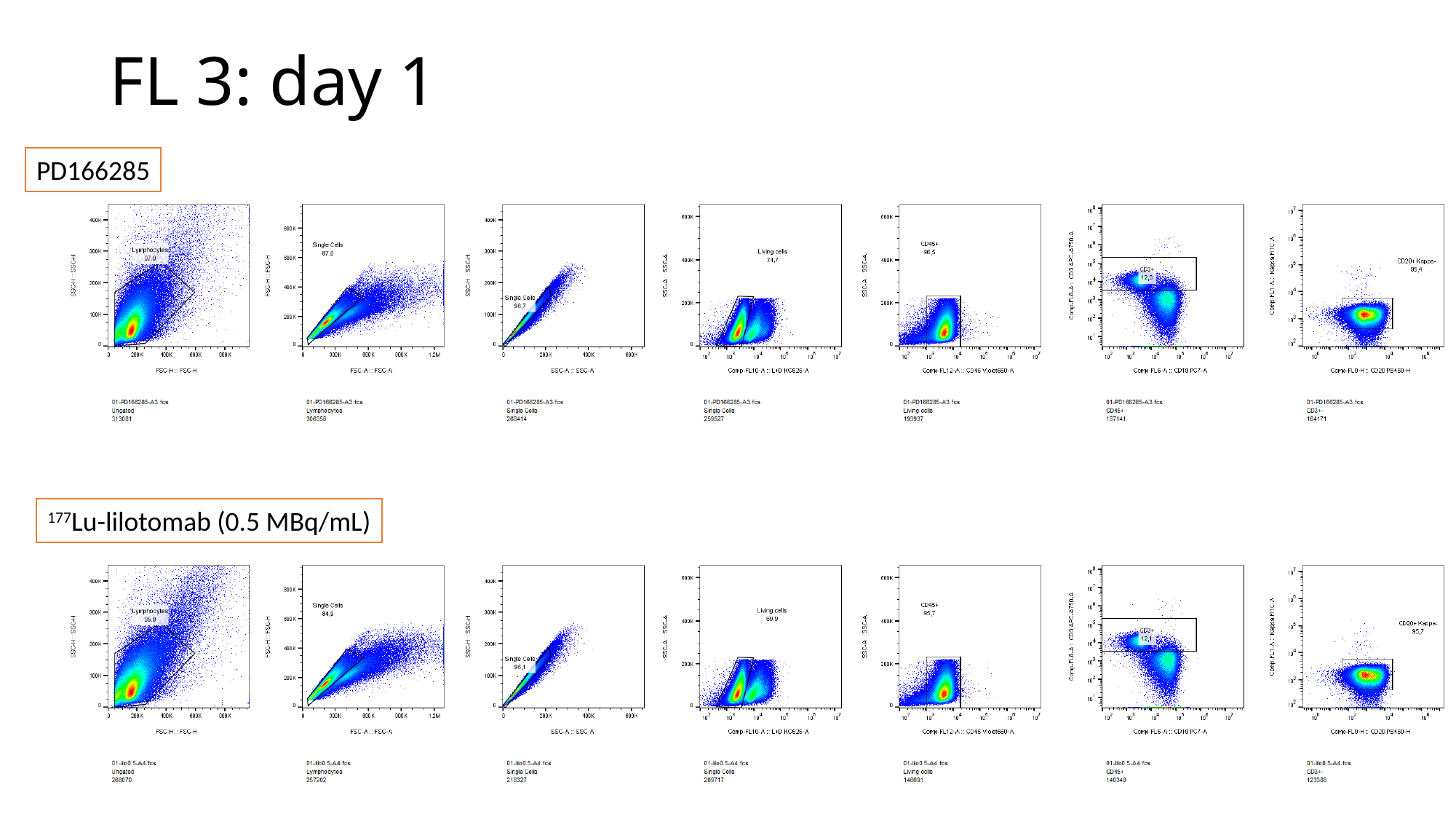

# FL 3: day 1
PD166285
177Lu-lilotomab (0.5 MBq/mL)

## Slide 5
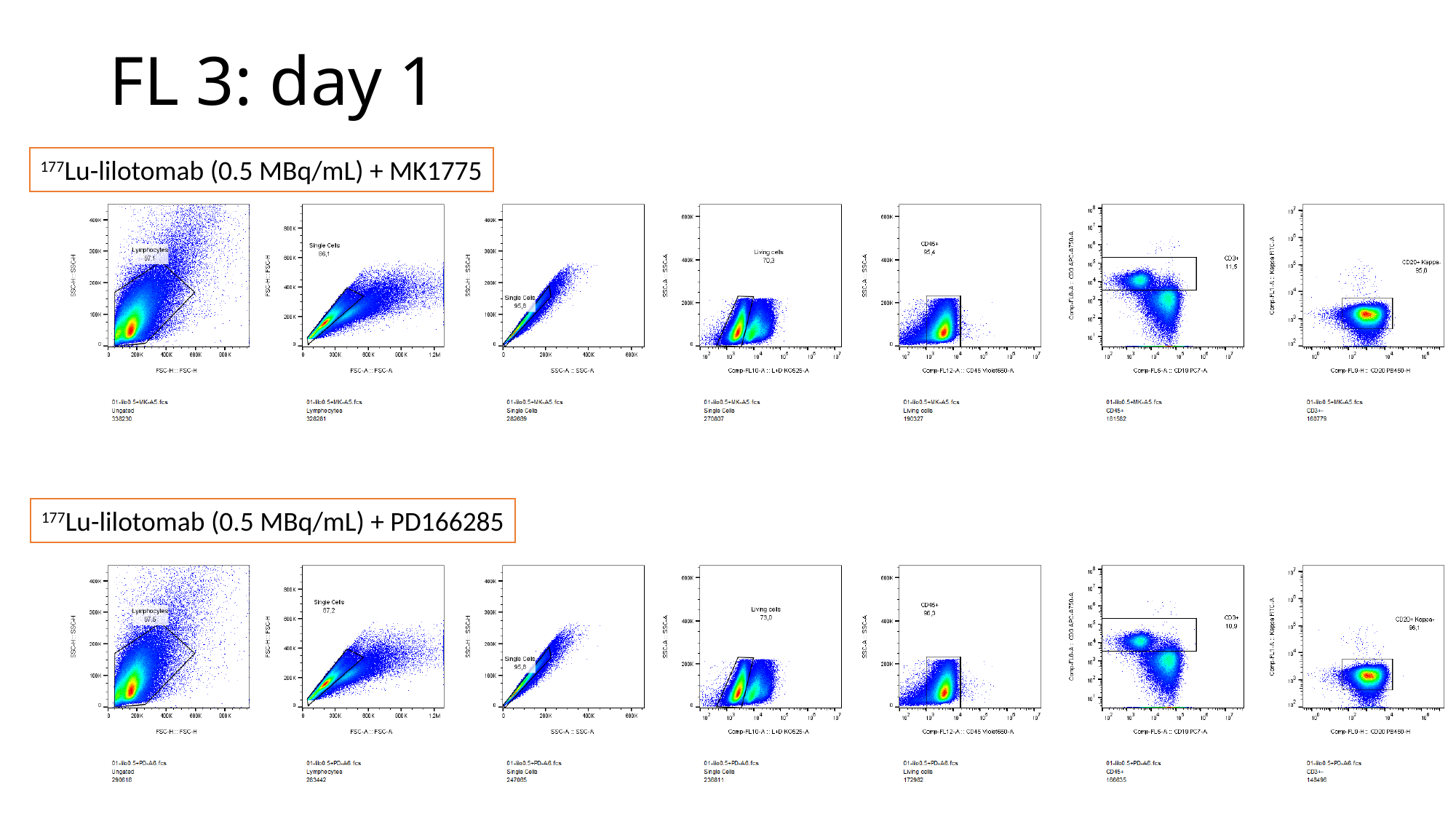

# FL 3: day 1
177Lu-lilotomab (0.5 MBq/mL) + MK1775
177Lu-lilotomab (0.5 MBq/mL) + PD166285

## Slide 6
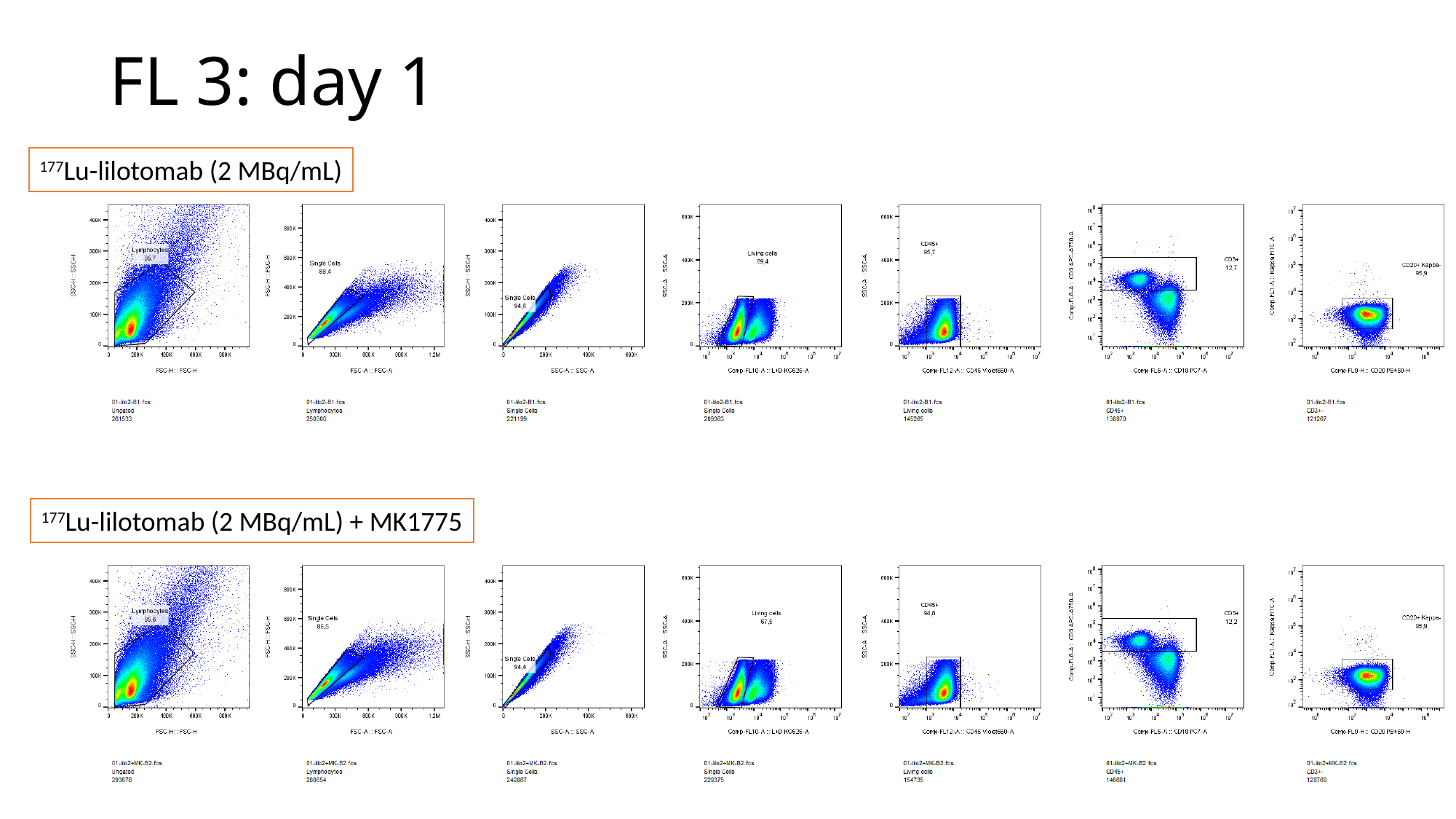

# FL 3: day 1
177Lu-lilotomab (2 MBq/mL)
177Lu-lilotomab (2 MBq/mL) + MK1775

## Slide 7
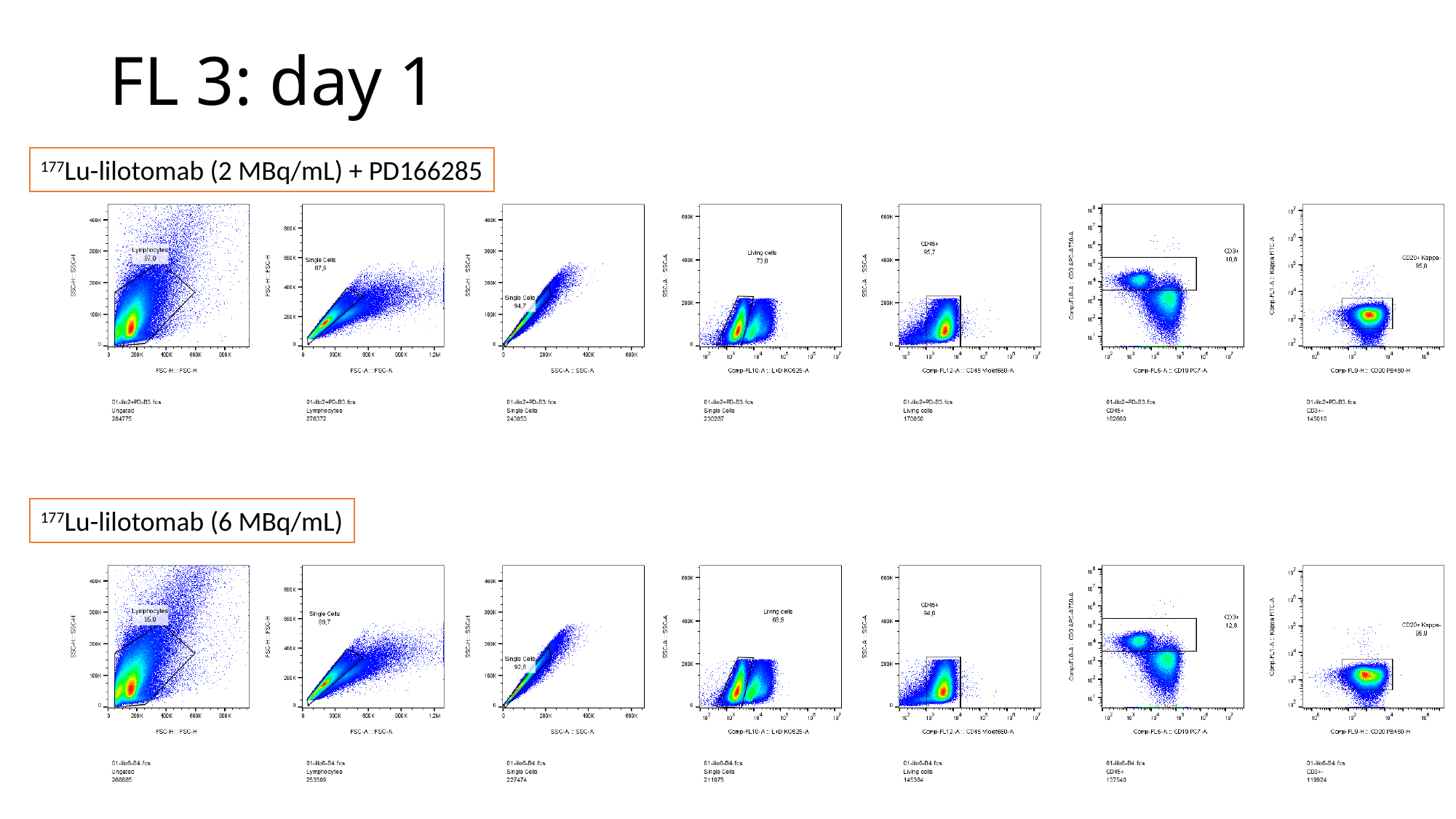

# FL 3: day 1
177Lu-lilotomab (2 MBq/mL) + PD166285
177Lu-lilotomab (6 MBq/mL)

## Slide 8
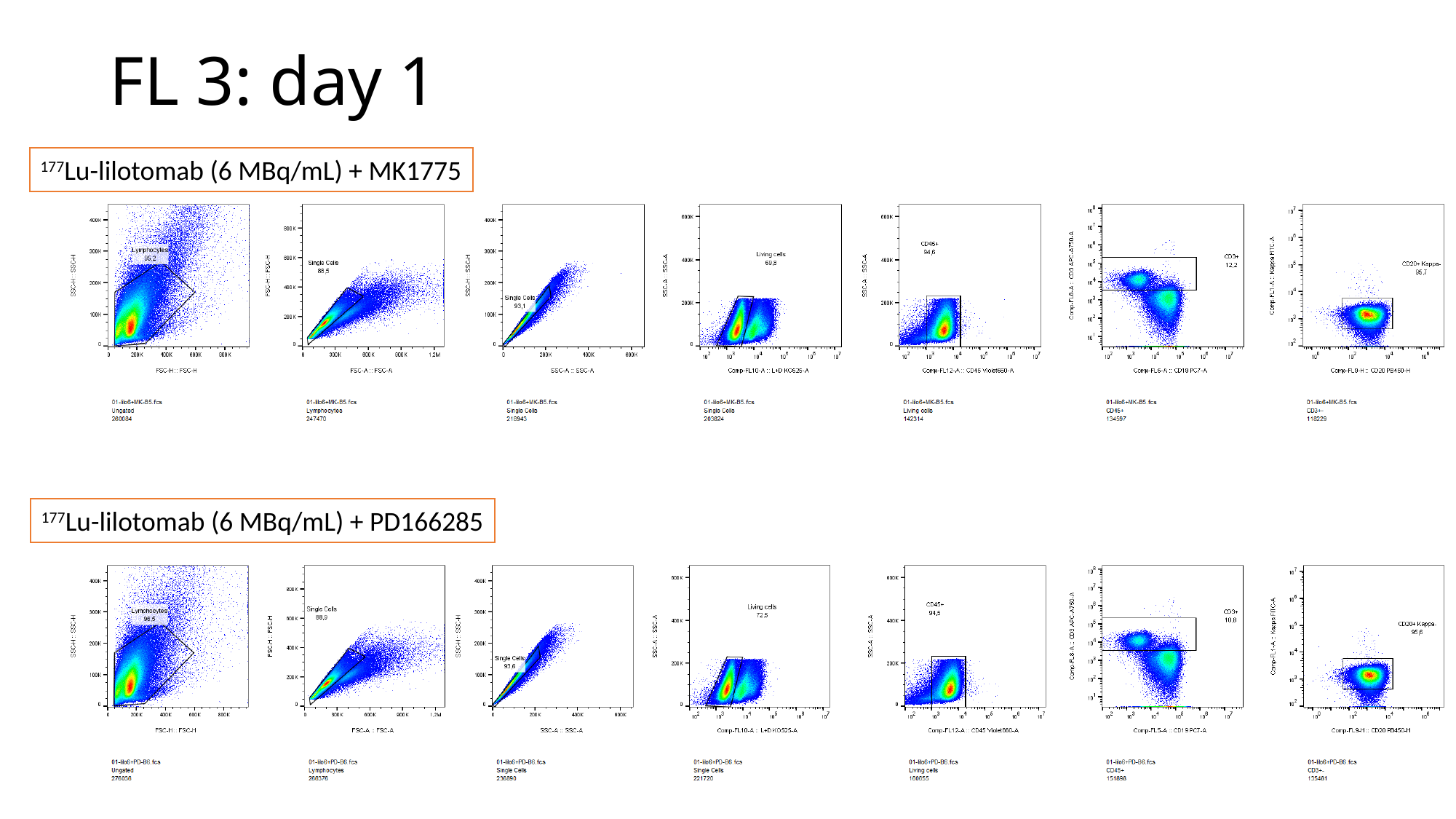

# FL 3: day 1
177Lu-lilotomab (6 MBq/mL) + MK1775
177Lu-lilotomab (6 MBq/mL) + PD166285

## Slide 9
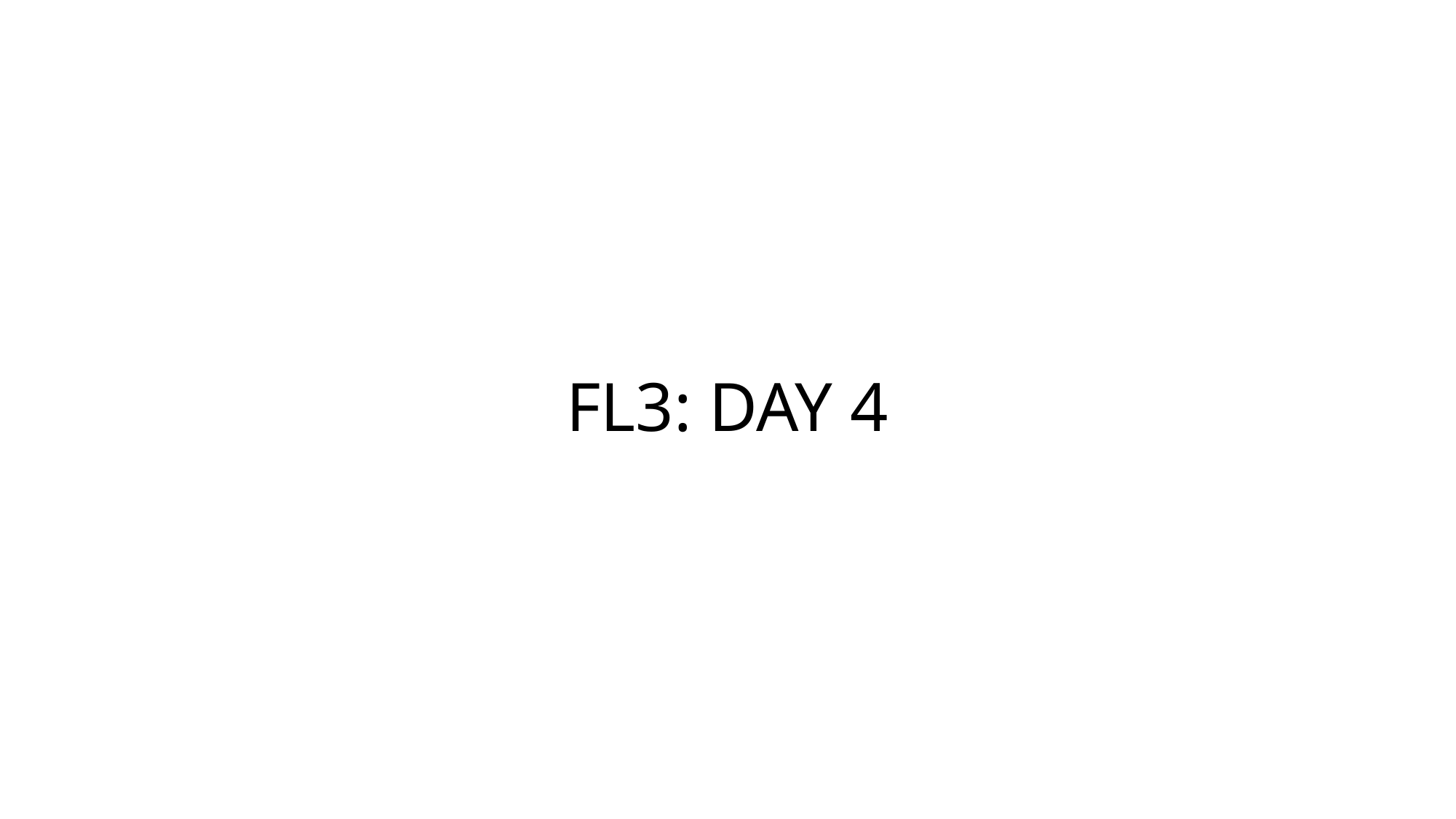

# FL3: DAY 4

## Slide 10
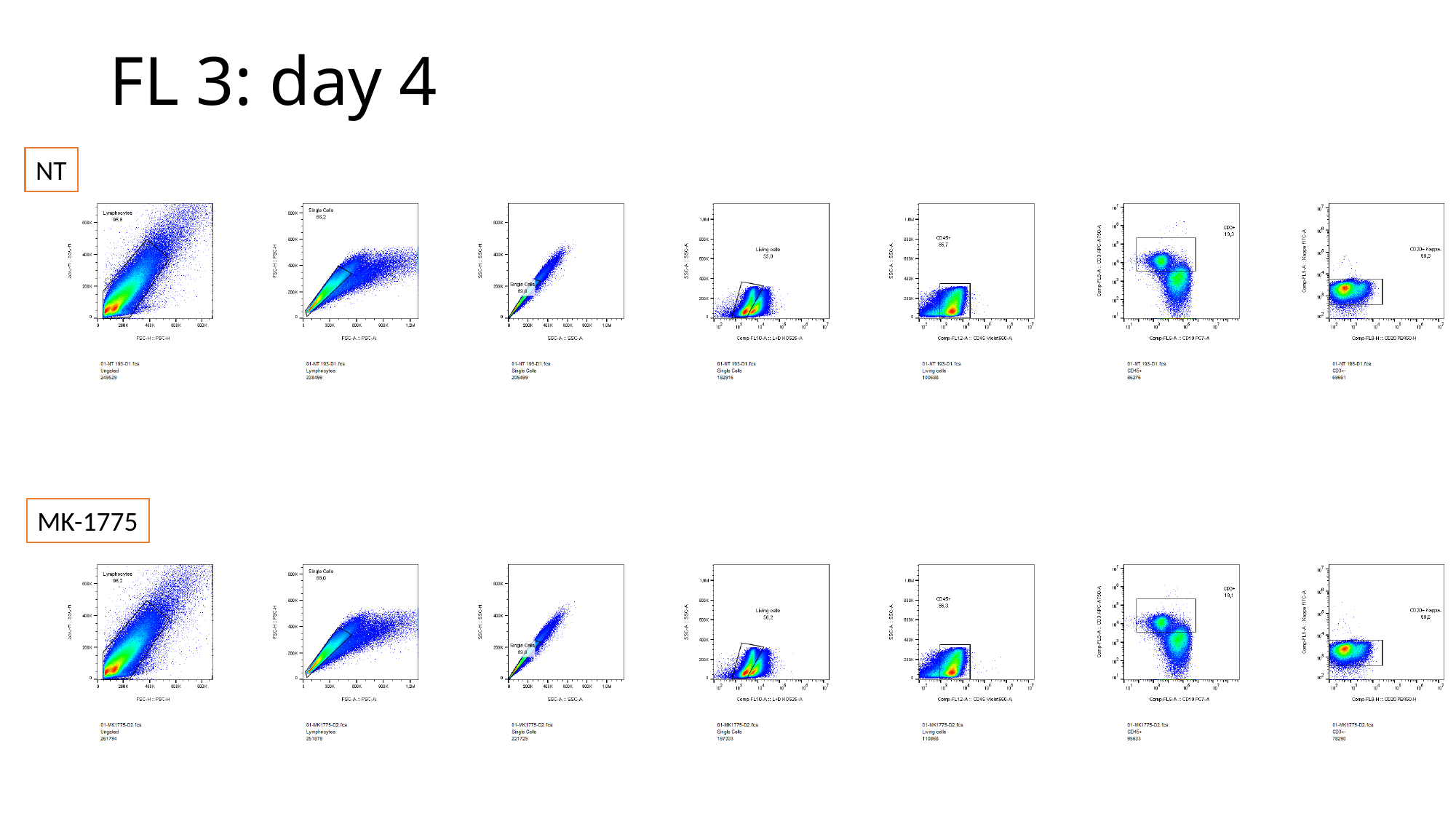

# FL 3: day 4
NT
MK-1775

## Slide 11
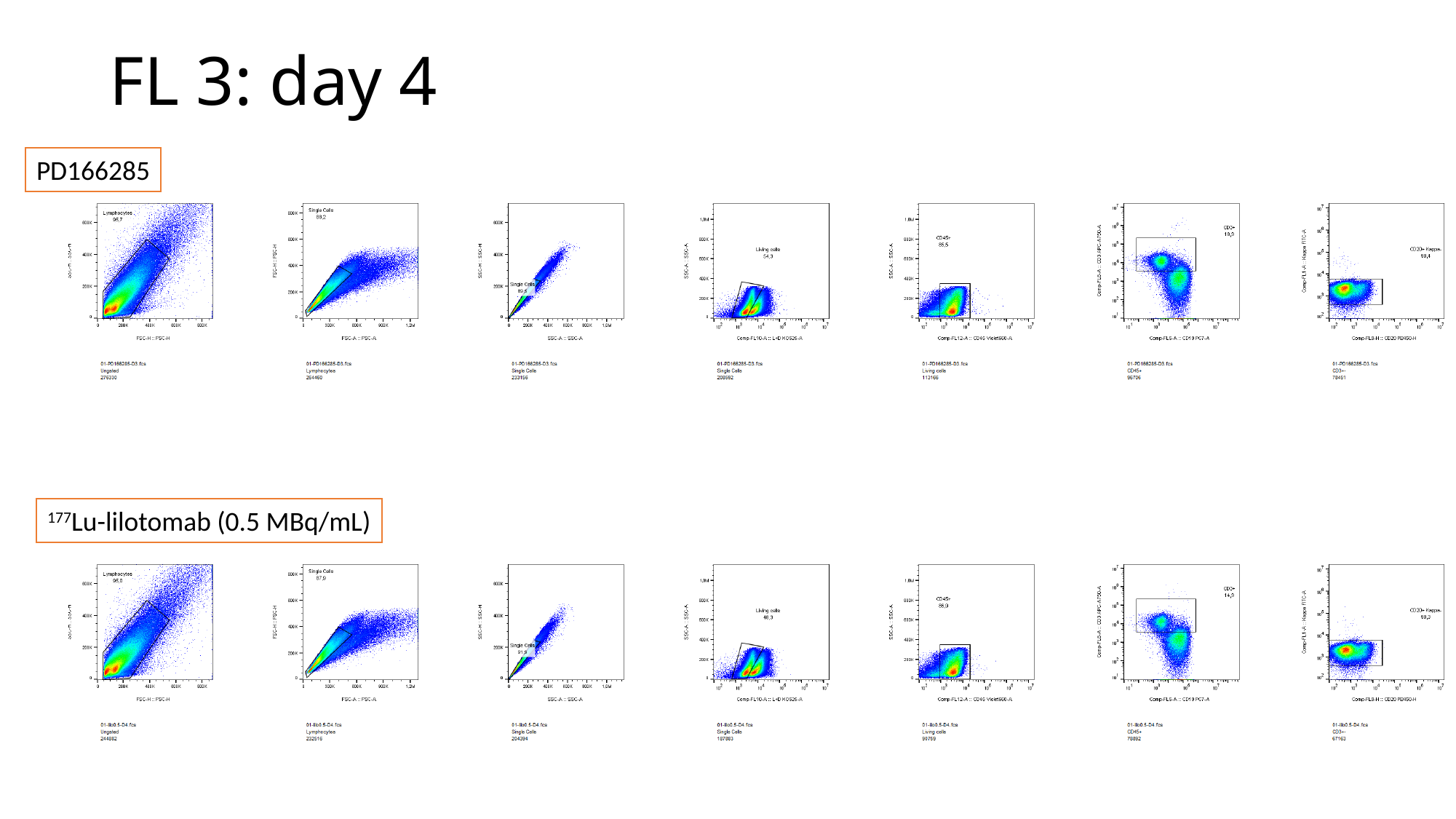

# FL 3: day 4
PD166285
177Lu-lilotomab (0.5 MBq/mL)

## Slide 12
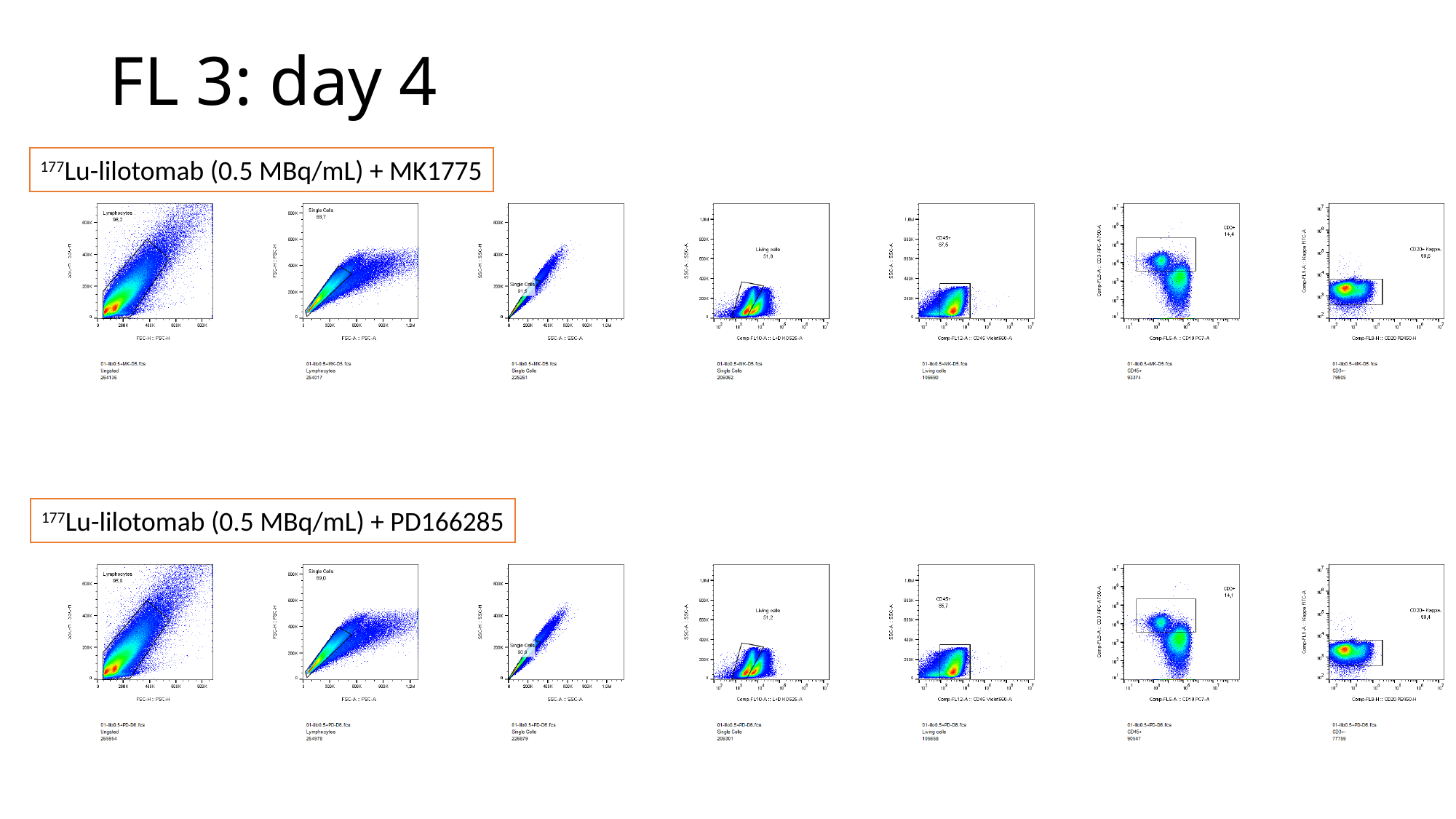

# FL 3: day 4
177Lu-lilotomab (0.5 MBq/mL) + MK1775
177Lu-lilotomab (0.5 MBq/mL) + PD166285

## Slide 13
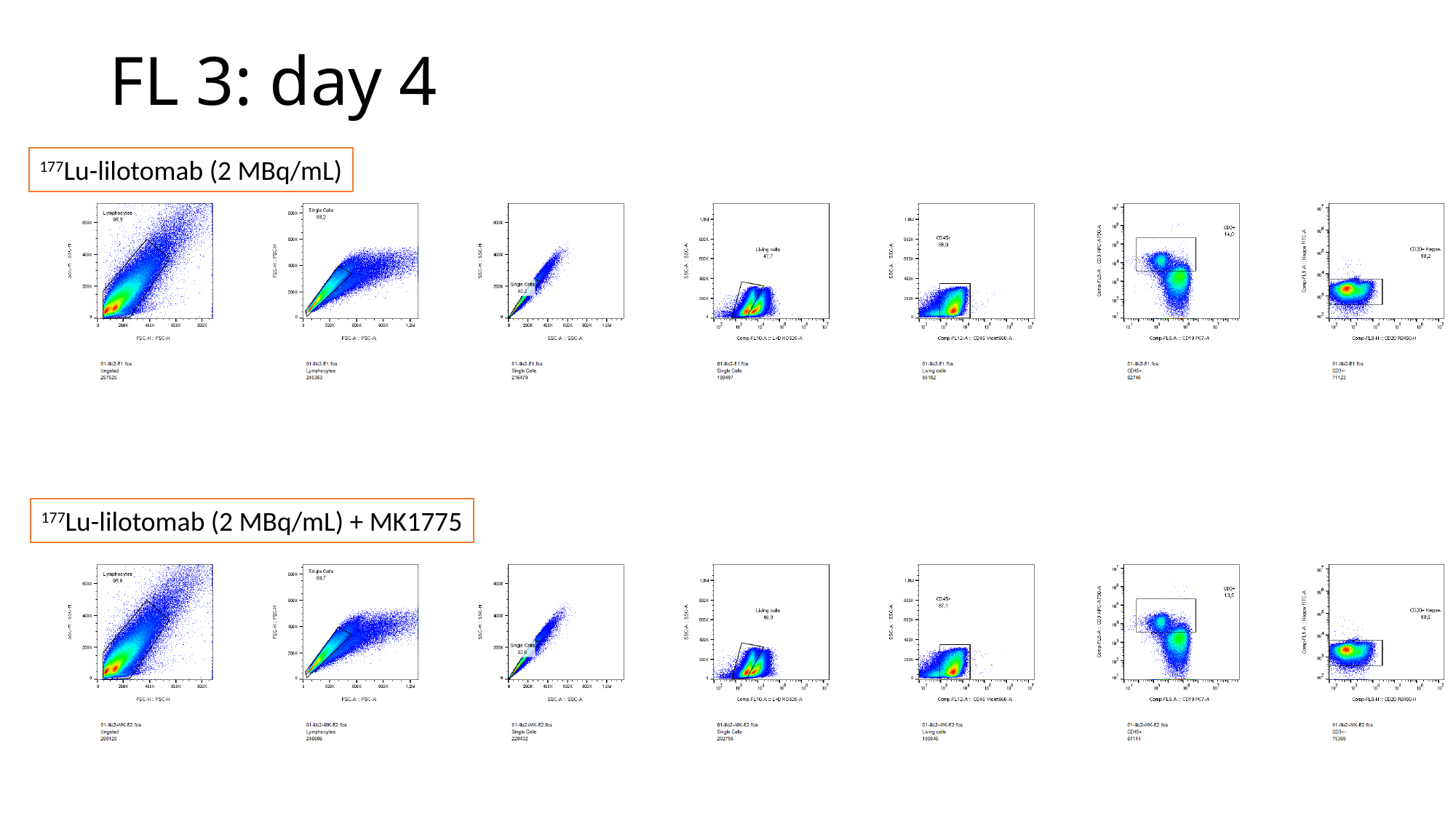

# FL 3: day 4
177Lu-lilotomab (2 MBq/mL)
177Lu-lilotomab (2 MBq/mL) + MK1775

## Slide 14
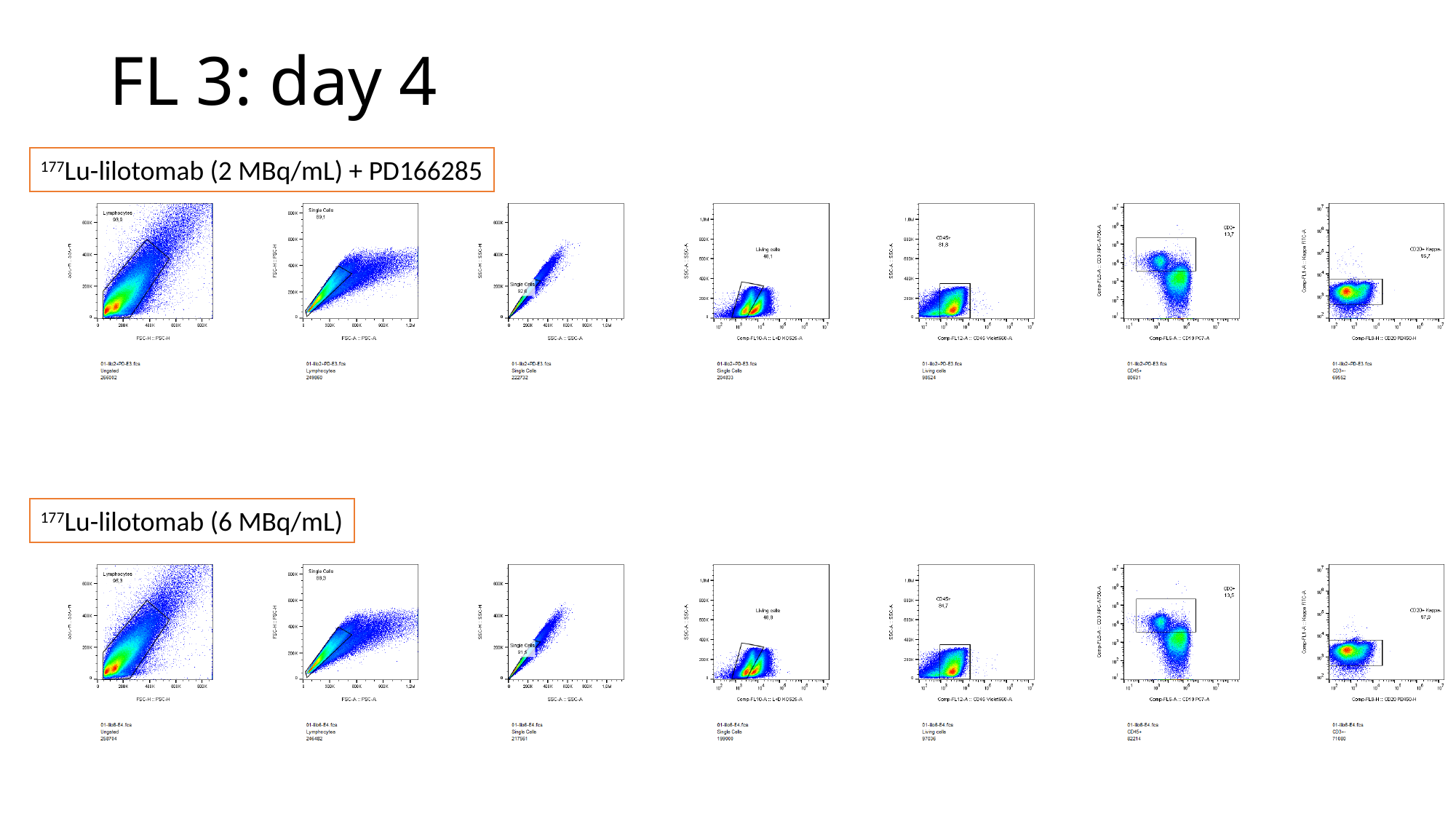

# FL 3: day 4
177Lu-lilotomab (2 MBq/mL) + PD166285
177Lu-lilotomab (6 MBq/mL)

## Slide 15
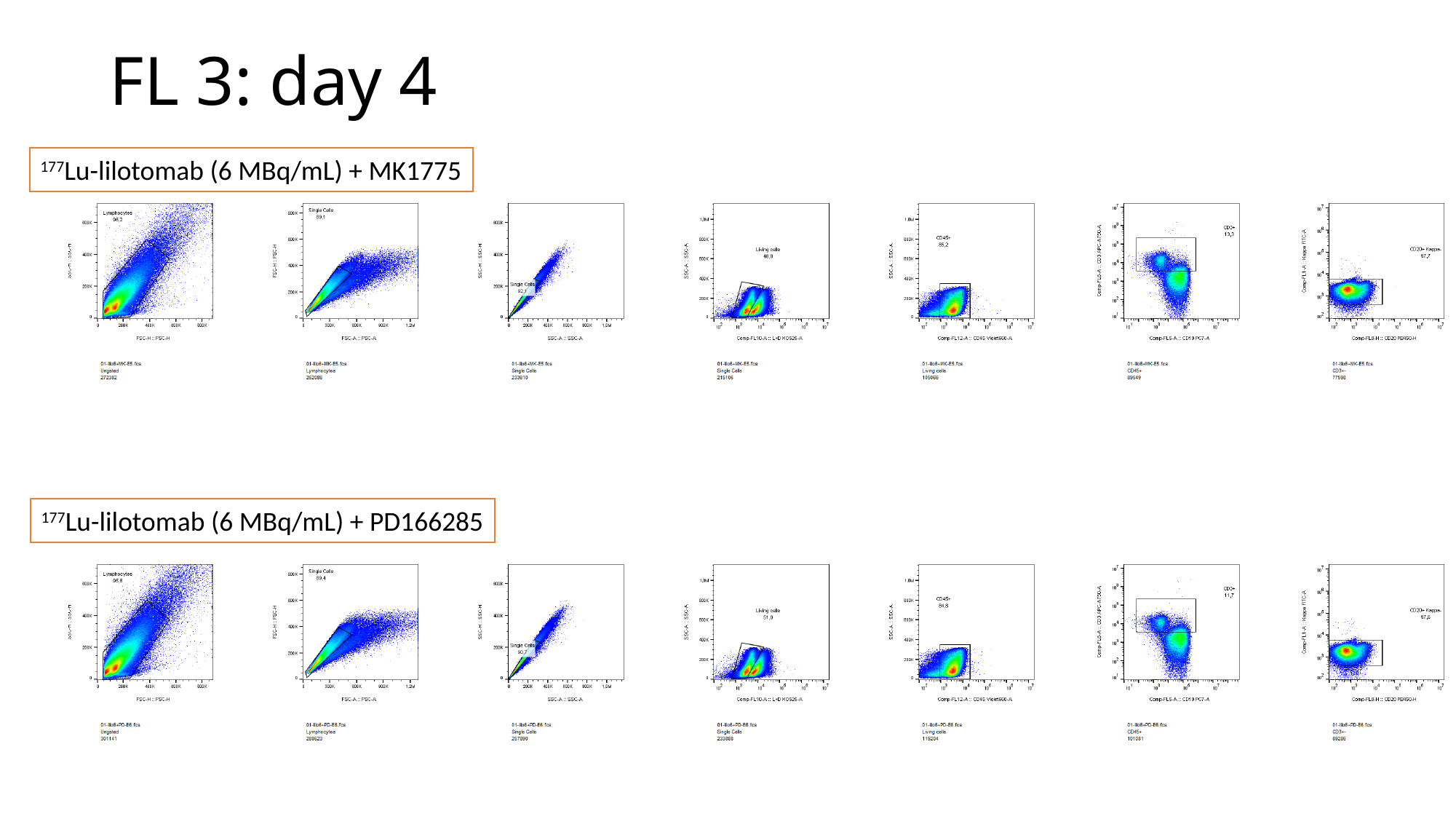

# FL 3: day 4
177Lu-lilotomab (6 MBq/mL) + MK1775
177Lu-lilotomab (6 MBq/mL) + PD166285

## Slide 16
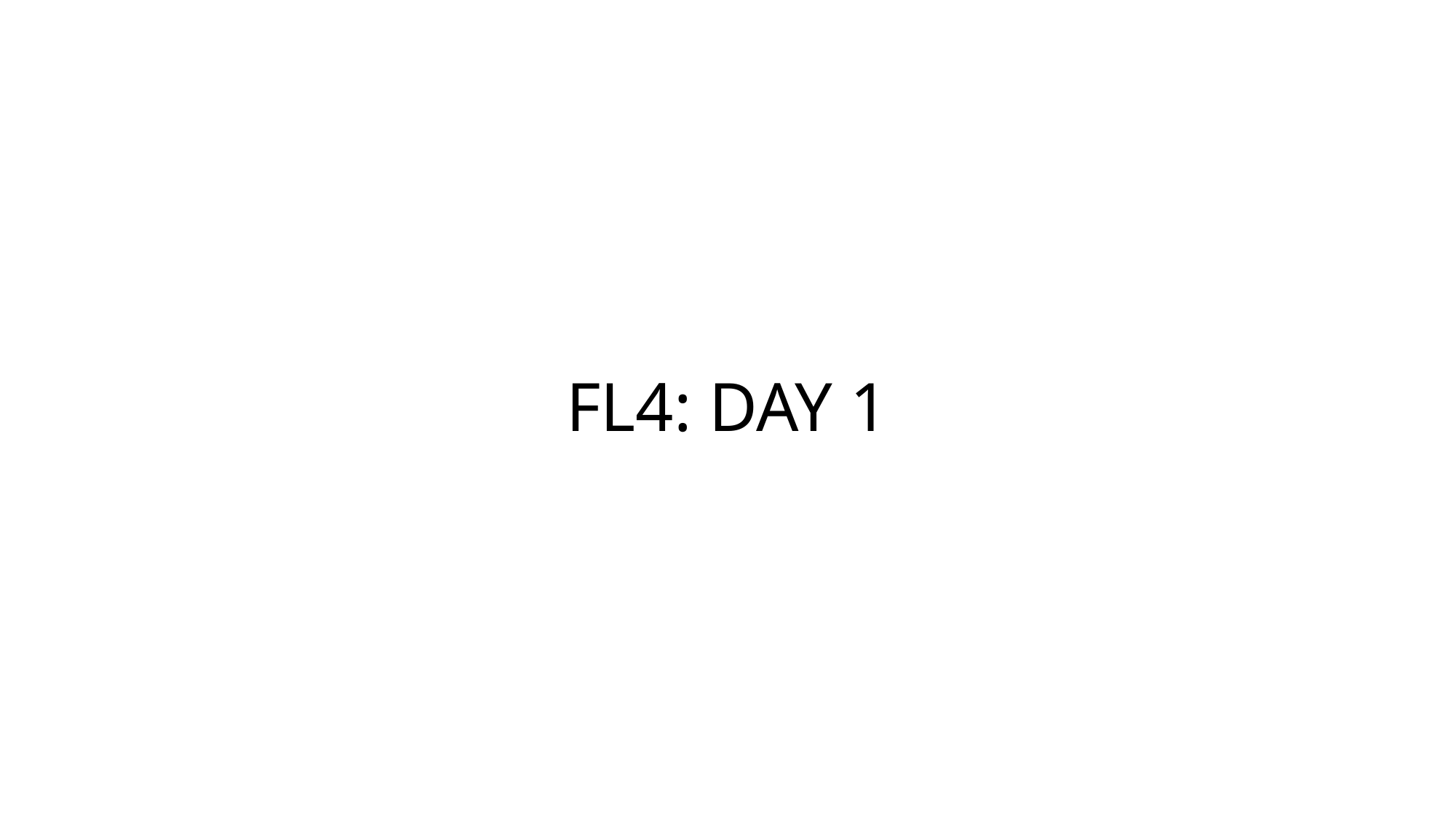

# FL4: DAY 1

## Slide 17
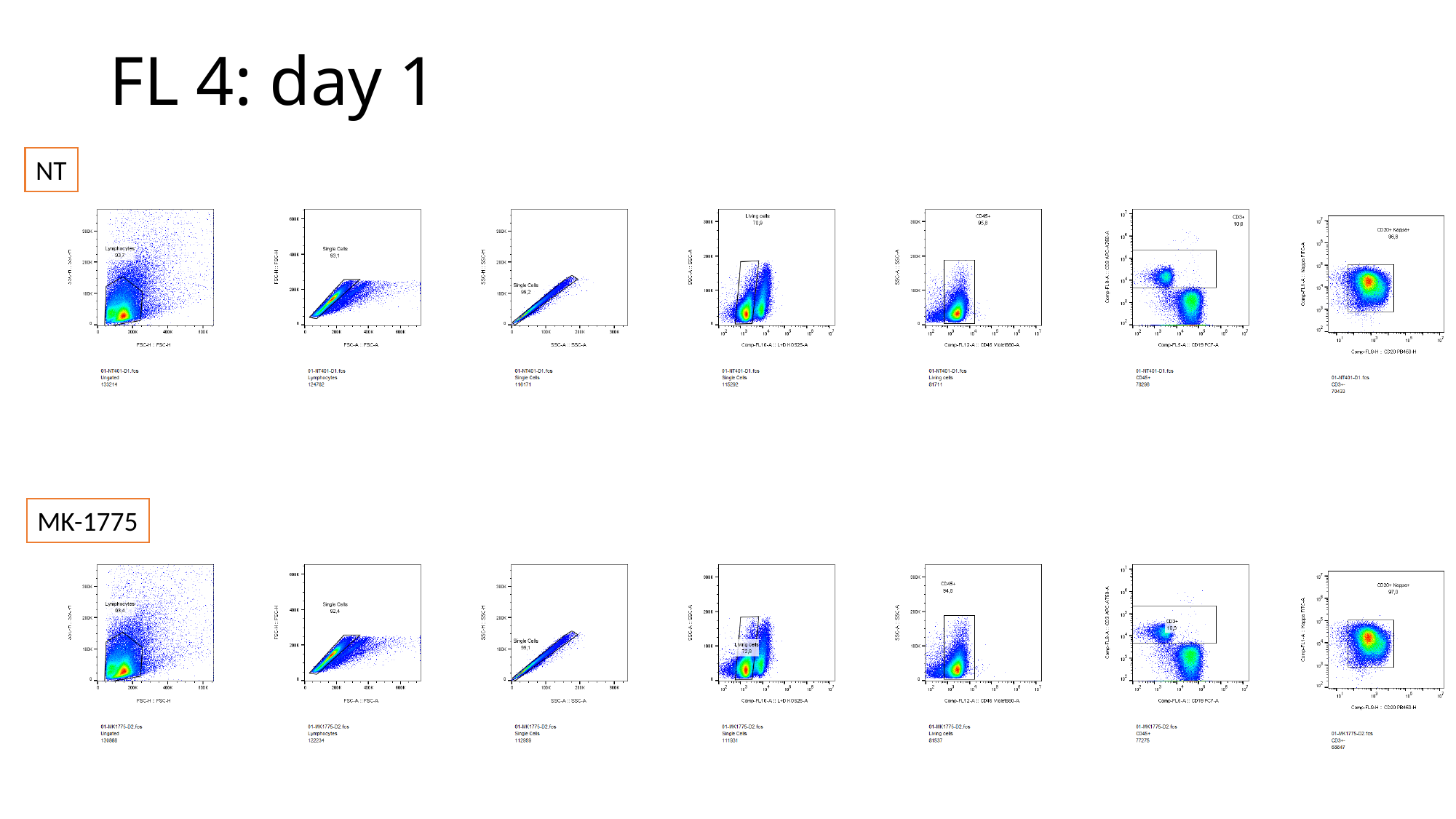

# FL 4: day 1
NT
MK-1775

## Slide 18
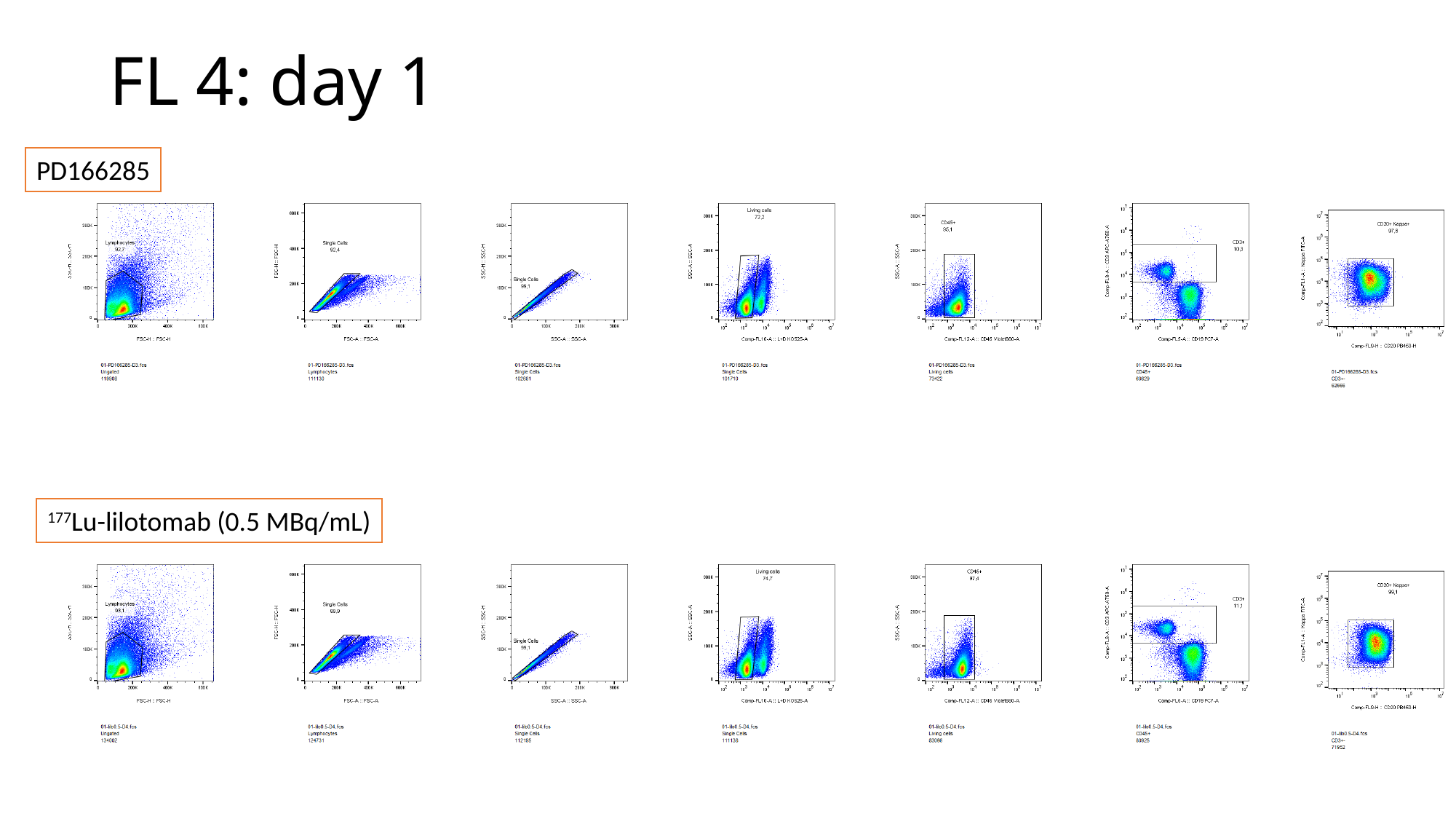

# FL 4: day 1
PD166285
177Lu-lilotomab (0.5 MBq/mL)

## Slide 19
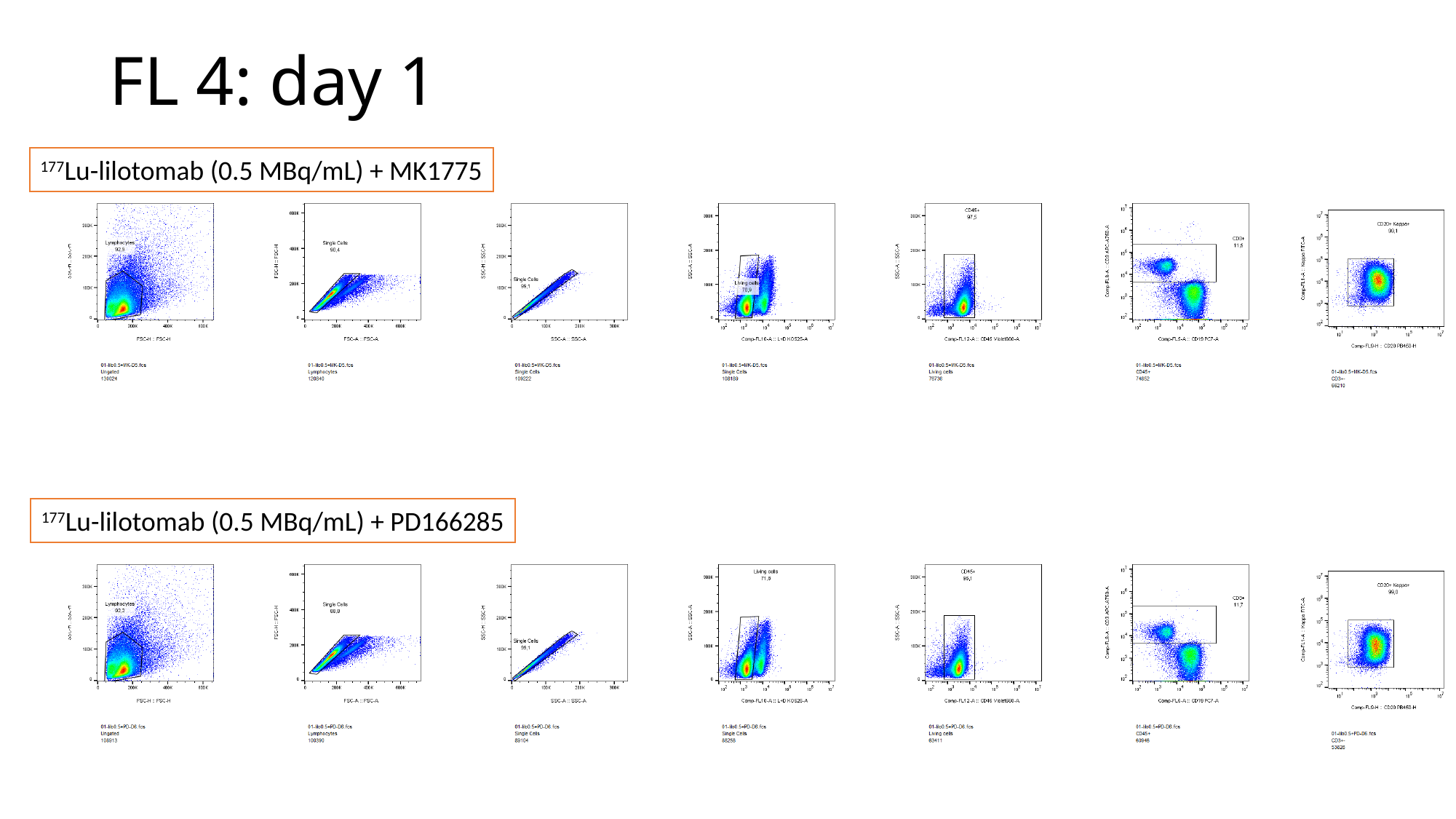

# FL 4: day 1
177Lu-lilotomab (0.5 MBq/mL) + MK1775
177Lu-lilotomab (0.5 MBq/mL) + PD166285

## Slide 20
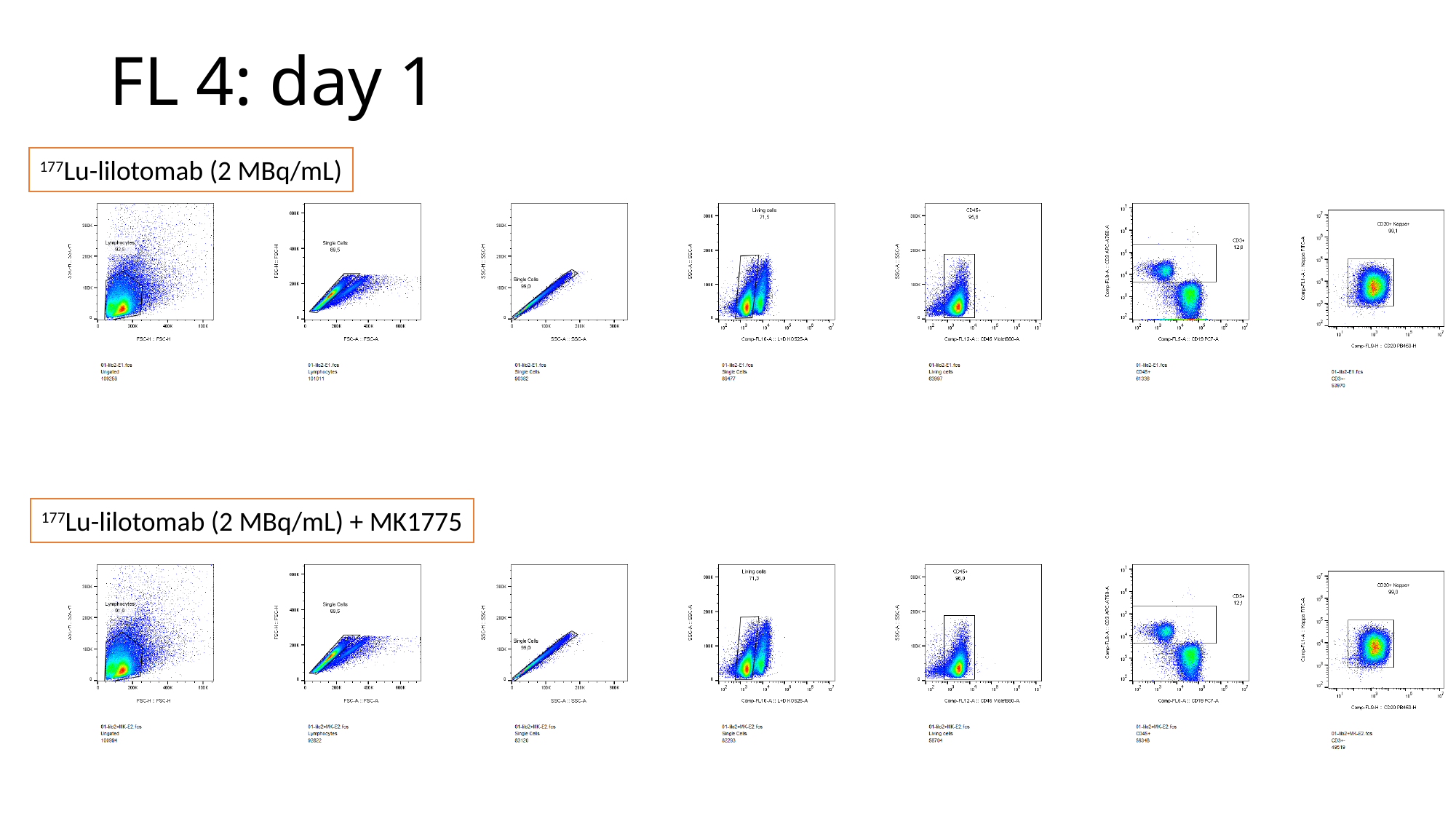

# FL 4: day 1
177Lu-lilotomab (2 MBq/mL)
177Lu-lilotomab (2 MBq/mL) + MK1775

## Slide 21
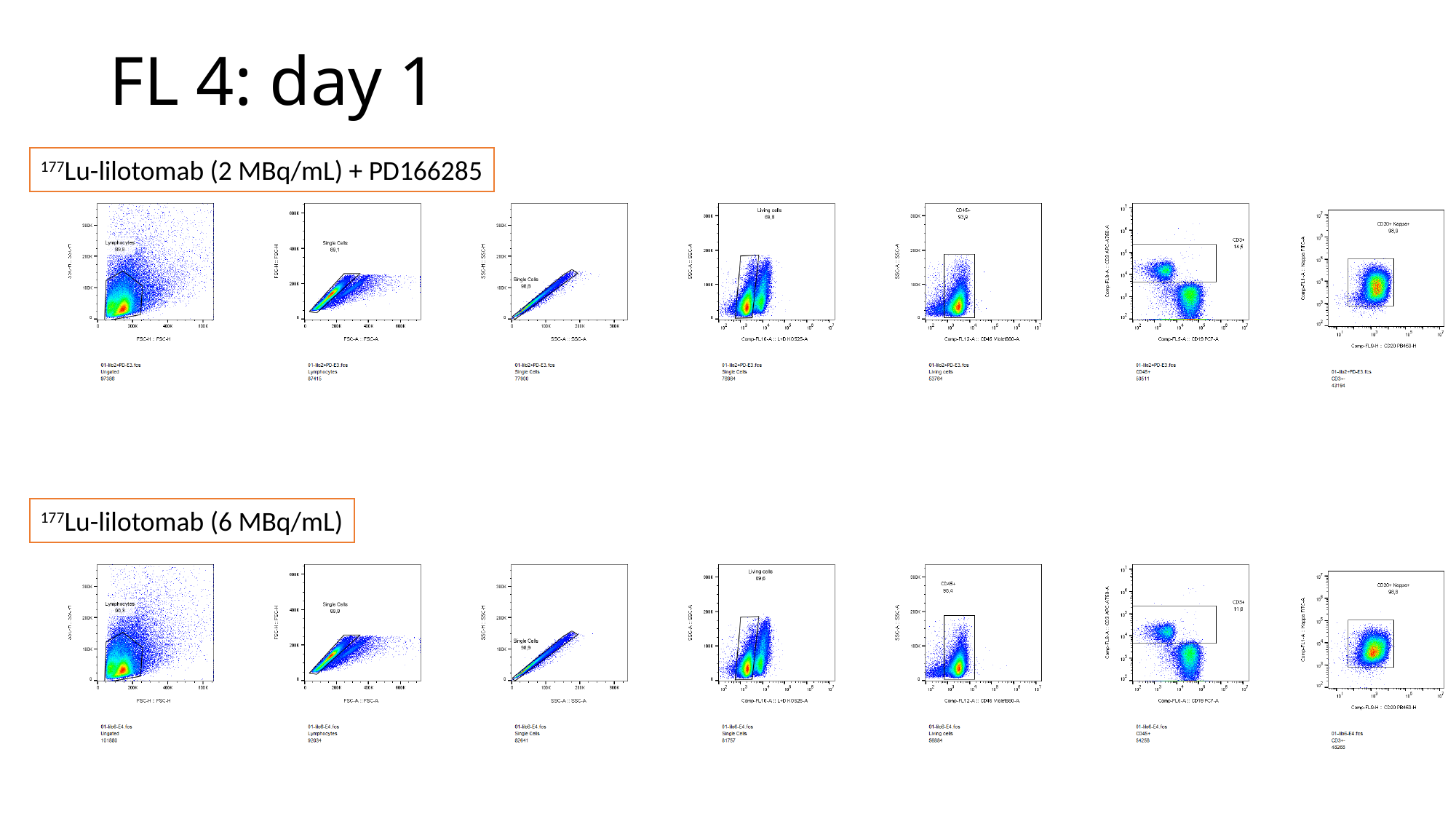

# FL 4: day 1
177Lu-lilotomab (2 MBq/mL) + PD166285
177Lu-lilotomab (6 MBq/mL)

## Slide 22
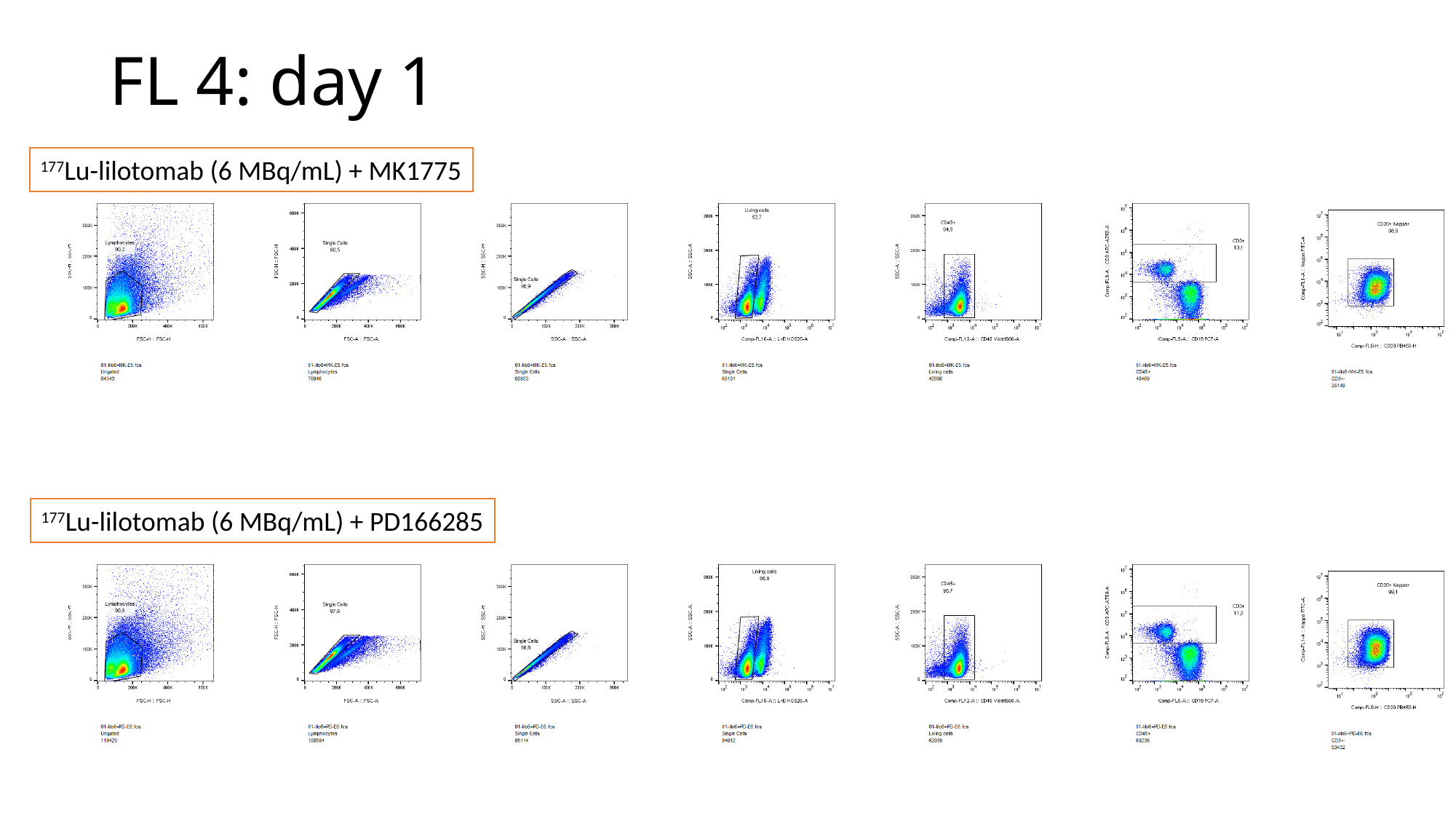

# FL 4: day 1
177Lu-lilotomab (6 MBq/mL) + MK1775
177Lu-lilotomab (6 MBq/mL) + PD166285

## Slide 23
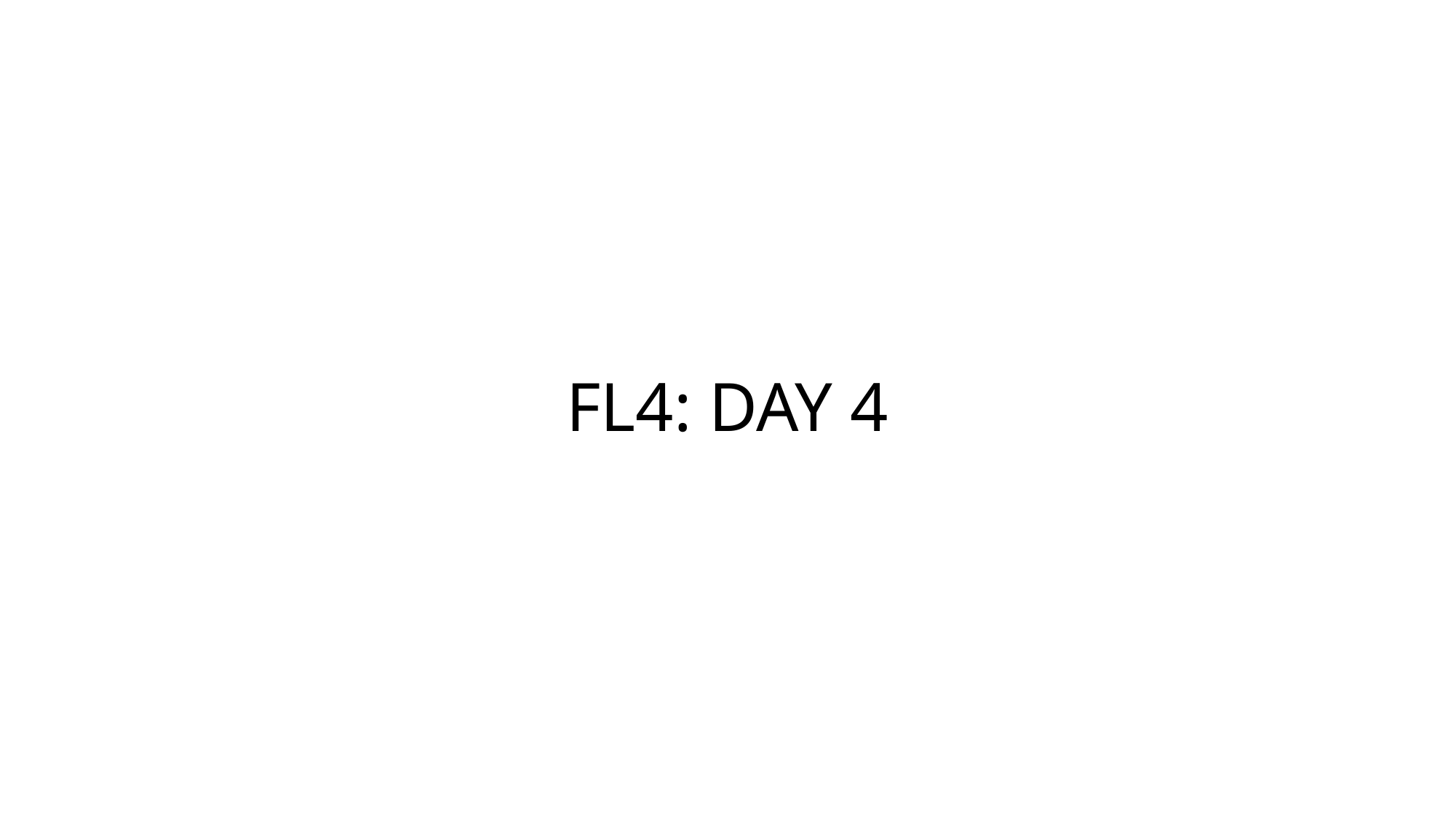

# FL4: DAY 4

## Slide 24
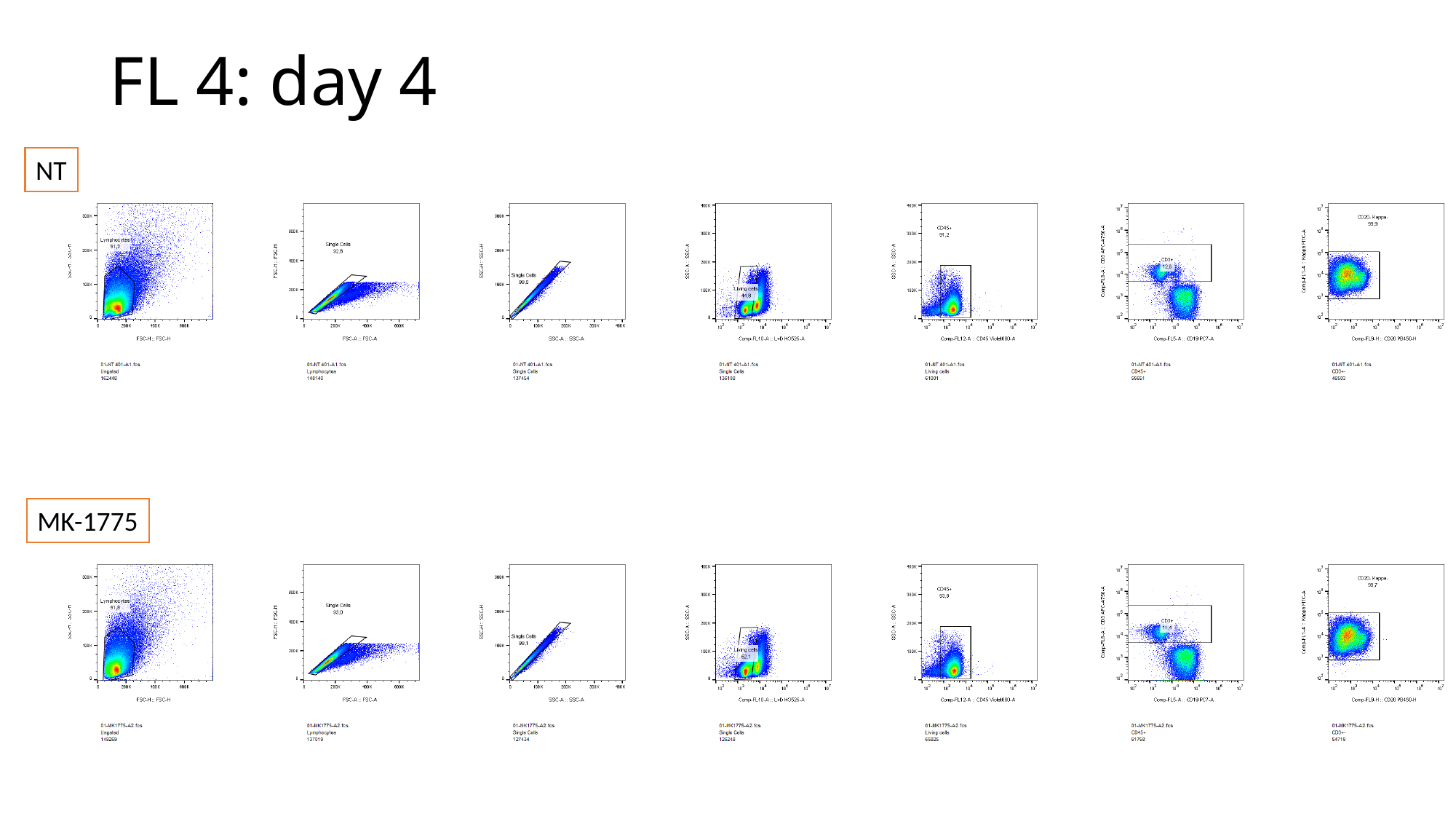

# FL 4: day 4
NT
MK-1775

## Slide 25
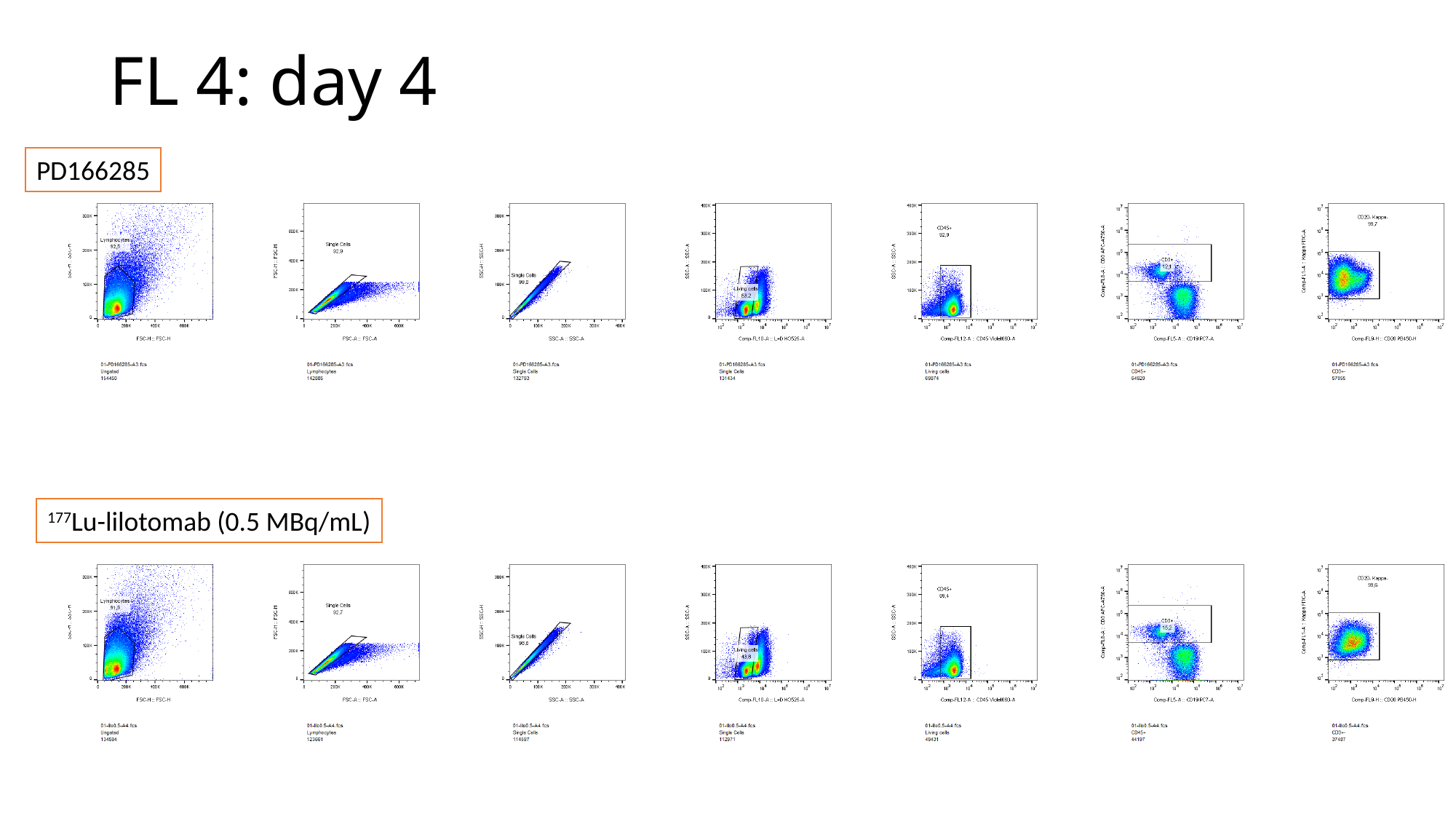

# FL 4: day 4
PD166285
177Lu-lilotomab (0.5 MBq/mL)

## Slide 26
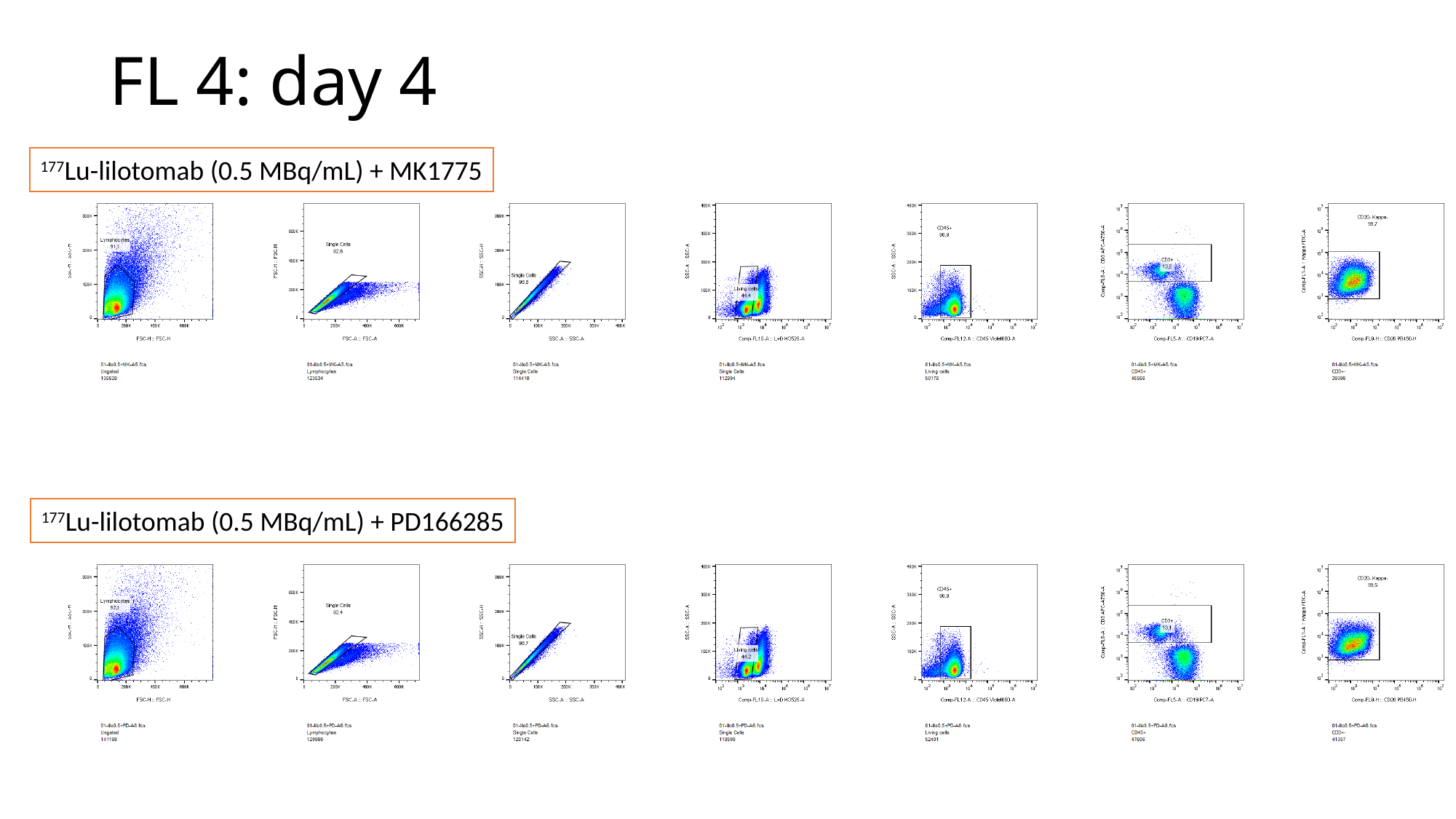

# FL 4: day 4
177Lu-lilotomab (0.5 MBq/mL) + MK1775
177Lu-lilotomab (0.5 MBq/mL) + PD166285

## Slide 27
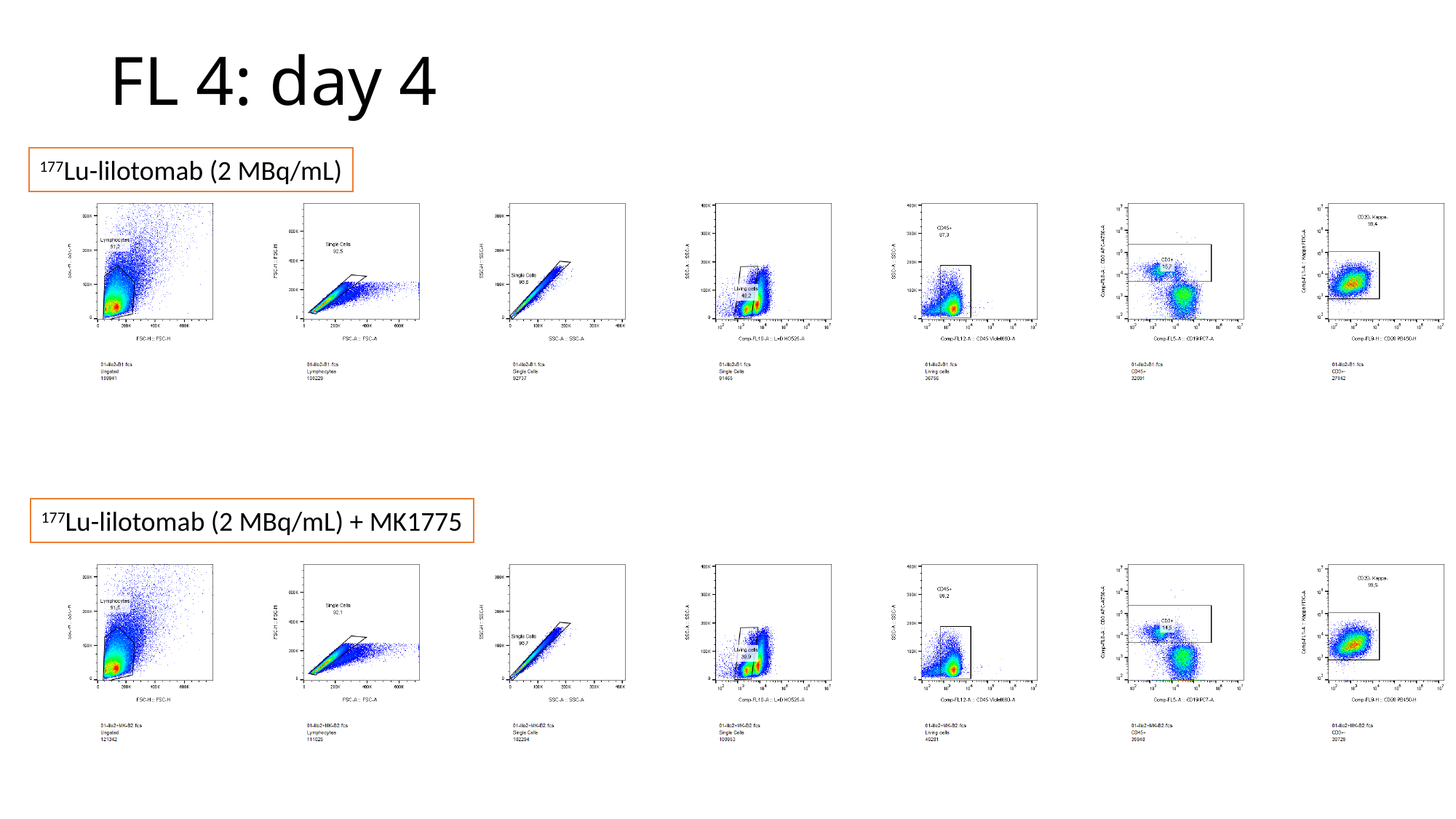

# FL 4: day 4
177Lu-lilotomab (2 MBq/mL)
177Lu-lilotomab (2 MBq/mL) + MK1775

## Slide 28
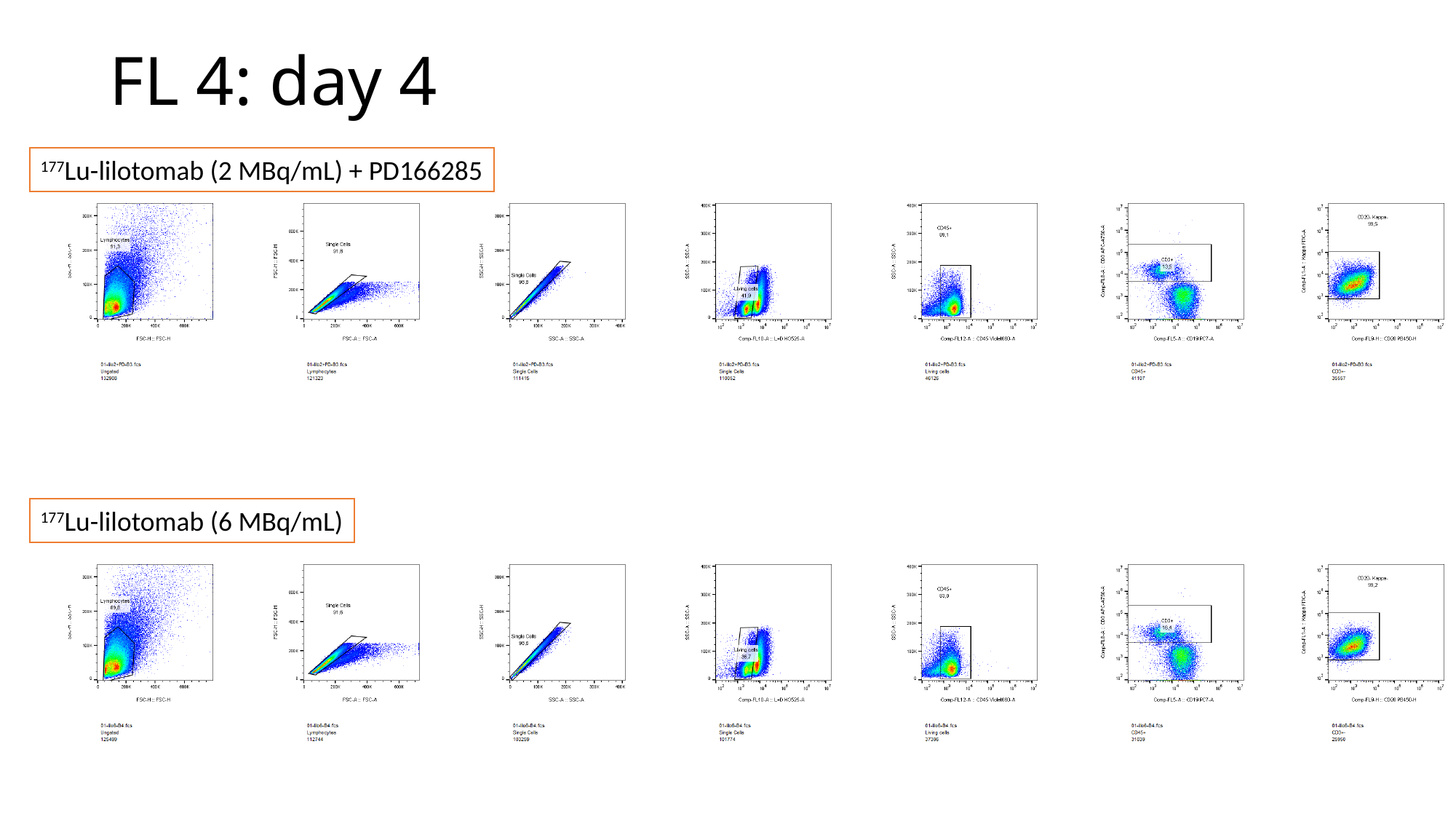

# FL 4: day 4
177Lu-lilotomab (2 MBq/mL) + PD166285
177Lu-lilotomab (6 MBq/mL)

## Slide 29
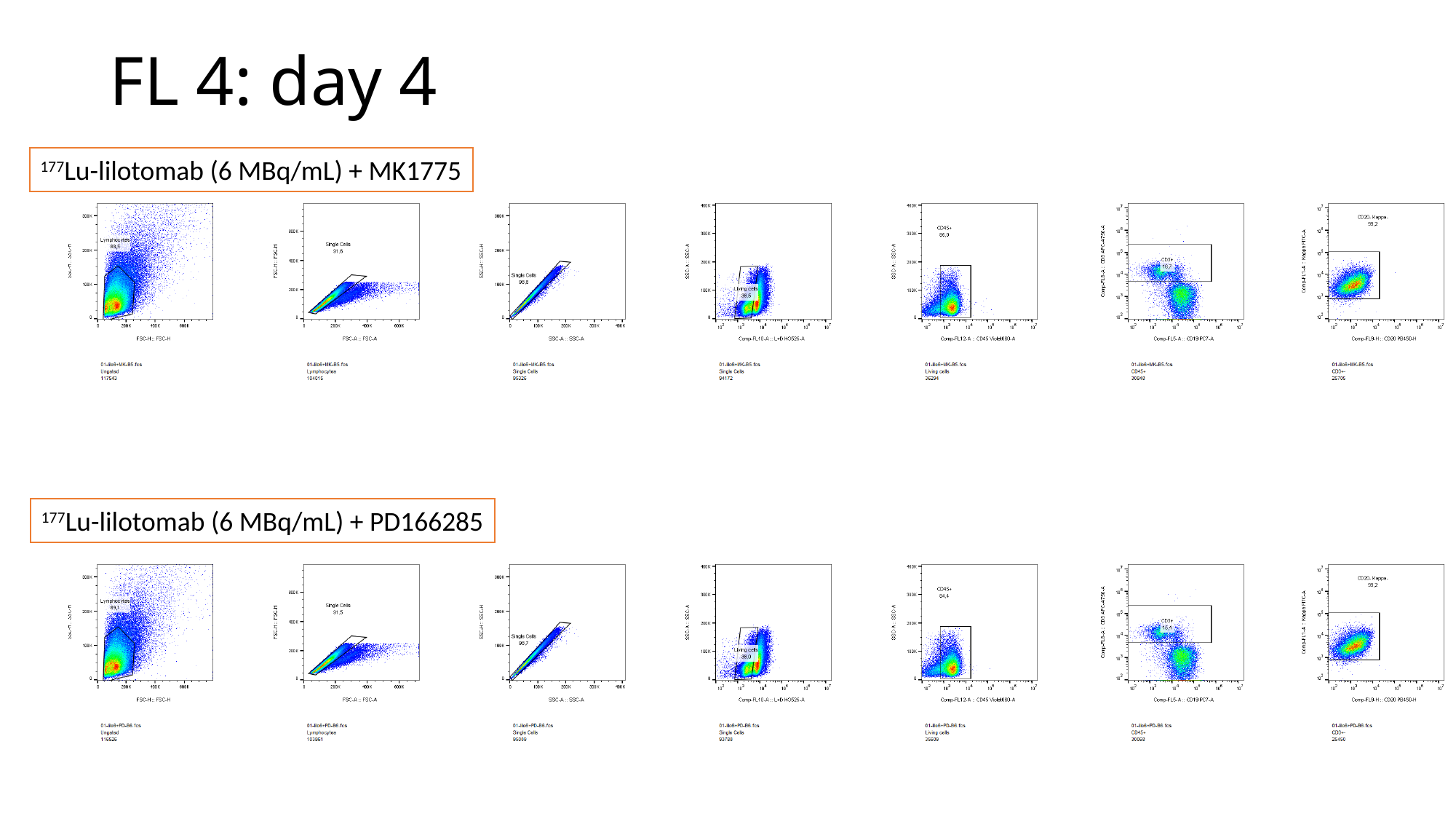

# FL 4: day 4
177Lu-lilotomab (6 MBq/mL) + MK1775
177Lu-lilotomab (6 MBq/mL) + PD166285

## Slide 30
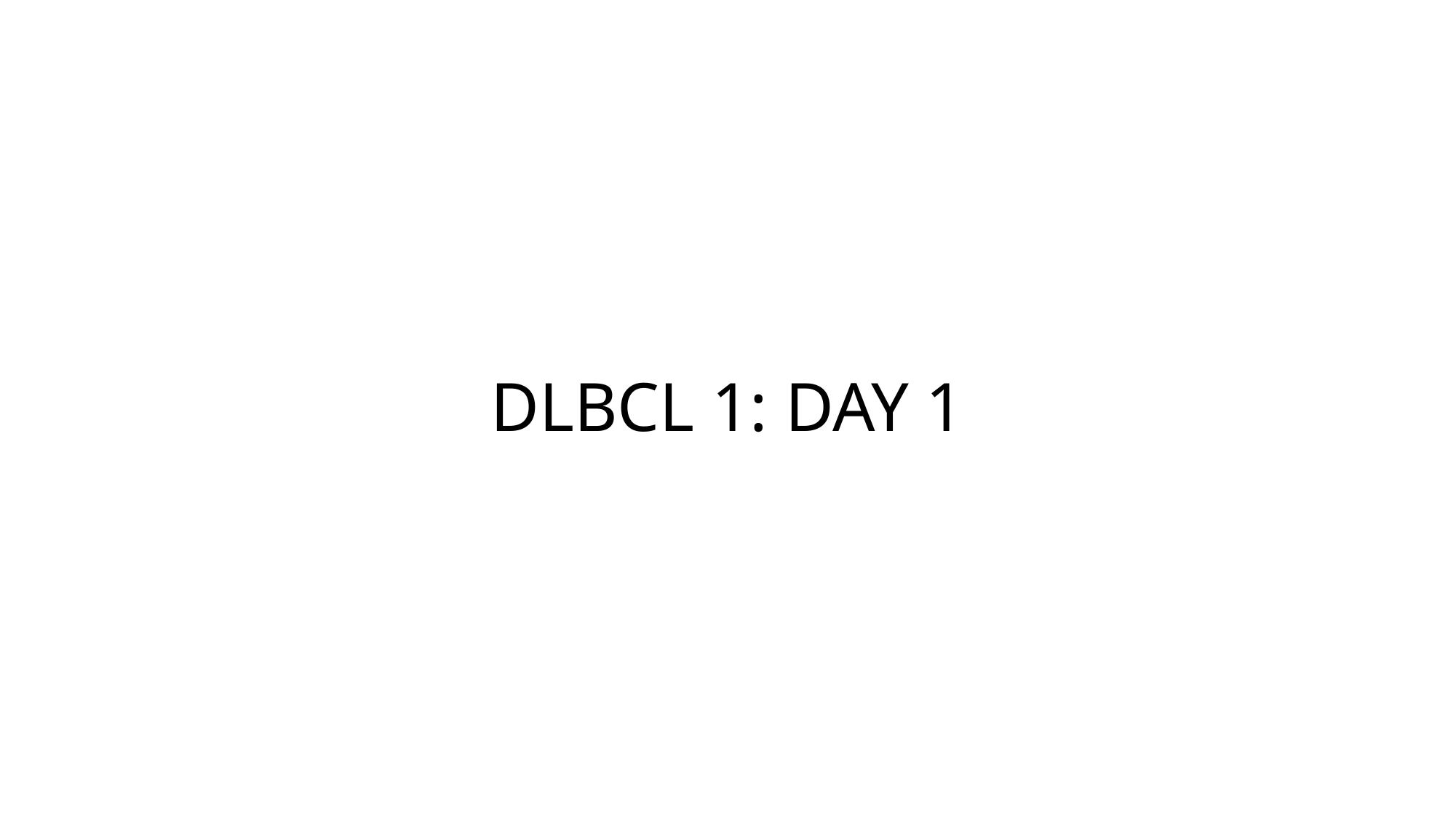

# DLBCL 1: DAY 1

## Slide 31
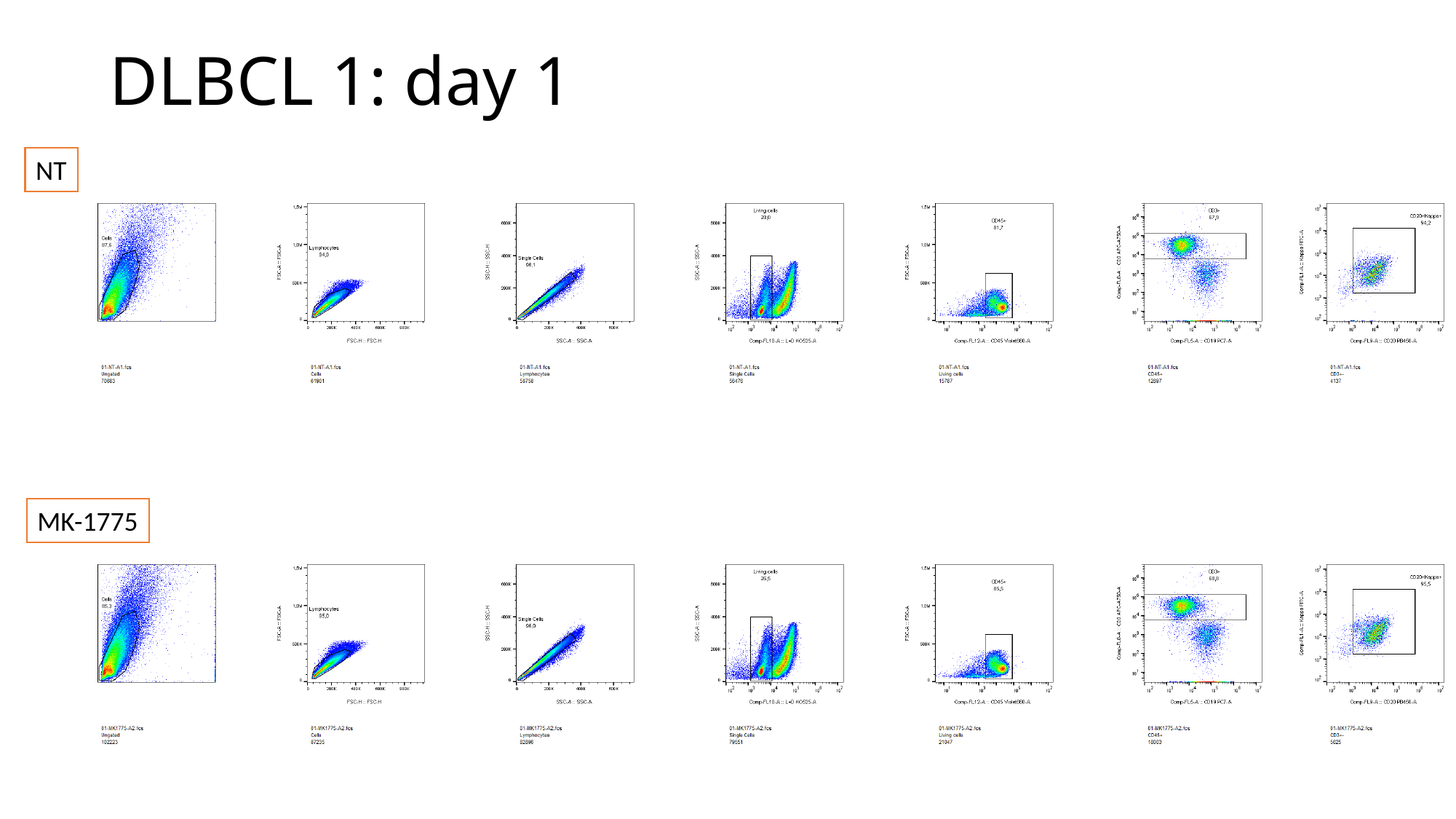

# DLBCL 1: day 1
NT
MK-1775

## Slide 32
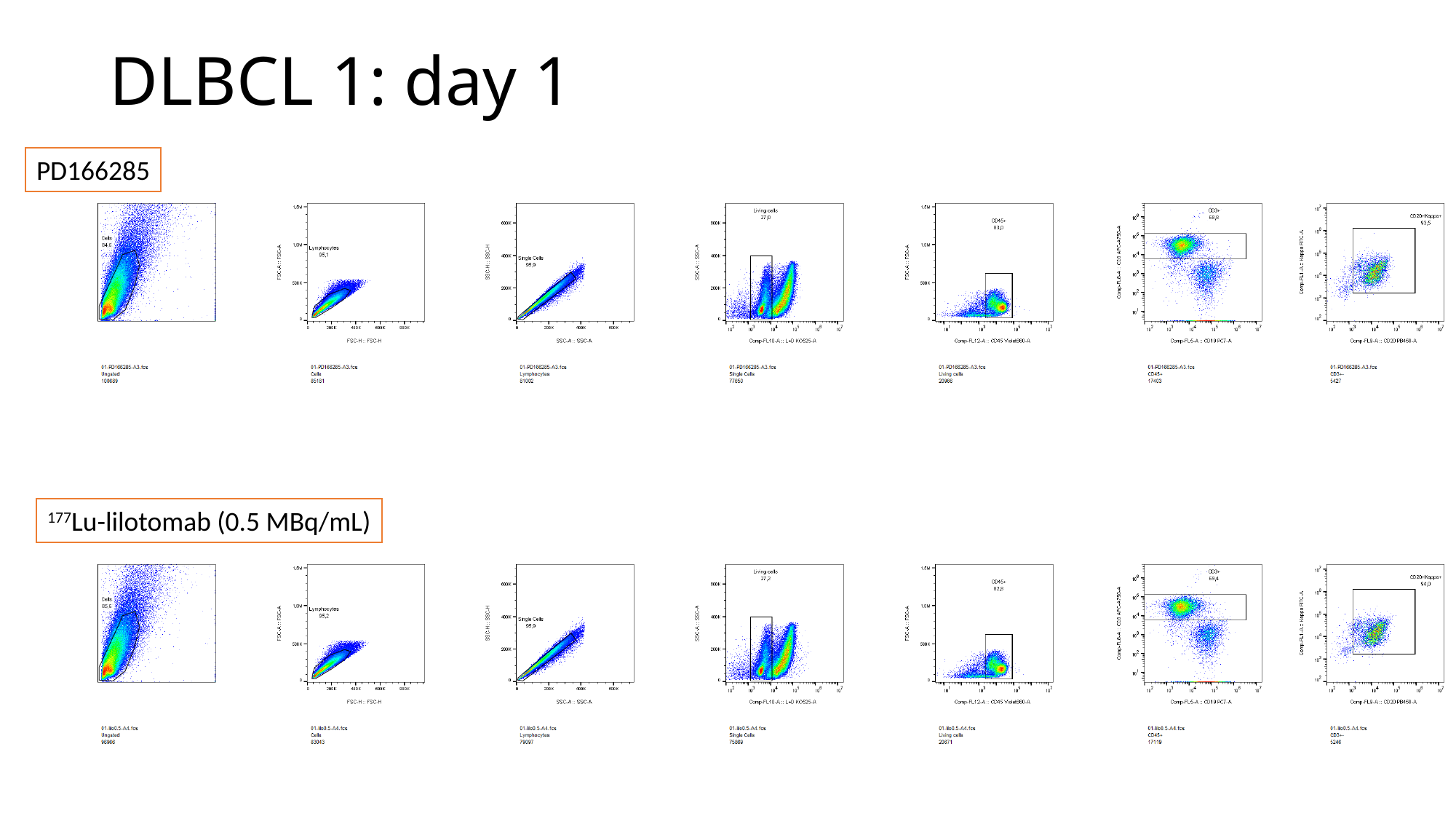

# DLBCL 1: day 1
PD166285
177Lu-lilotomab (0.5 MBq/mL)

## Slide 33
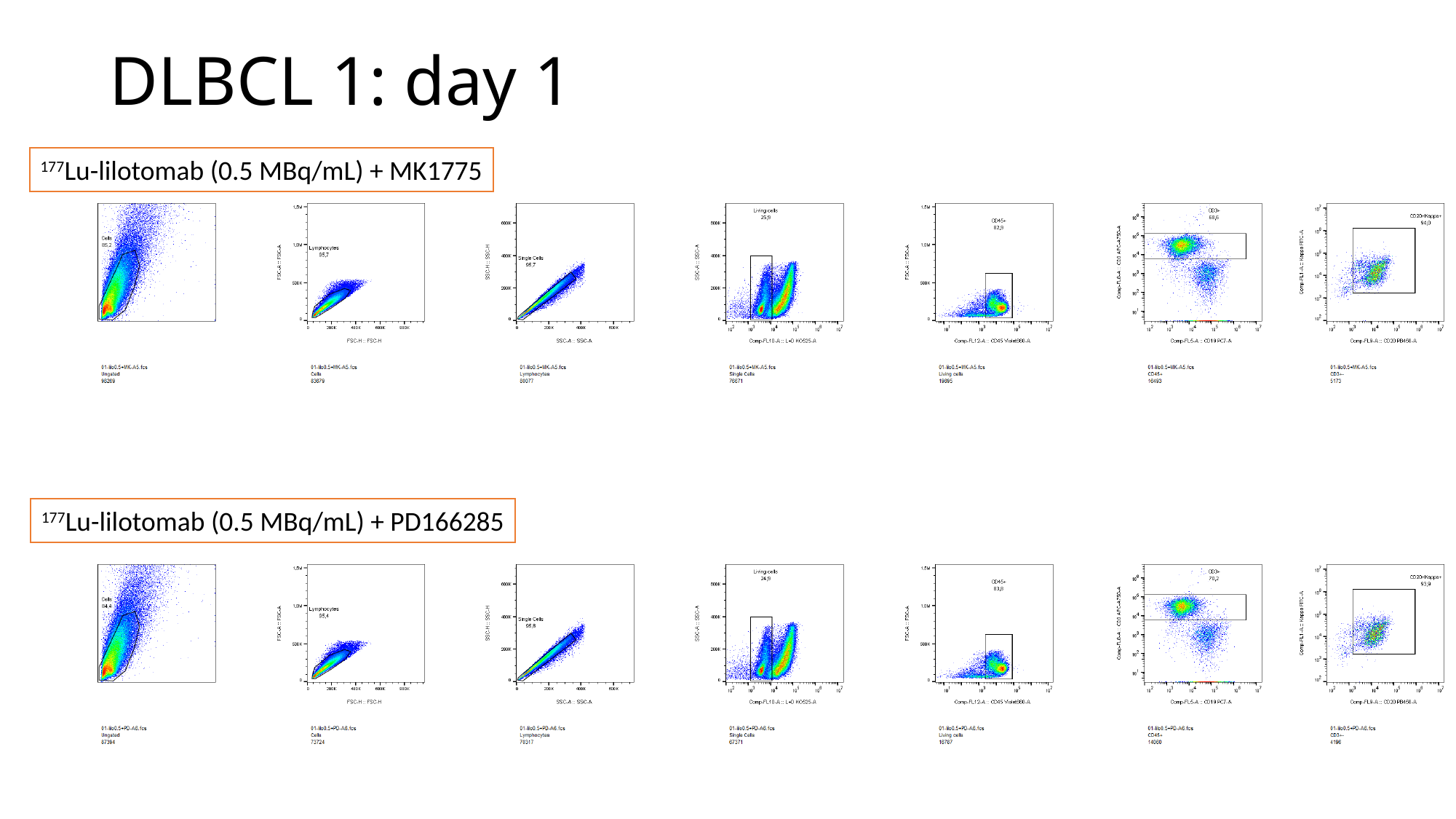

# DLBCL 1: day 1
177Lu-lilotomab (0.5 MBq/mL) + MK1775
177Lu-lilotomab (0.5 MBq/mL) + PD166285

## Slide 34
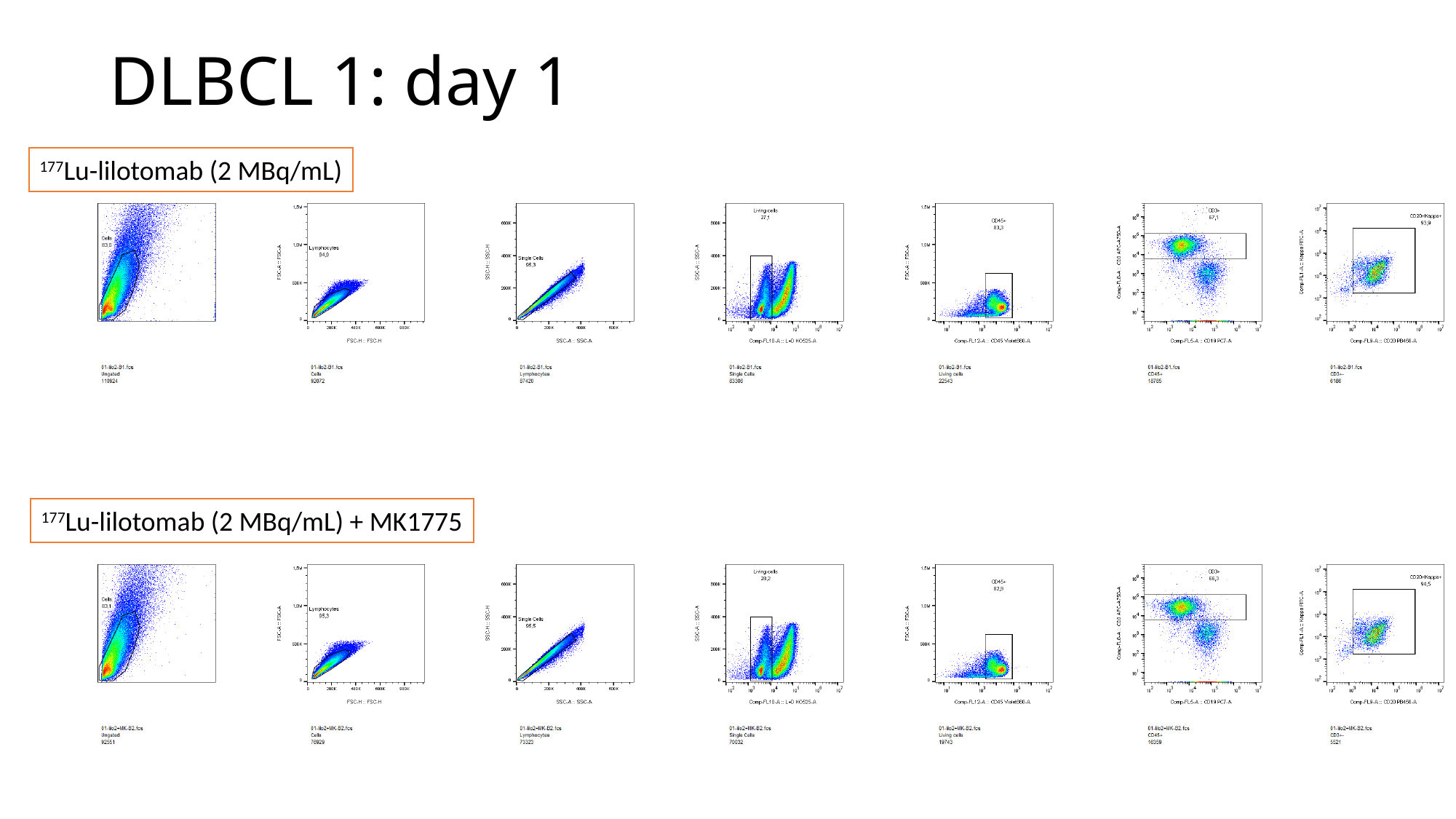

# DLBCL 1: day 1
177Lu-lilotomab (2 MBq/mL)
177Lu-lilotomab (2 MBq/mL) + MK1775

## Slide 35
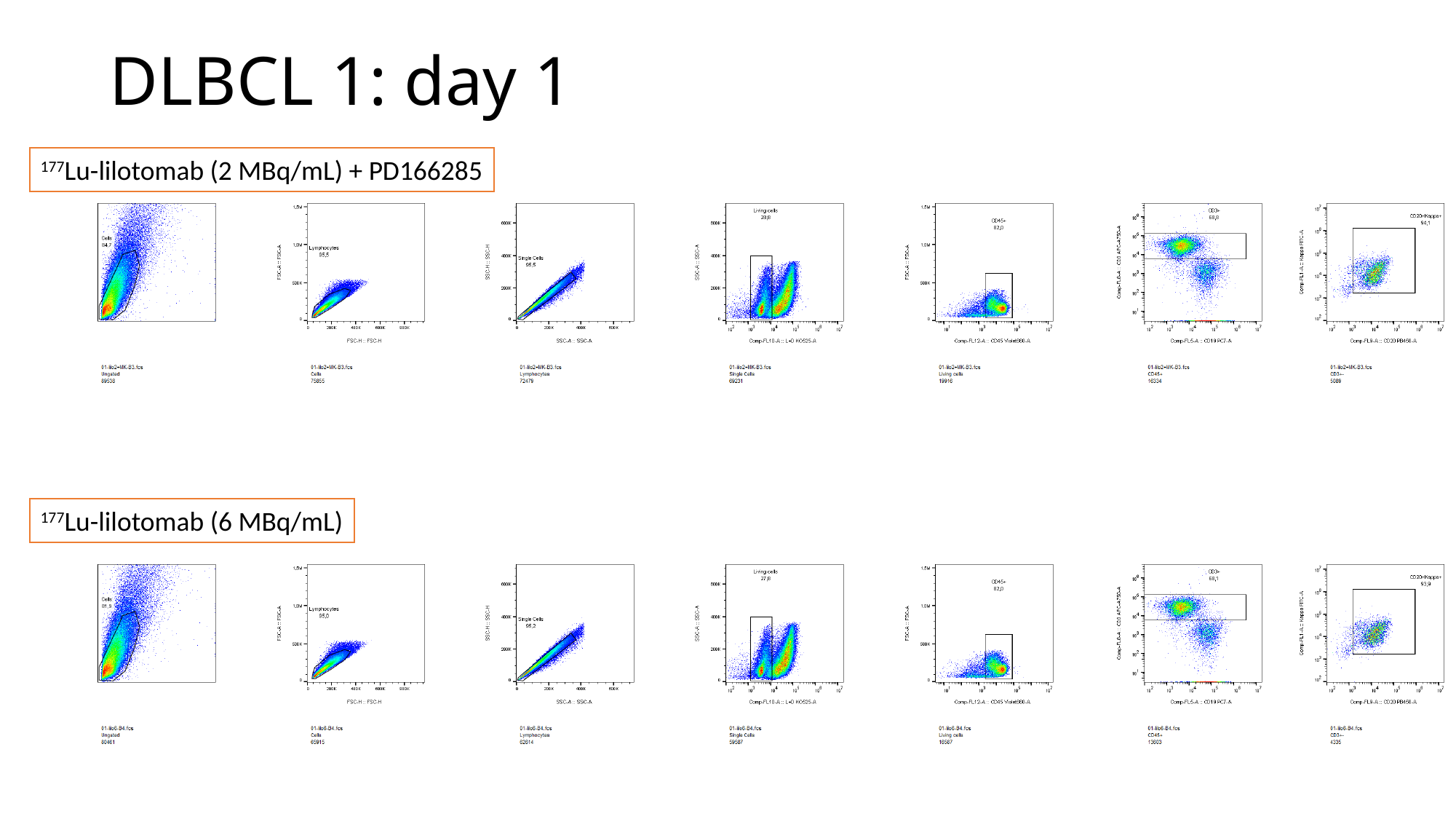

# DLBCL 1: day 1
177Lu-lilotomab (2 MBq/mL) + PD166285
177Lu-lilotomab (6 MBq/mL)

## Slide 36
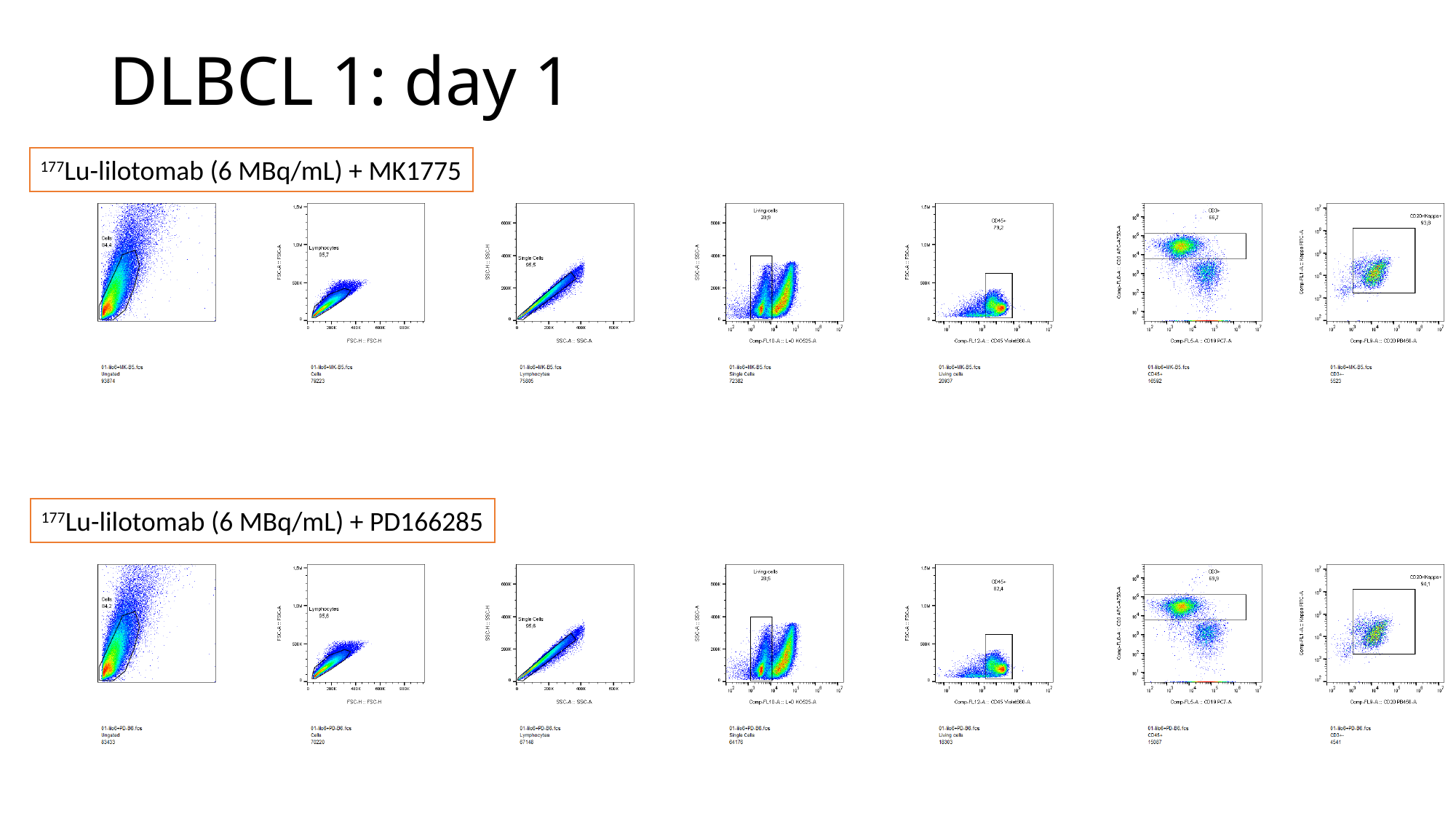

# DLBCL 1: day 1
177Lu-lilotomab (6 MBq/mL) + MK1775
177Lu-lilotomab (6 MBq/mL) + PD166285

## Slide 37
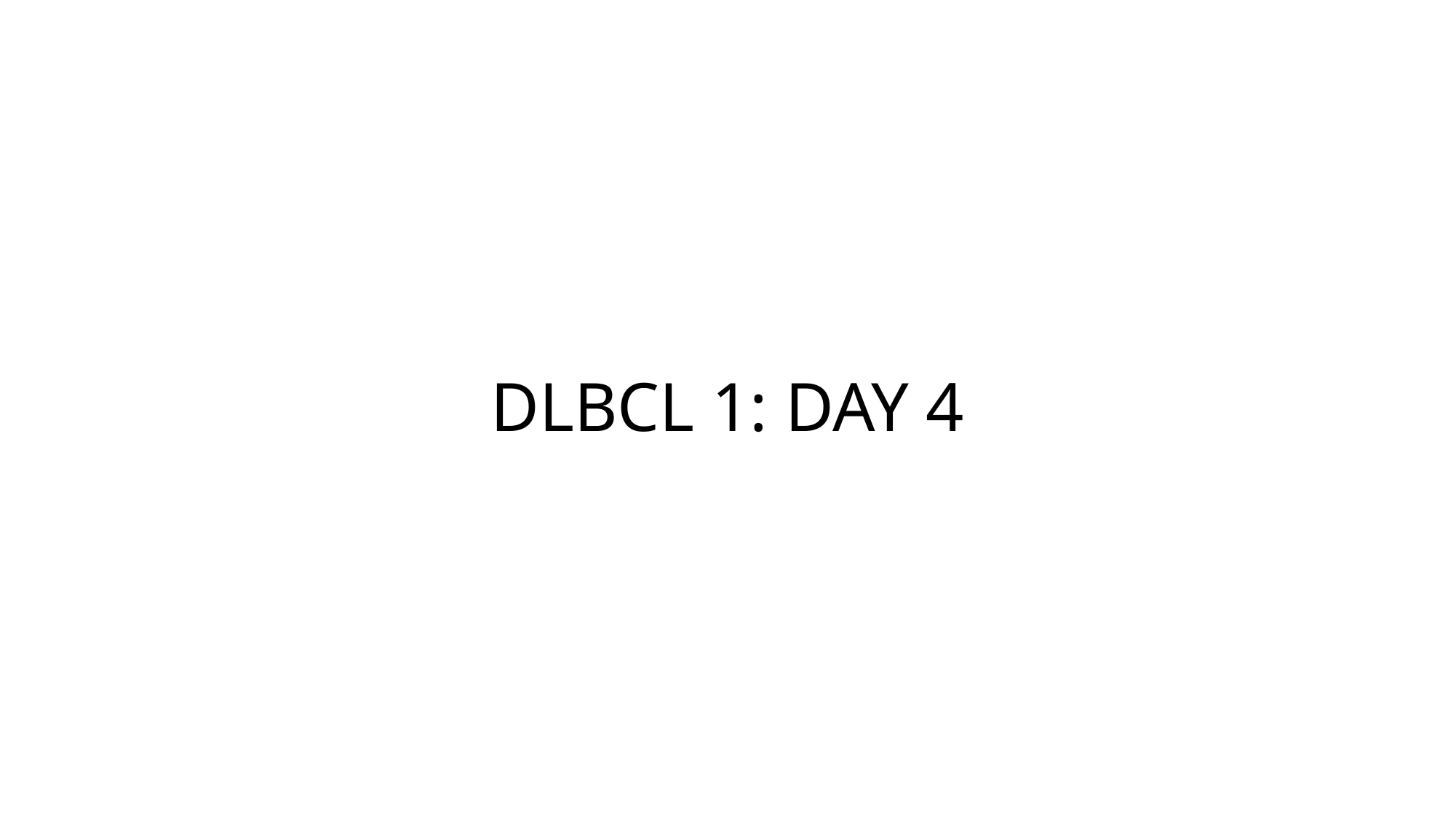

# DLBCL 1: DAY 4

## Slide 38
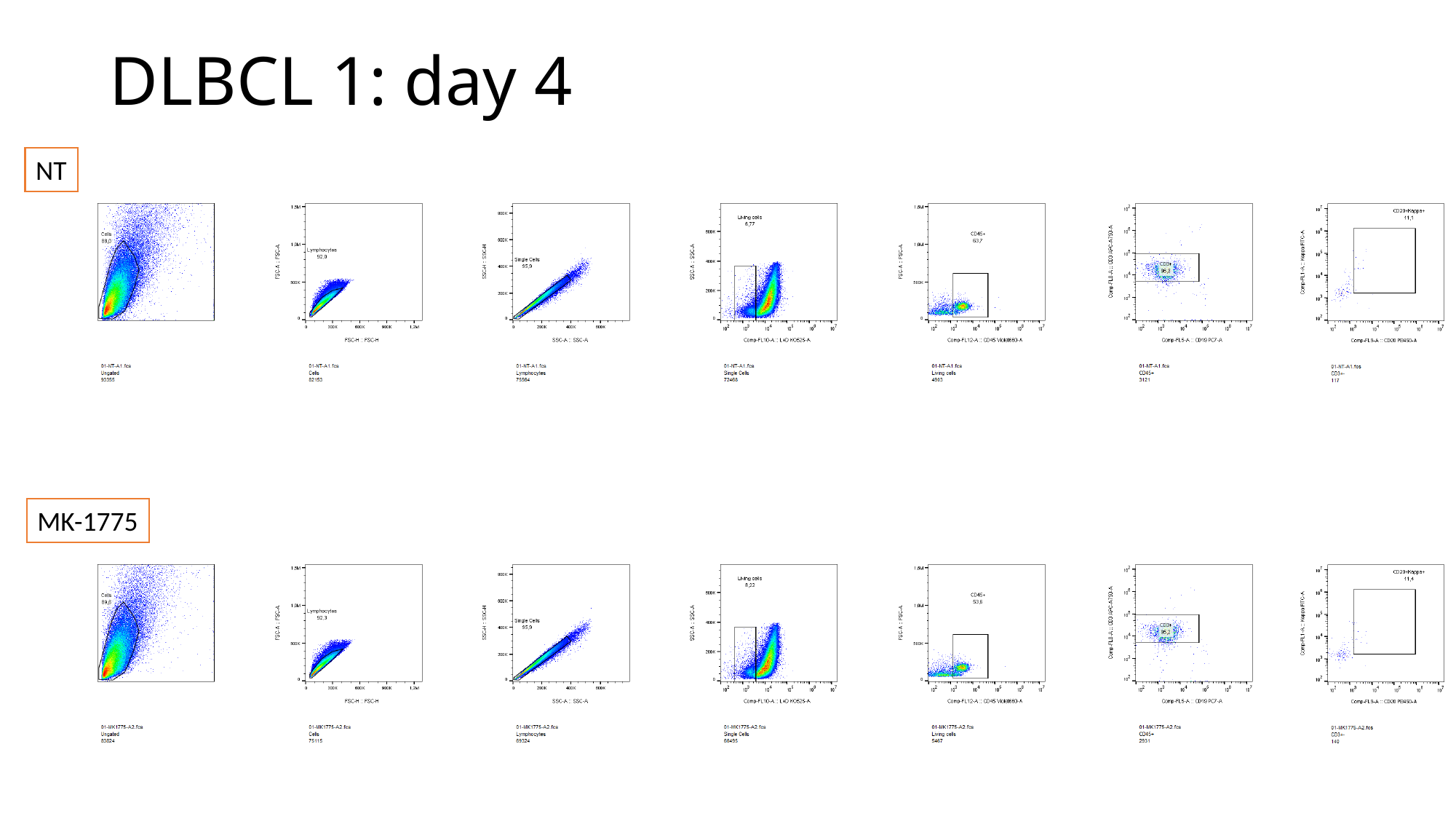

# DLBCL 1: day 4
NT
MK-1775

## Slide 39
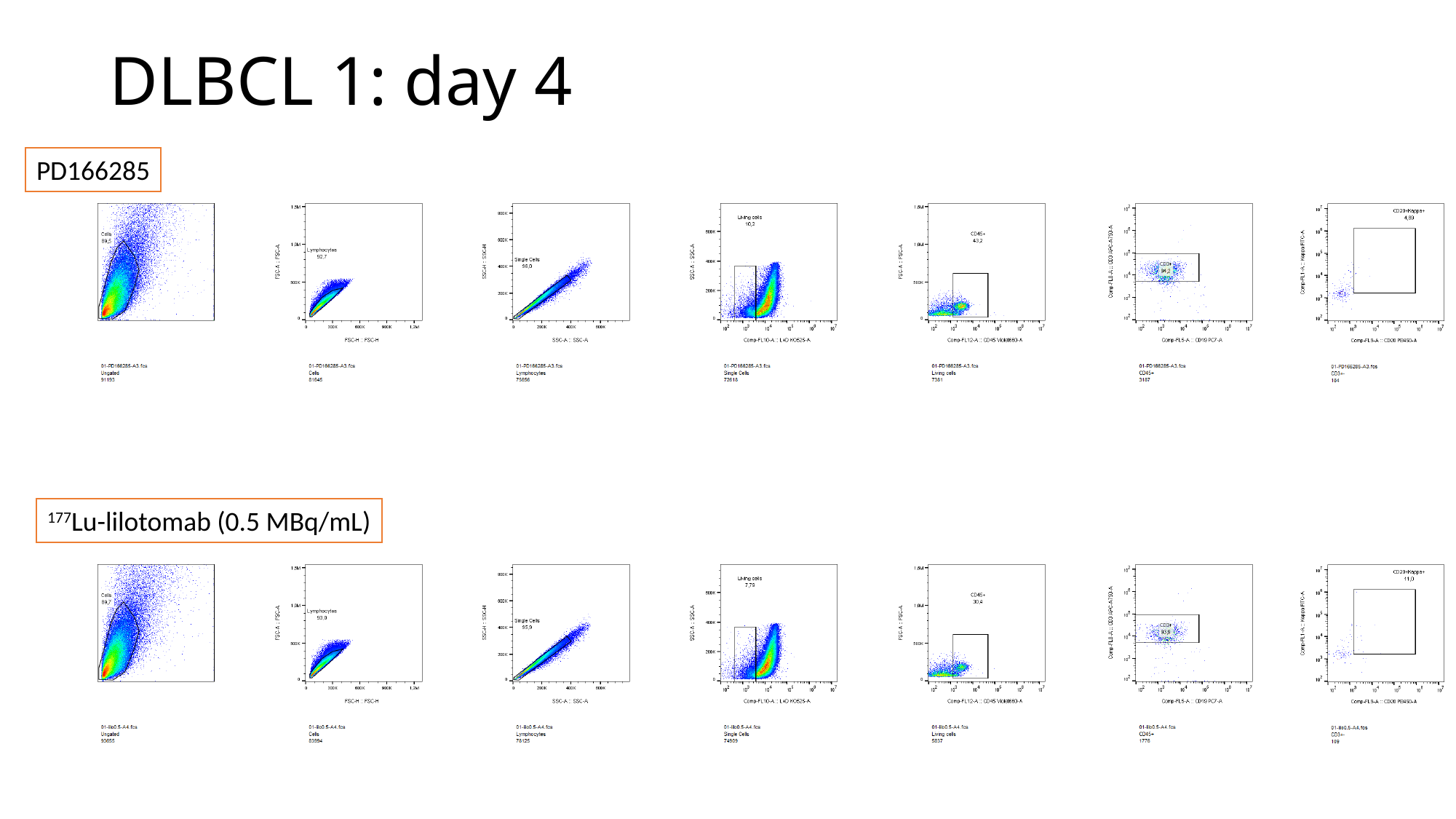

# DLBCL 1: day 4
PD166285
177Lu-lilotomab (0.5 MBq/mL)

## Slide 40
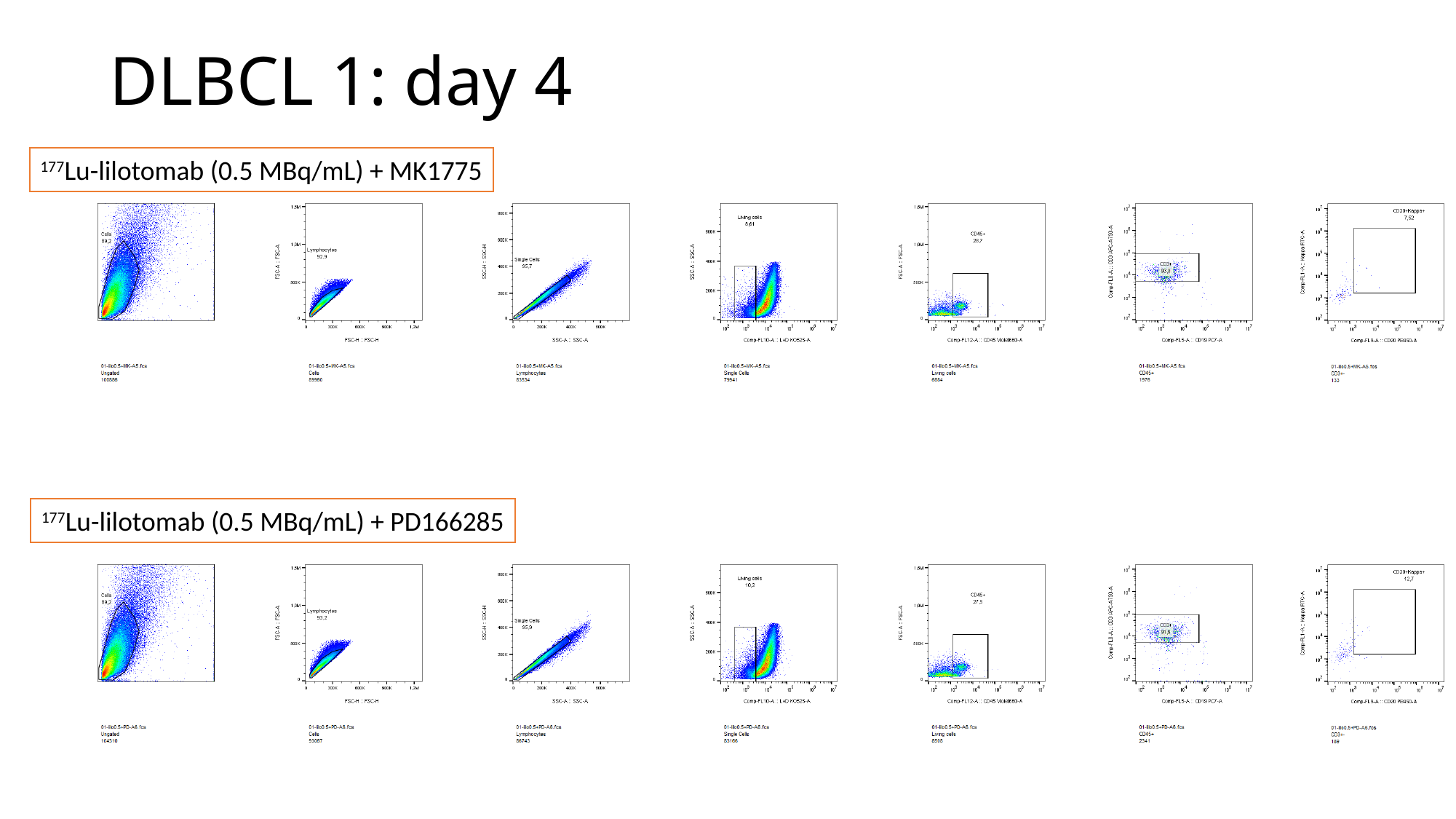

# DLBCL 1: day 4
177Lu-lilotomab (0.5 MBq/mL) + MK1775
177Lu-lilotomab (0.5 MBq/mL) + PD166285

## Slide 41
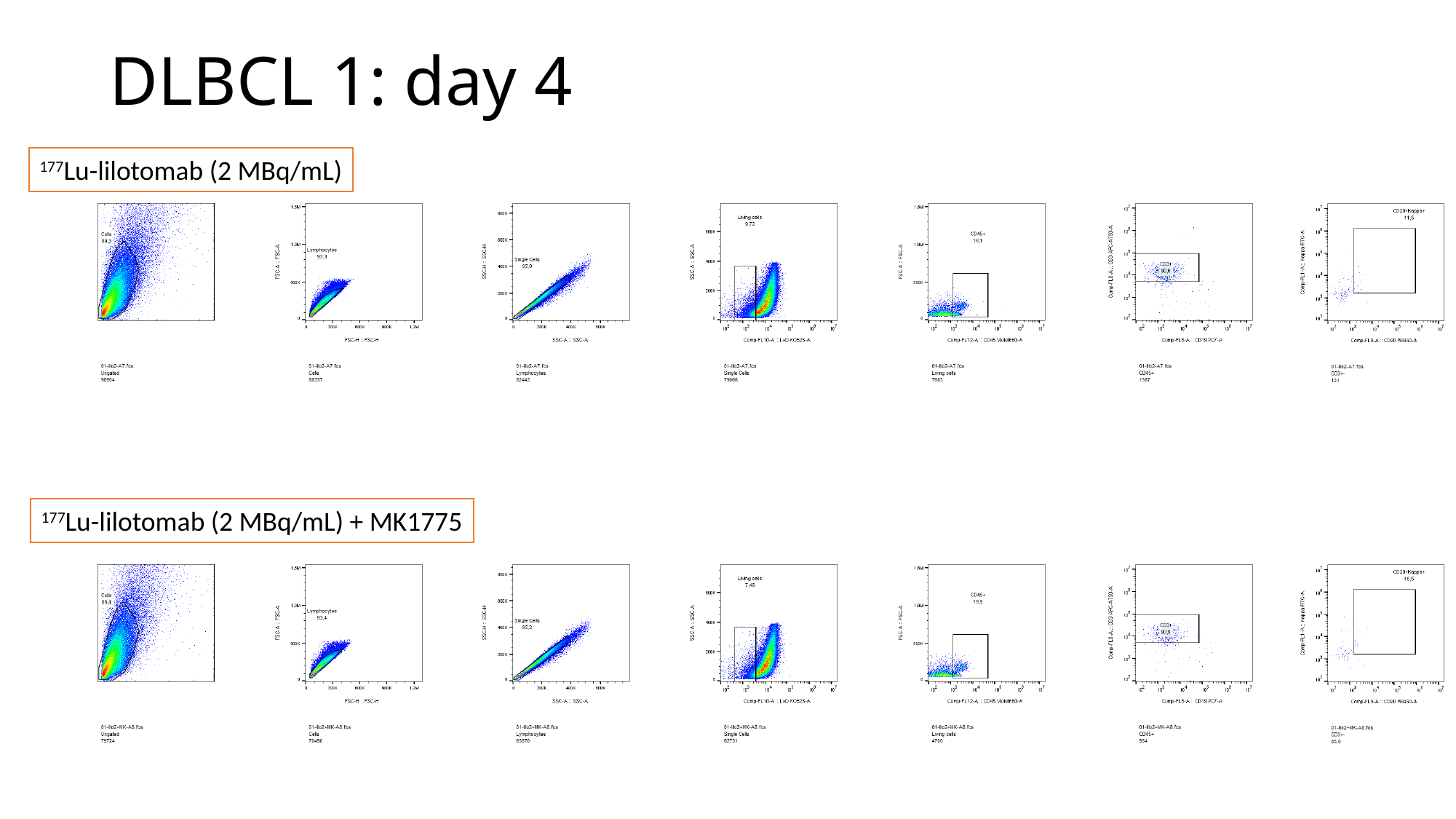

# DLBCL 1: day 4
177Lu-lilotomab (2 MBq/mL)
177Lu-lilotomab (2 MBq/mL) + MK1775

## Slide 42
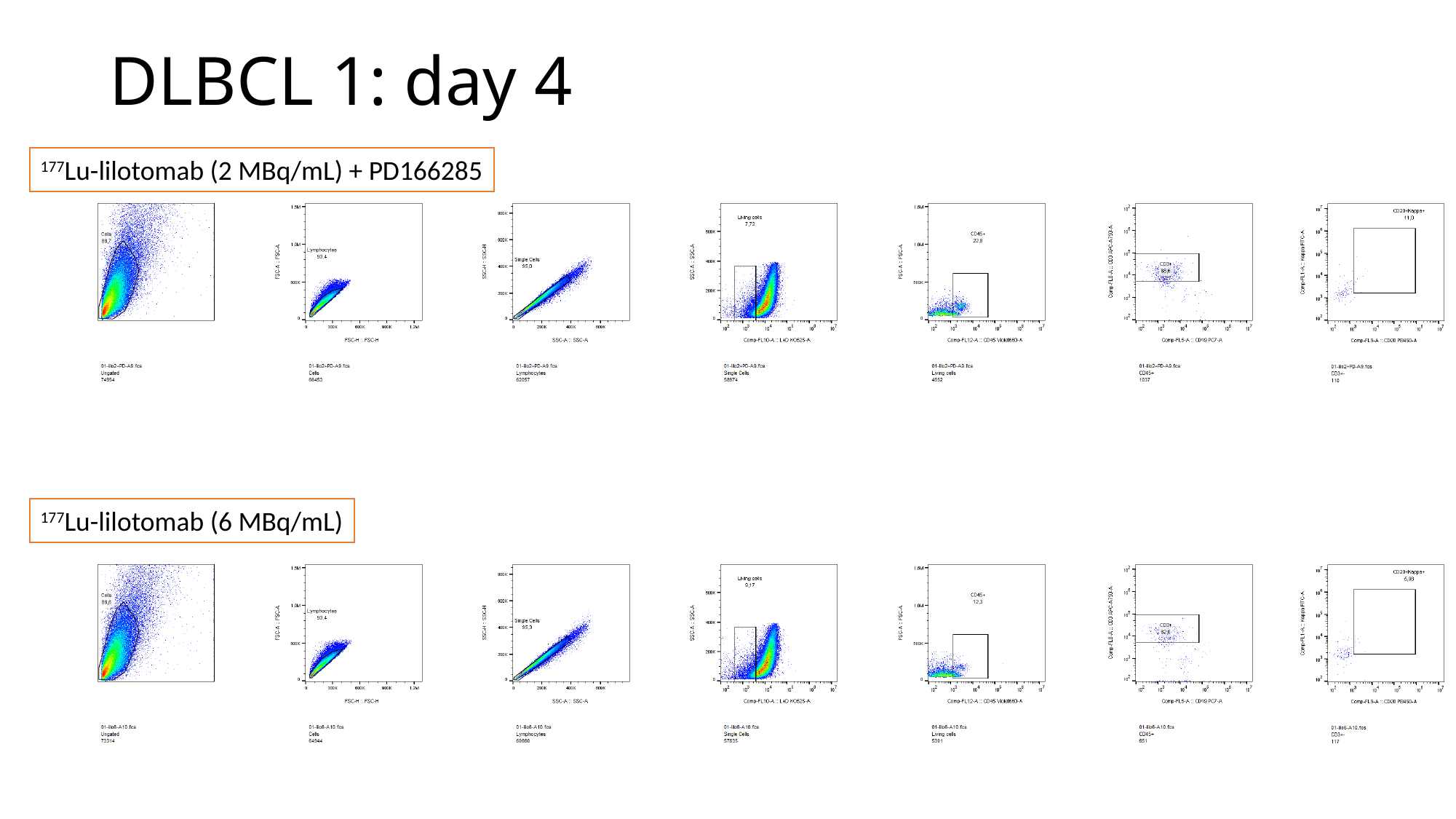

# DLBCL 1: day 4
177Lu-lilotomab (2 MBq/mL) + PD166285
177Lu-lilotomab (6 MBq/mL)

## Slide 43
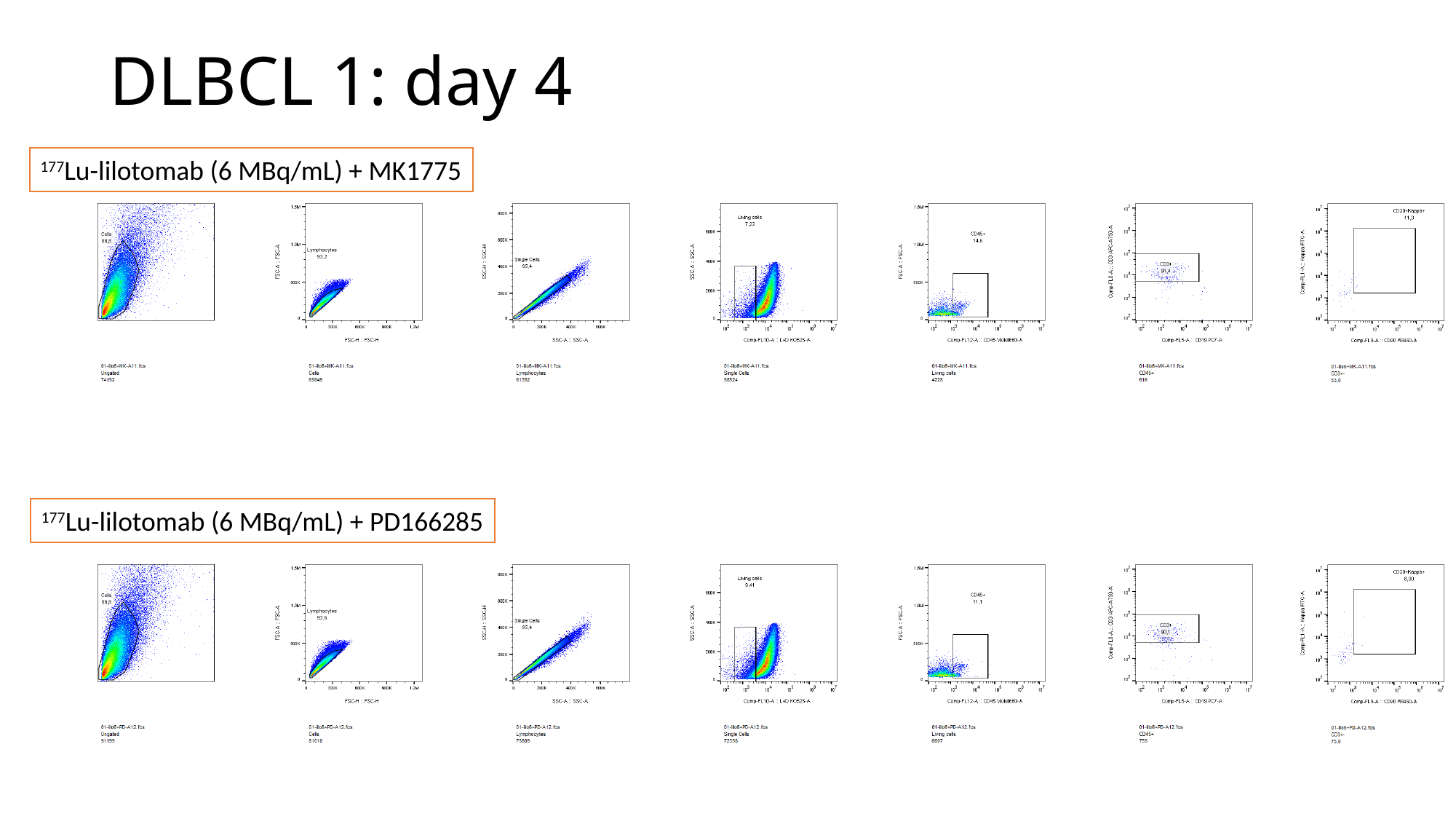

# DLBCL 1: day 4
177Lu-lilotomab (6 MBq/mL) + MK1775
177Lu-lilotomab (6 MBq/mL) + PD166285

## Slide 44
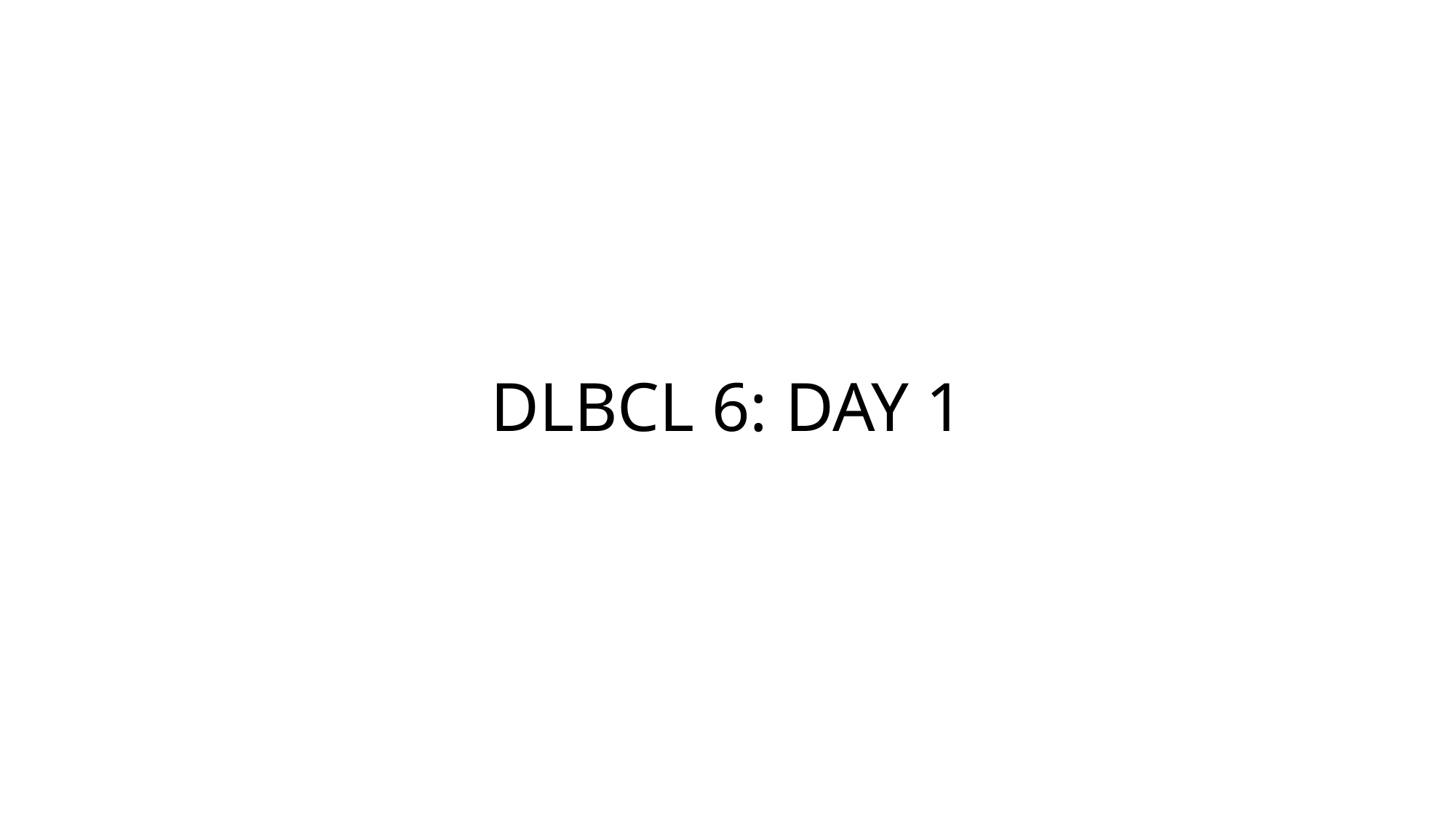

# DLBCL 6: DAY 1

## Slide 45
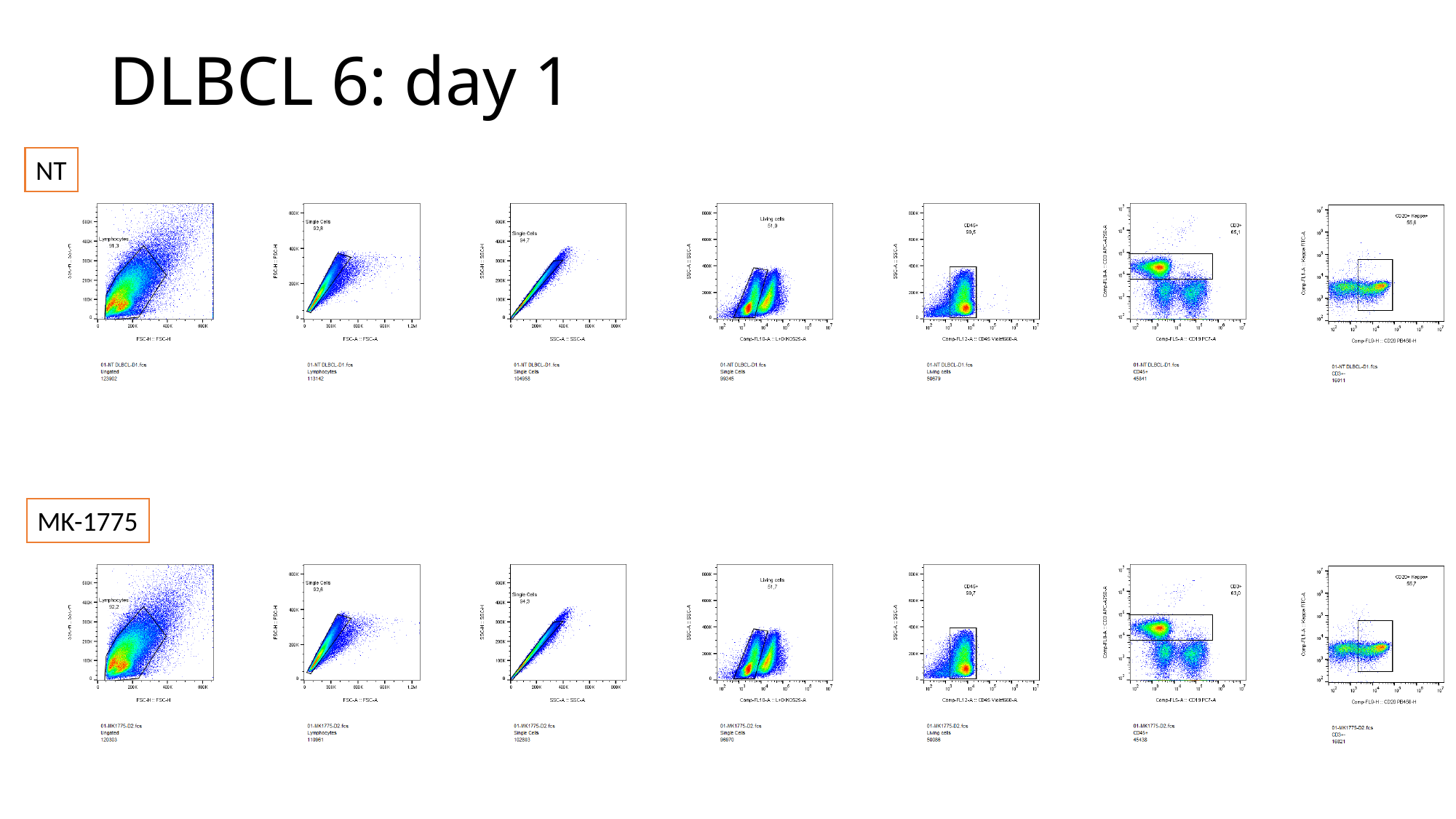

# DLBCL 6: day 1
NT
MK-1775

## Slide 46
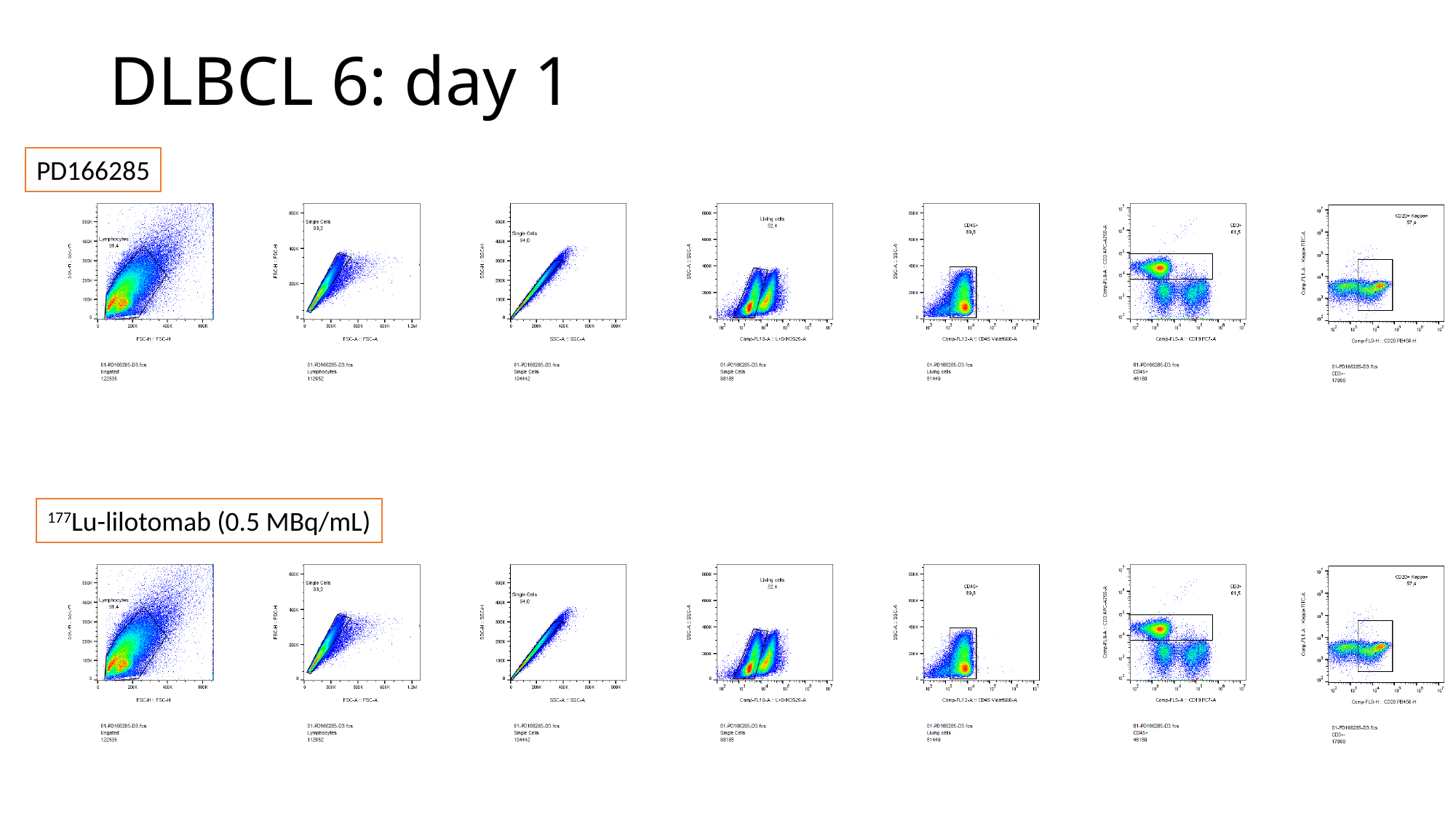

# DLBCL 6: day 1
PD166285
177Lu-lilotomab (0.5 MBq/mL)

## Slide 47
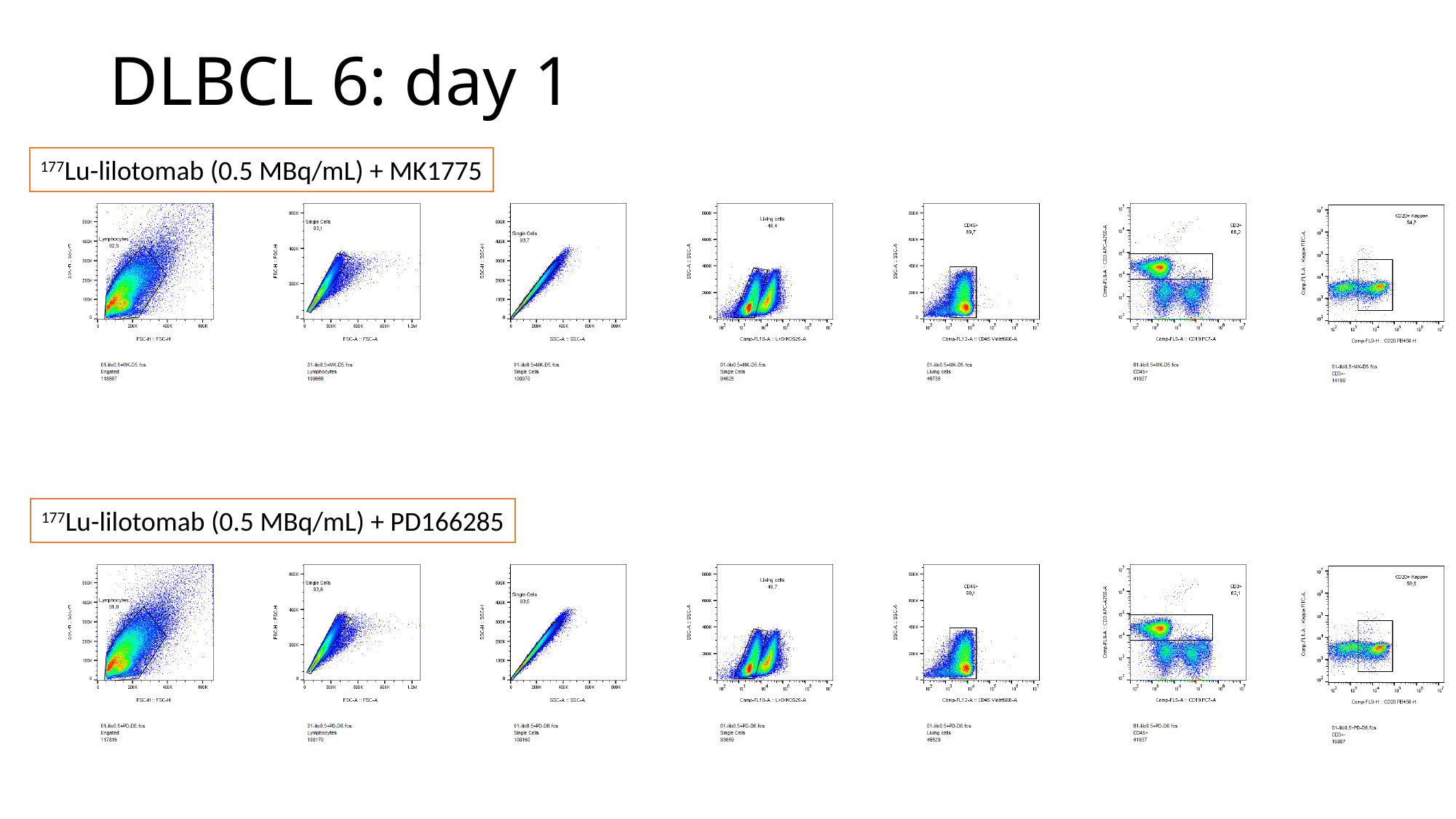

# DLBCL 6: day 1
177Lu-lilotomab (0.5 MBq/mL) + MK1775
177Lu-lilotomab (0.5 MBq/mL) + PD166285

## Slide 48
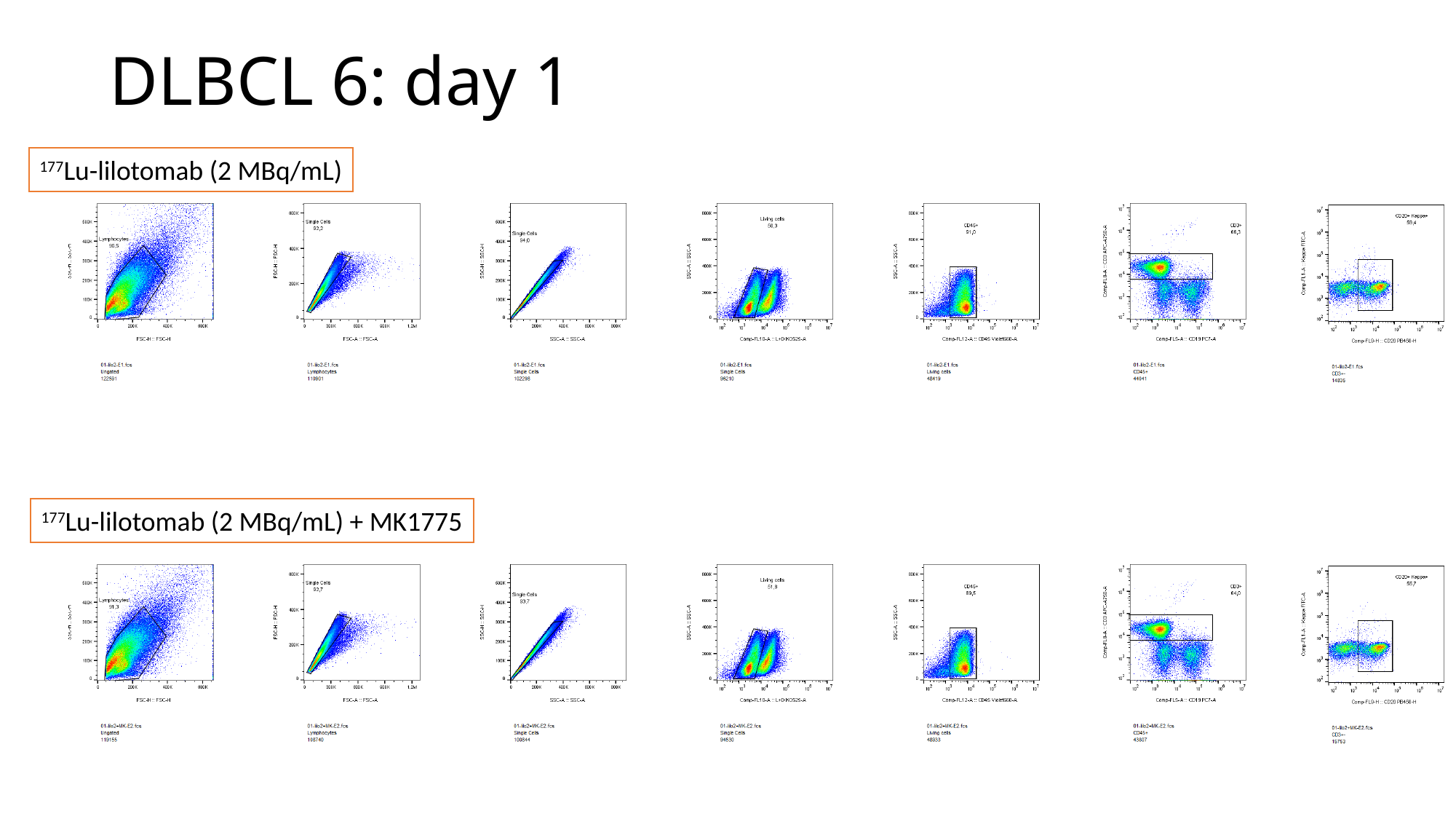

# DLBCL 6: day 1
177Lu-lilotomab (2 MBq/mL)
177Lu-lilotomab (2 MBq/mL) + MK1775

## Slide 49
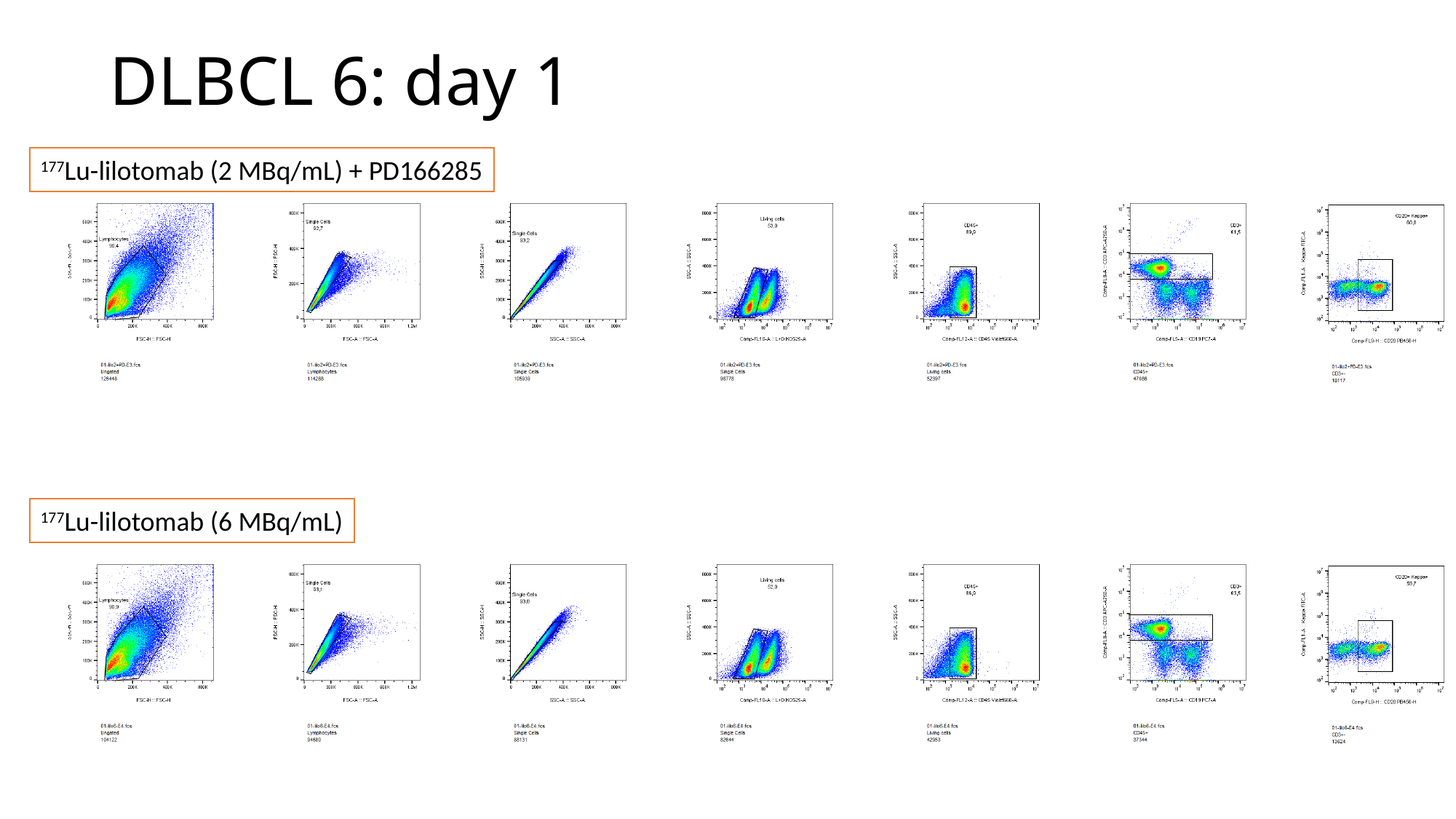

# DLBCL 6: day 1
177Lu-lilotomab (2 MBq/mL) + PD166285
177Lu-lilotomab (6 MBq/mL)

## Slide 50
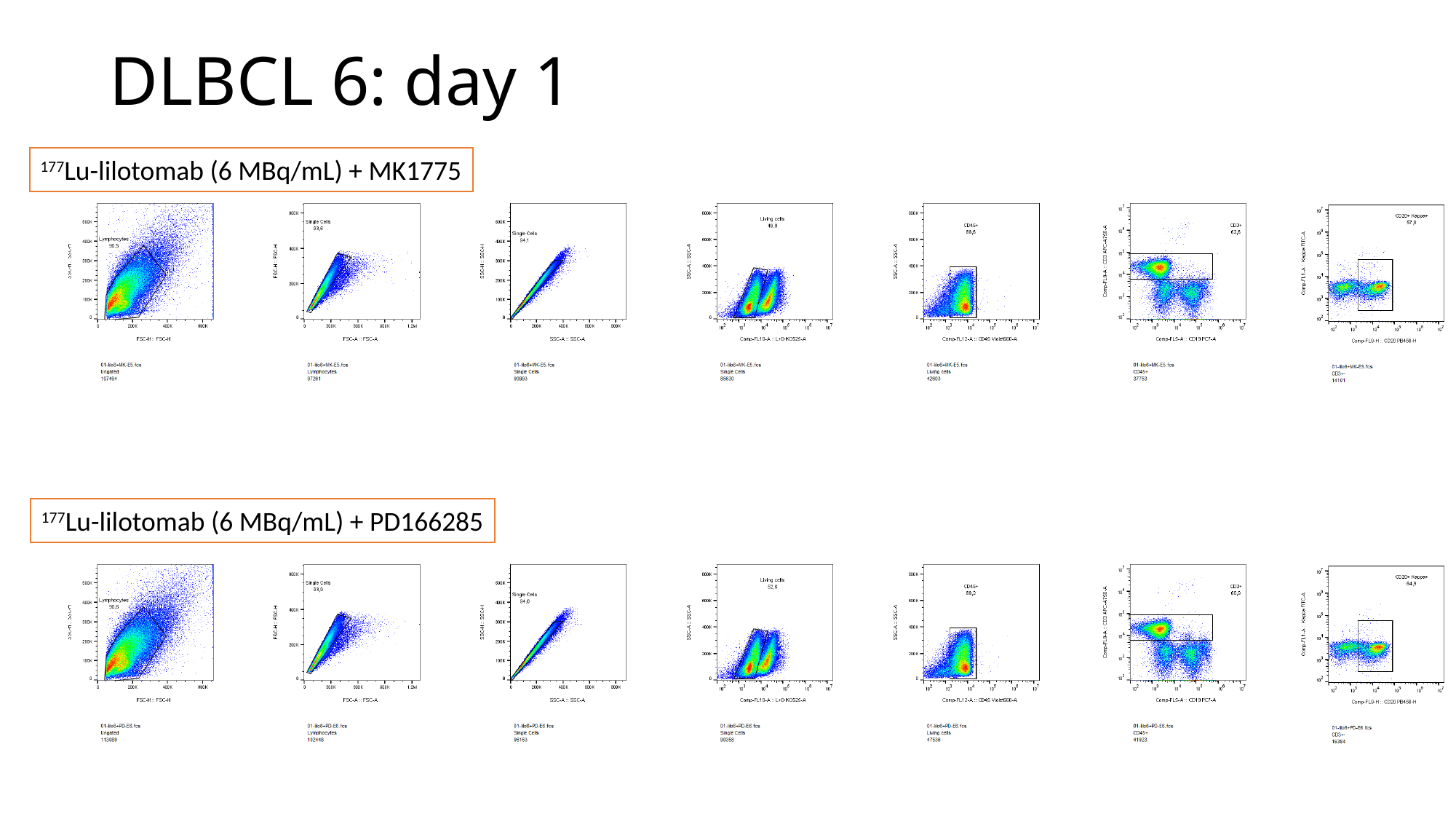

# DLBCL 6: day 1
177Lu-lilotomab (6 MBq/mL) + MK1775
177Lu-lilotomab (6 MBq/mL) + PD166285

## Slide 51
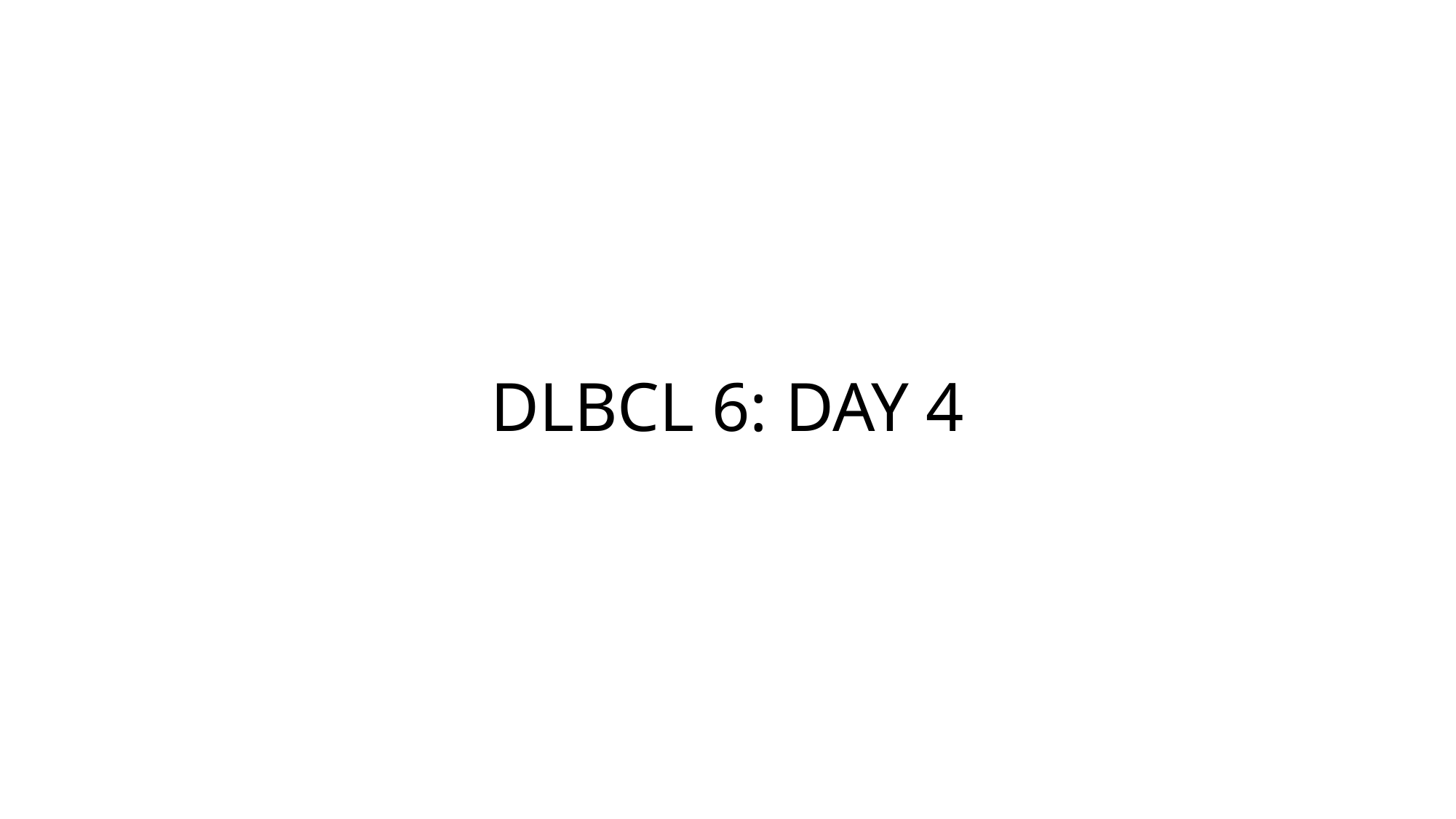

# DLBCL 6: DAY 4

## Slide 52
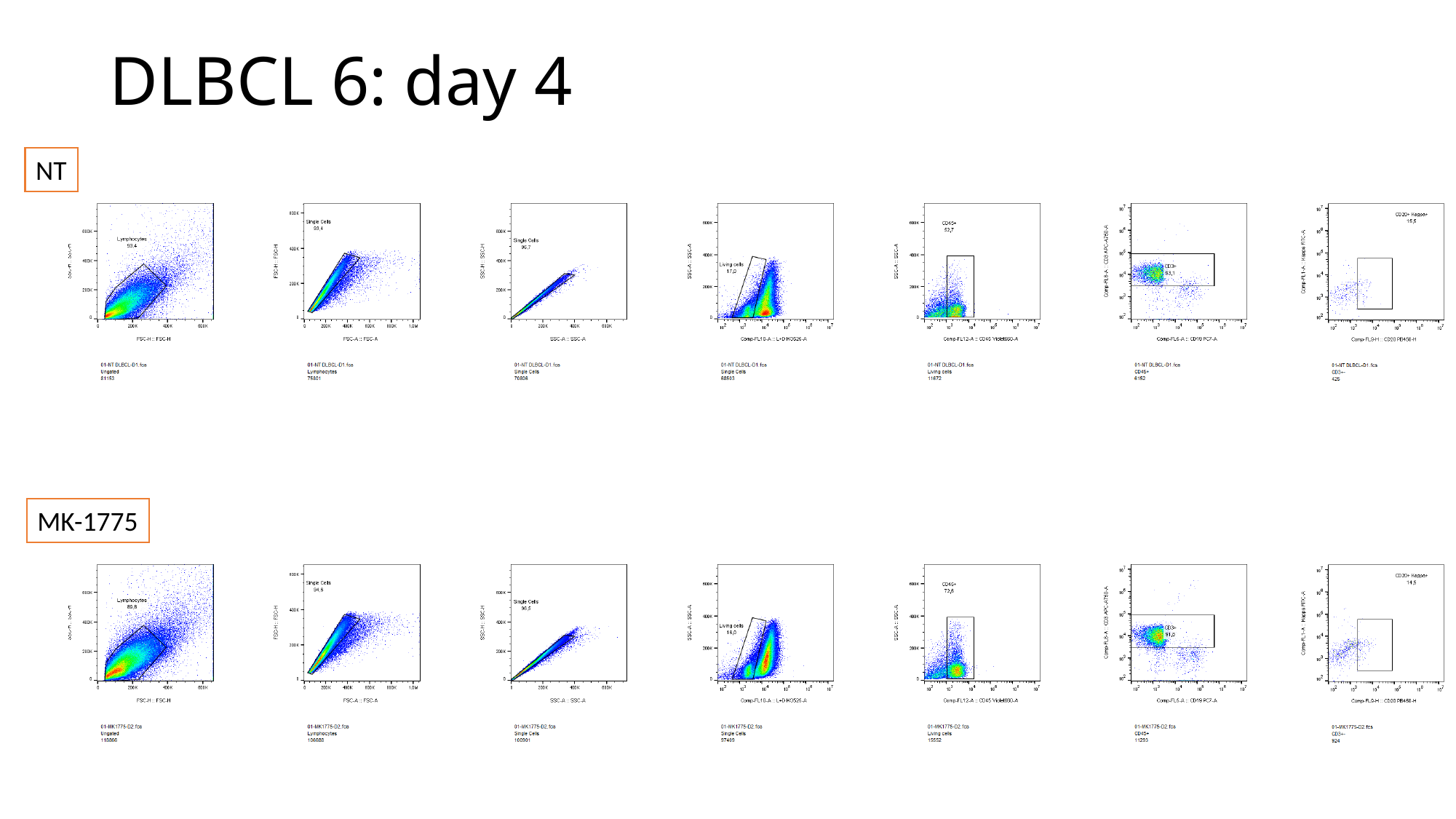

# DLBCL 6: day 4
NT
MK-1775

## Slide 53
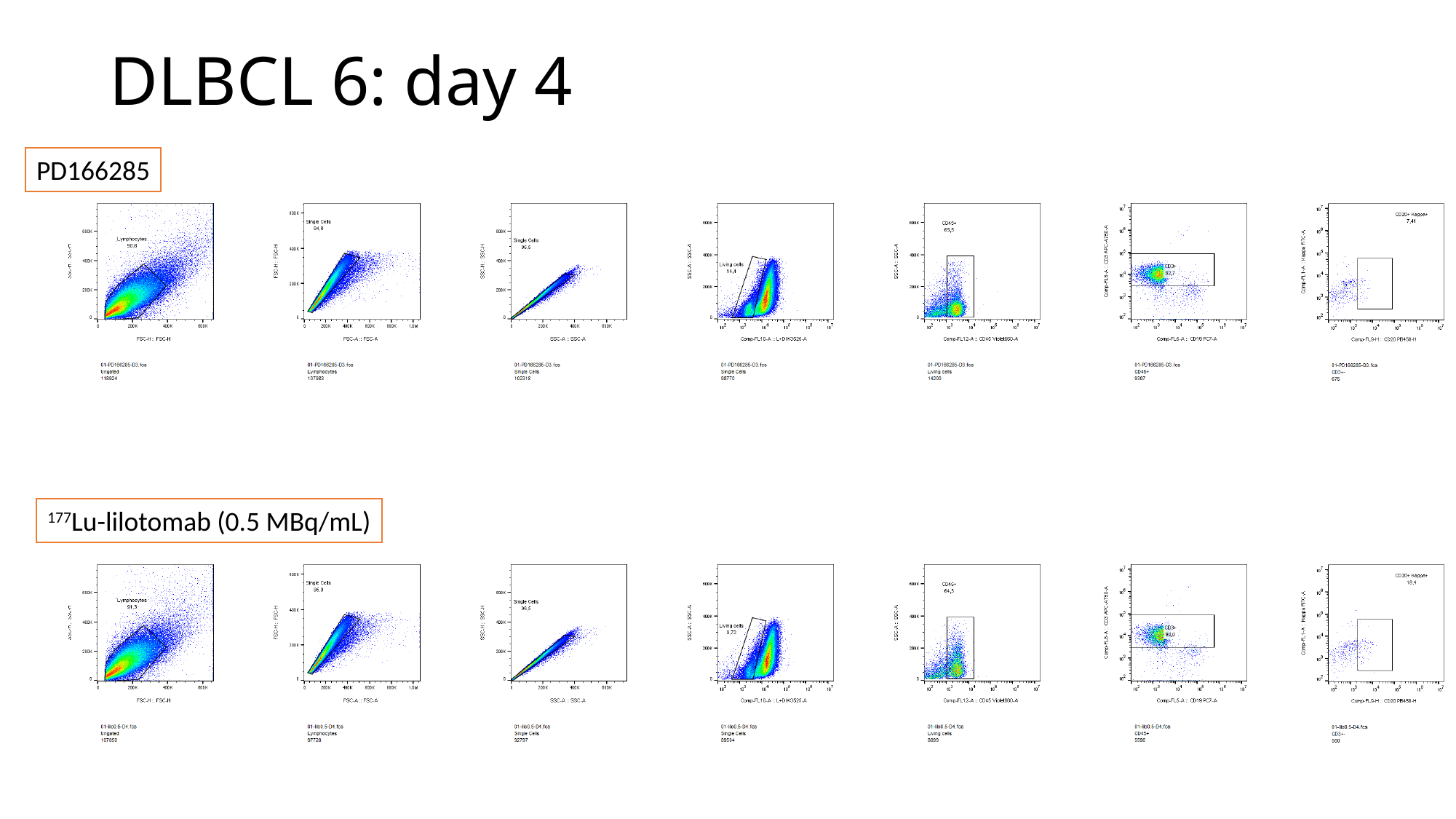

# DLBCL 6: day 4
PD166285
177Lu-lilotomab (0.5 MBq/mL)

## Slide 54
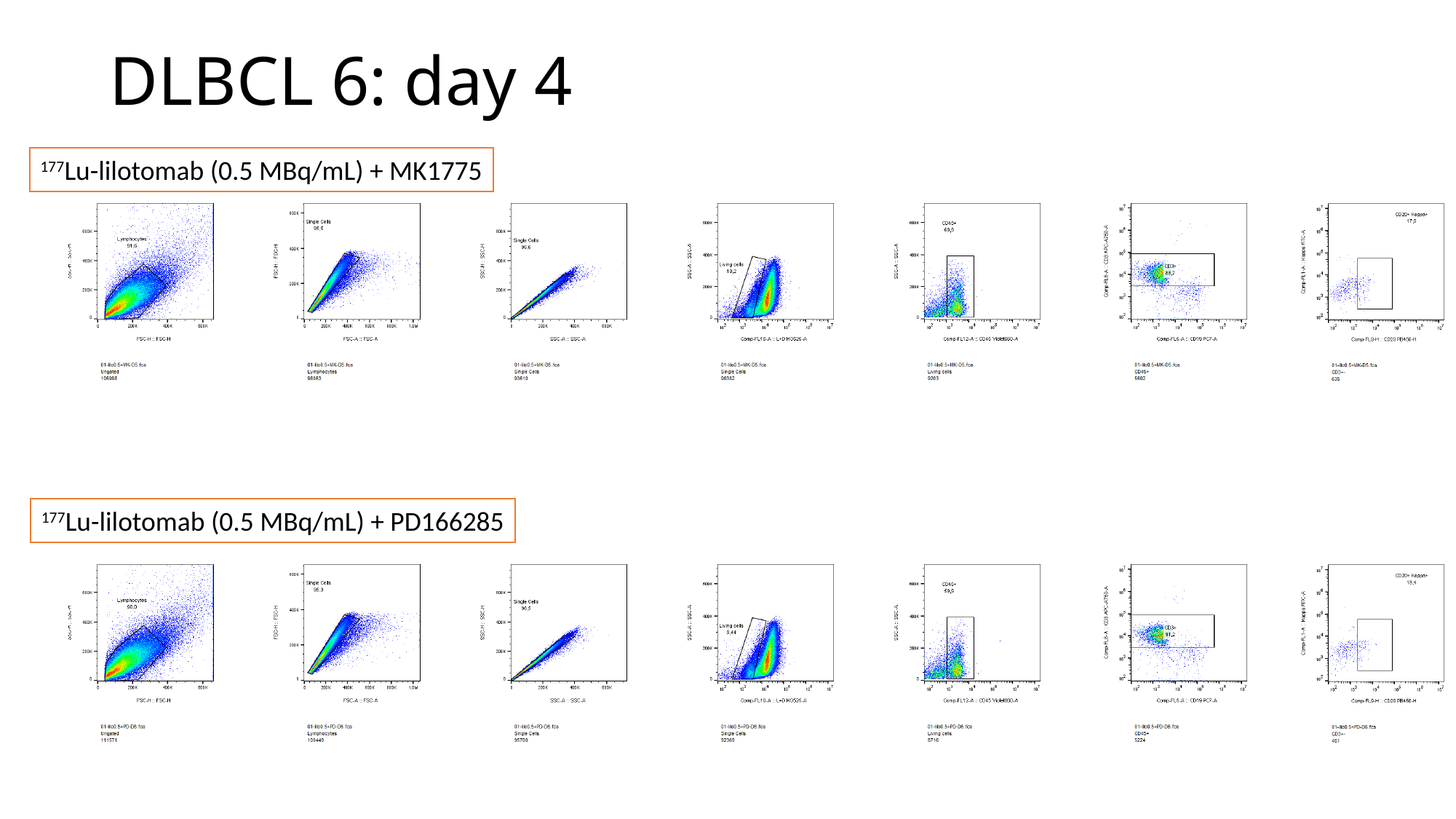

# DLBCL 6: day 4
177Lu-lilotomab (0.5 MBq/mL) + MK1775
177Lu-lilotomab (0.5 MBq/mL) + PD166285

## Slide 55
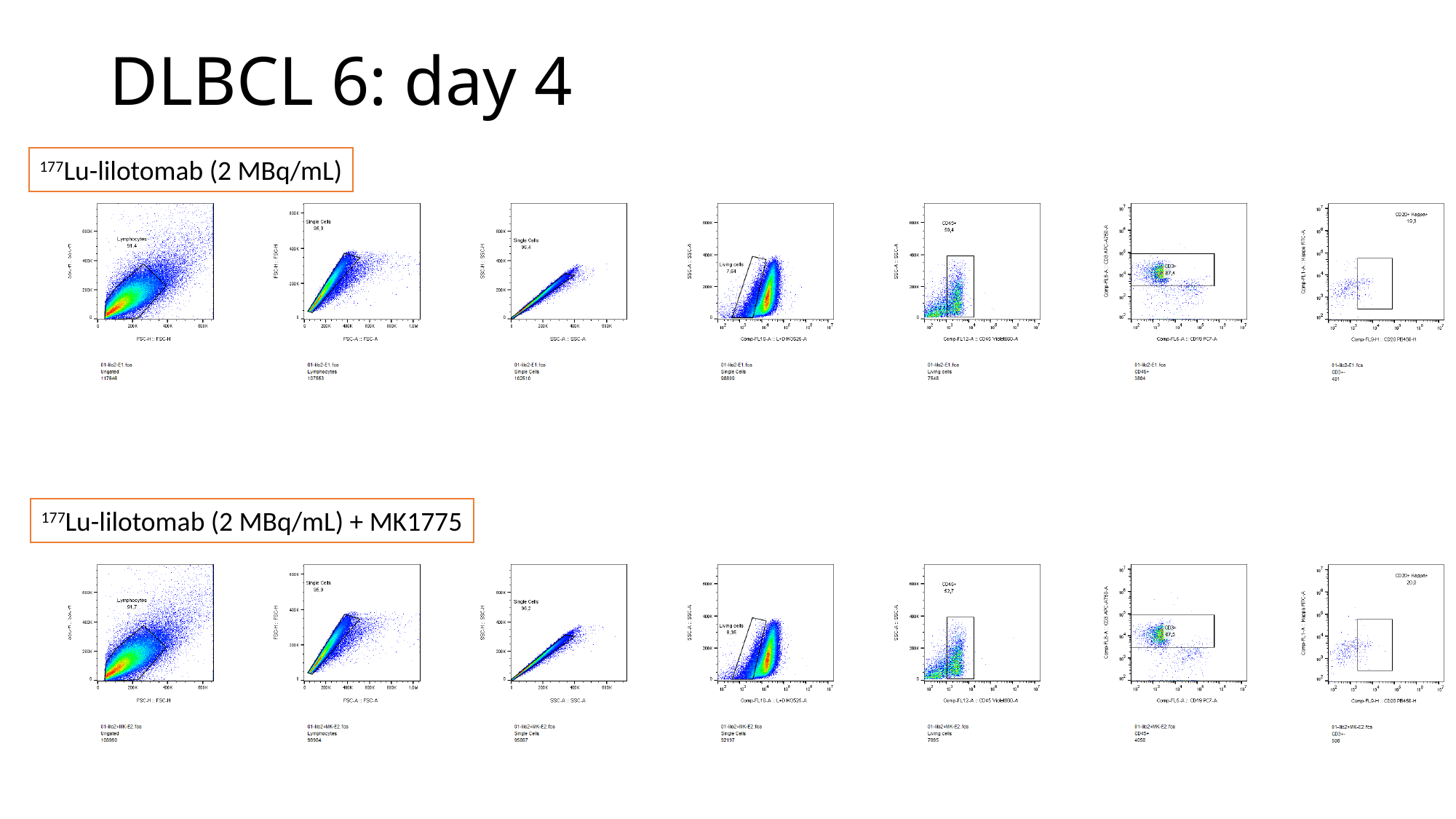

# DLBCL 6: day 4
177Lu-lilotomab (2 MBq/mL)
177Lu-lilotomab (2 MBq/mL) + MK1775

## Slide 56
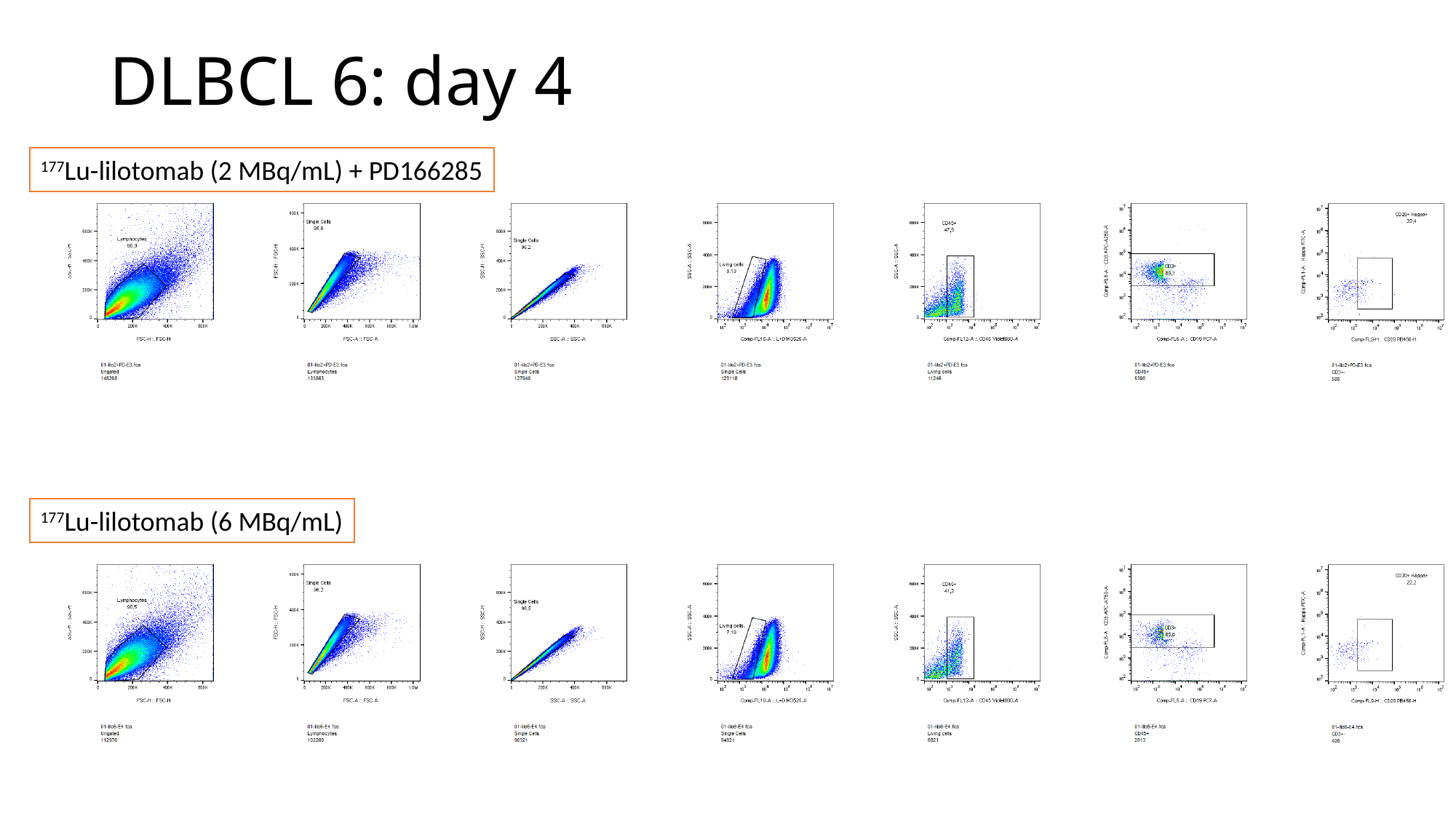

# DLBCL 6: day 4
177Lu-lilotomab (2 MBq/mL) + PD166285
177Lu-lilotomab (6 MBq/mL)

## Slide 57
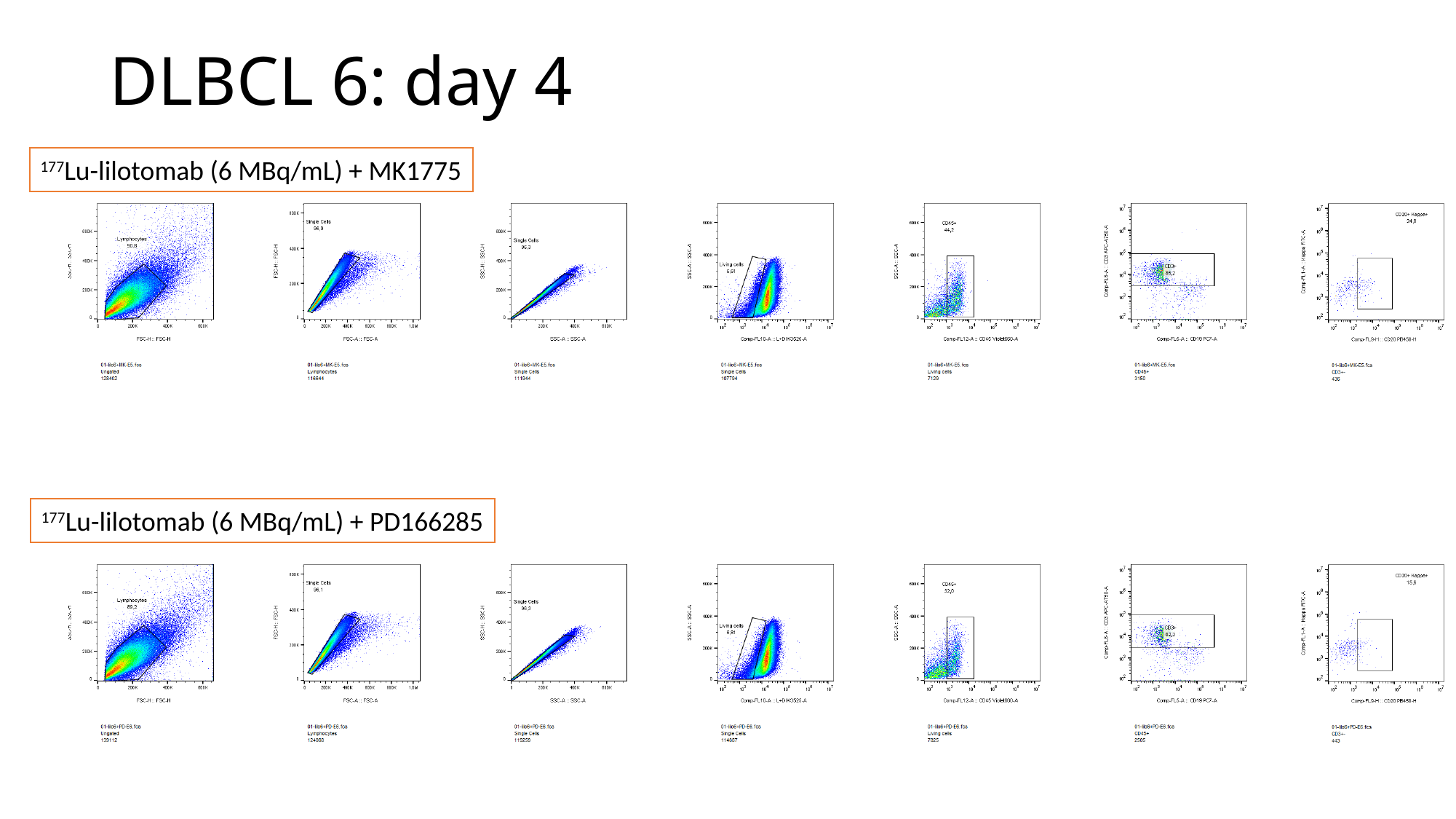

# DLBCL 6: day 4
177Lu-lilotomab (6 MBq/mL) + MK1775
177Lu-lilotomab (6 MBq/mL) + PD166285
