# Supplementary material for: The therapeutic effectiveness of 177Lu-lilotomab in B-cell non-Hodgkin lymphoma involves modulation of G2/M cell cycle arrest
Source: Leukemia. 2019 Dec 13;34(5):1315–28. doi: 10.1038/s41375-019-0677-4 (PMC7192854; doi:10.1038/s41375-019-0677-4)
Supplement: Supplementary file 14 — Dataset 2 [file 41375_2019_677_MOESM14_ESM.pptx]

## Slide 1
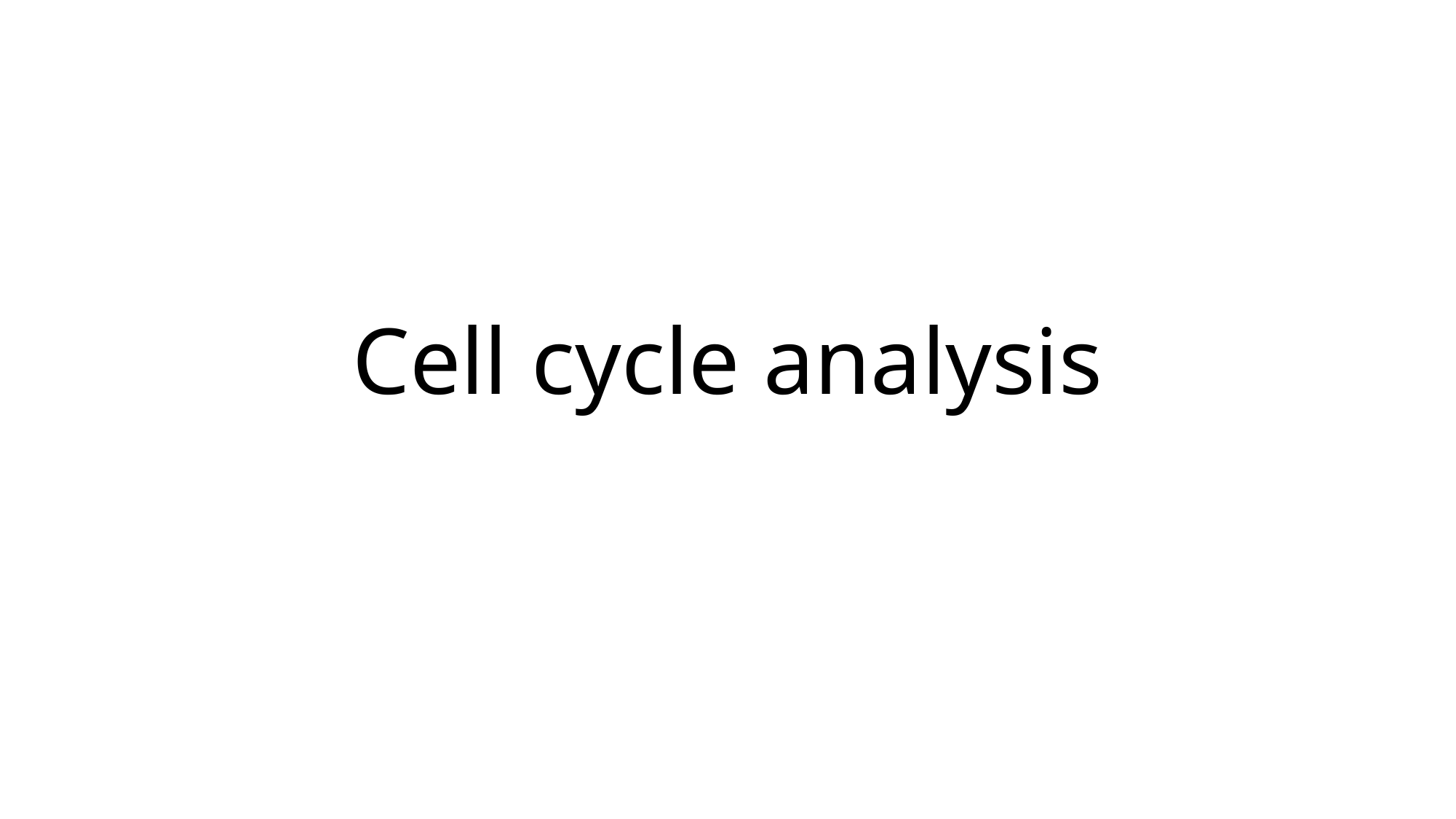

# Cell cycle analysis

## Slide 2
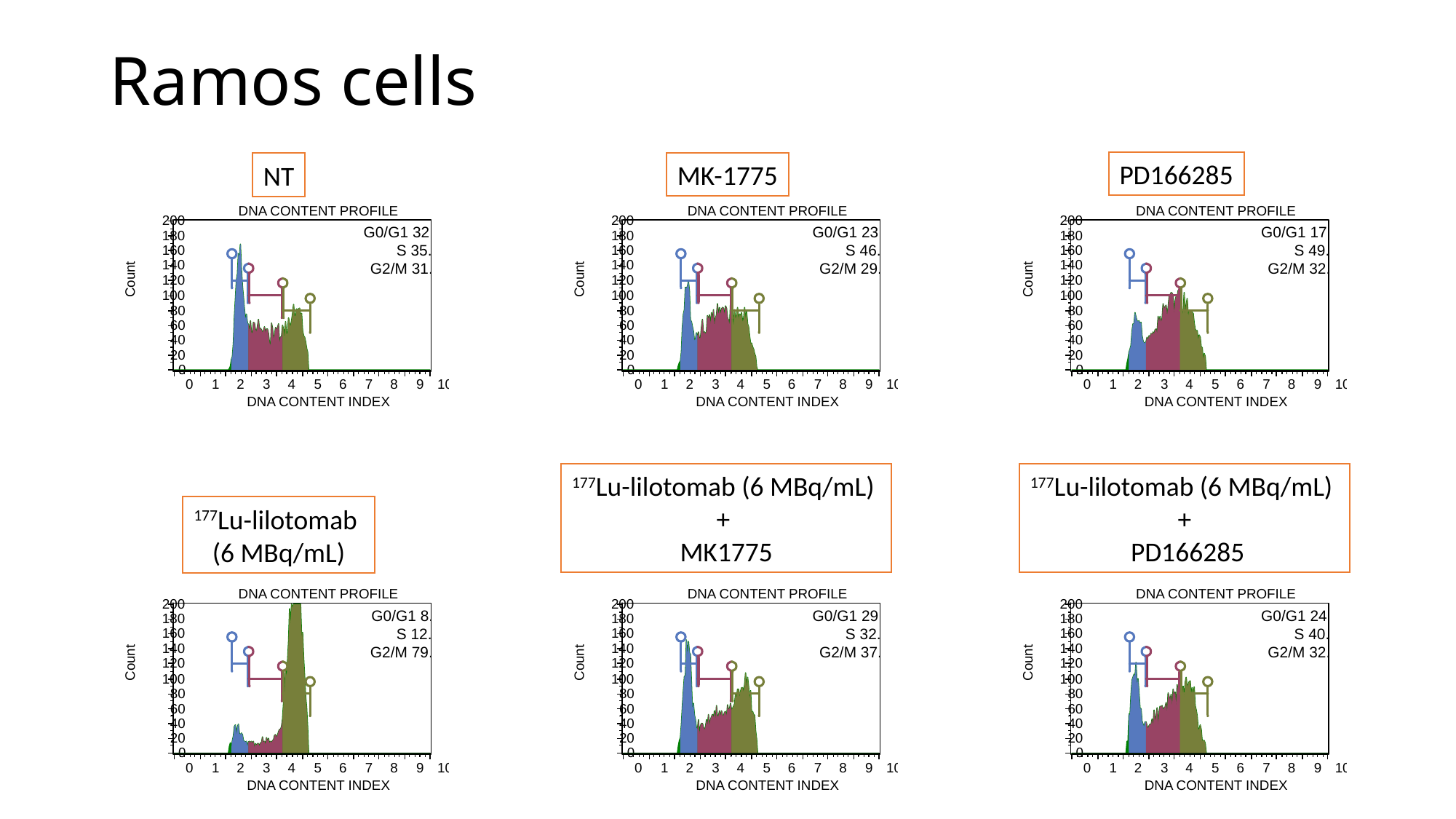

# Ramos cells
PD166285
MK-1775
NT
177Lu-lilotomab (6 MBq/mL)
+
MK1775
177Lu-lilotomab (6 MBq/mL)
+
 PD166285
177Lu-lilotomab
(6 MBq/mL)

## Slide 3
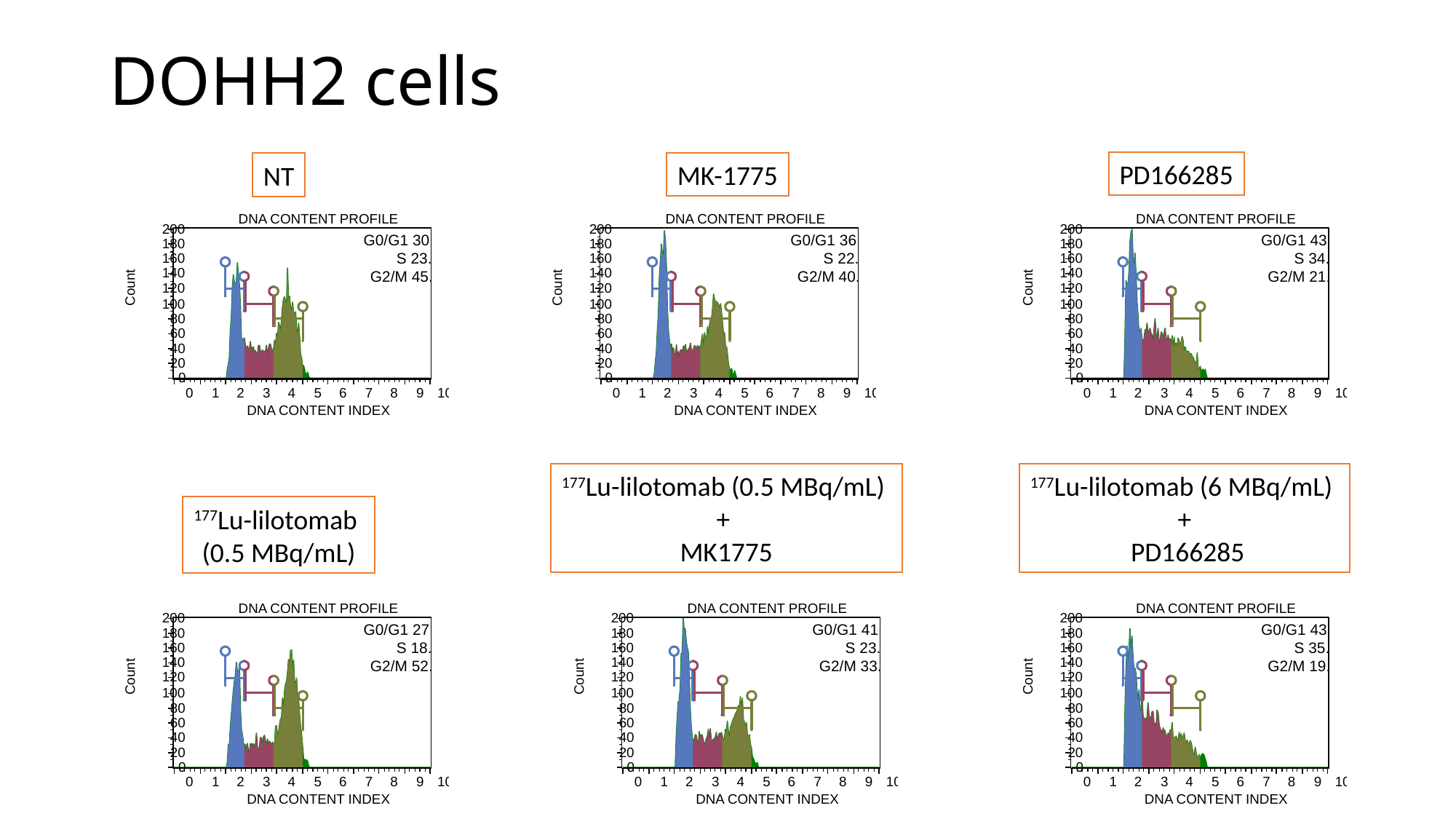

# DOHH2 cells
PD166285
MK-1775
NT
177Lu-lilotomab (0.5 MBq/mL)
+
MK1775
177Lu-lilotomab (6 MBq/mL)
+
 PD166285
177Lu-lilotomab
(0.5 MBq/mL)

## Slide 4
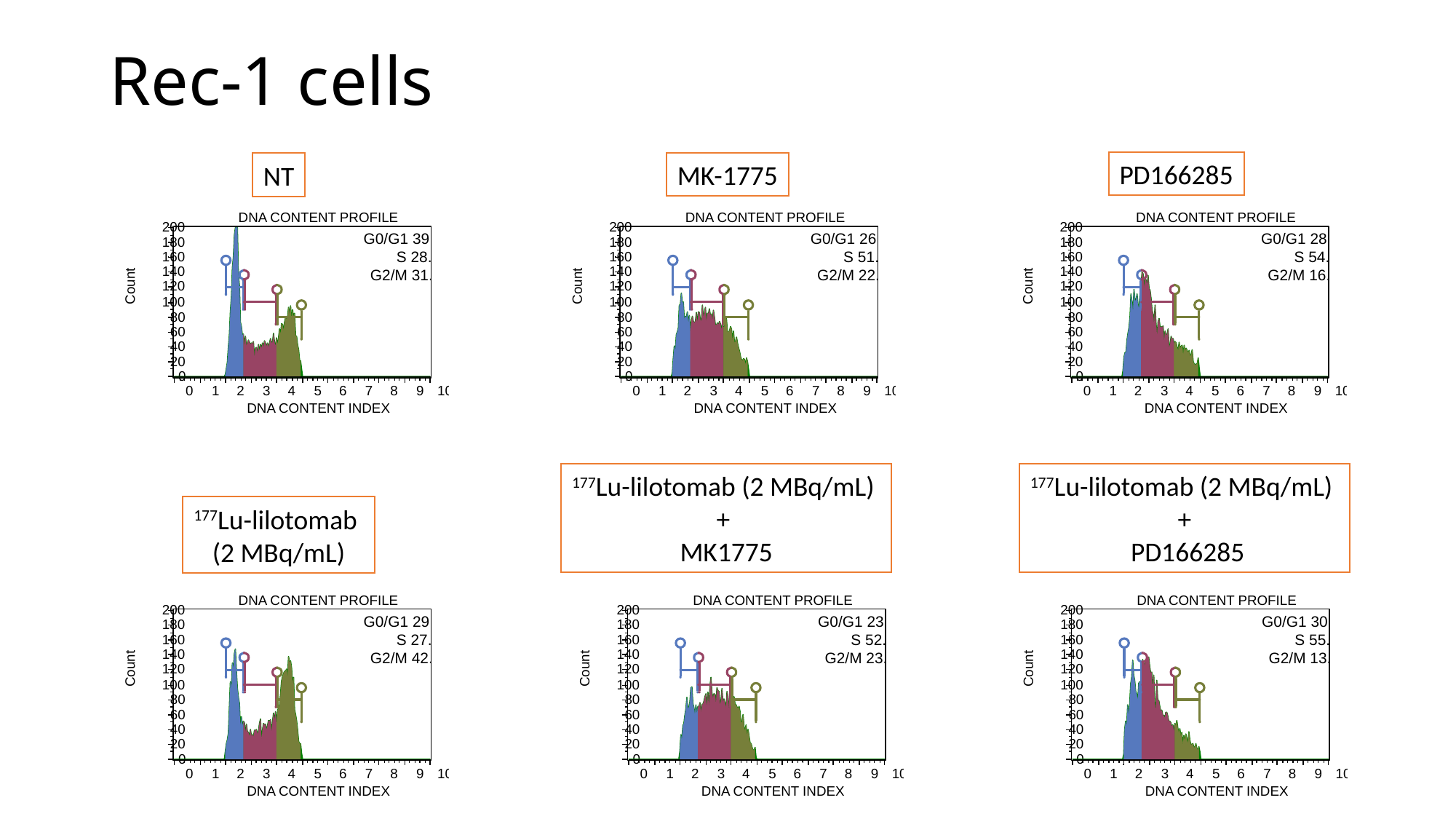

# Rec-1 cells
PD166285
MK-1775
NT
177Lu-lilotomab (2 MBq/mL)
+
MK1775
177Lu-lilotomab (2 MBq/mL)
+
 PD166285
177Lu-lilotomab
(2 MBq/mL)

## Slide 5
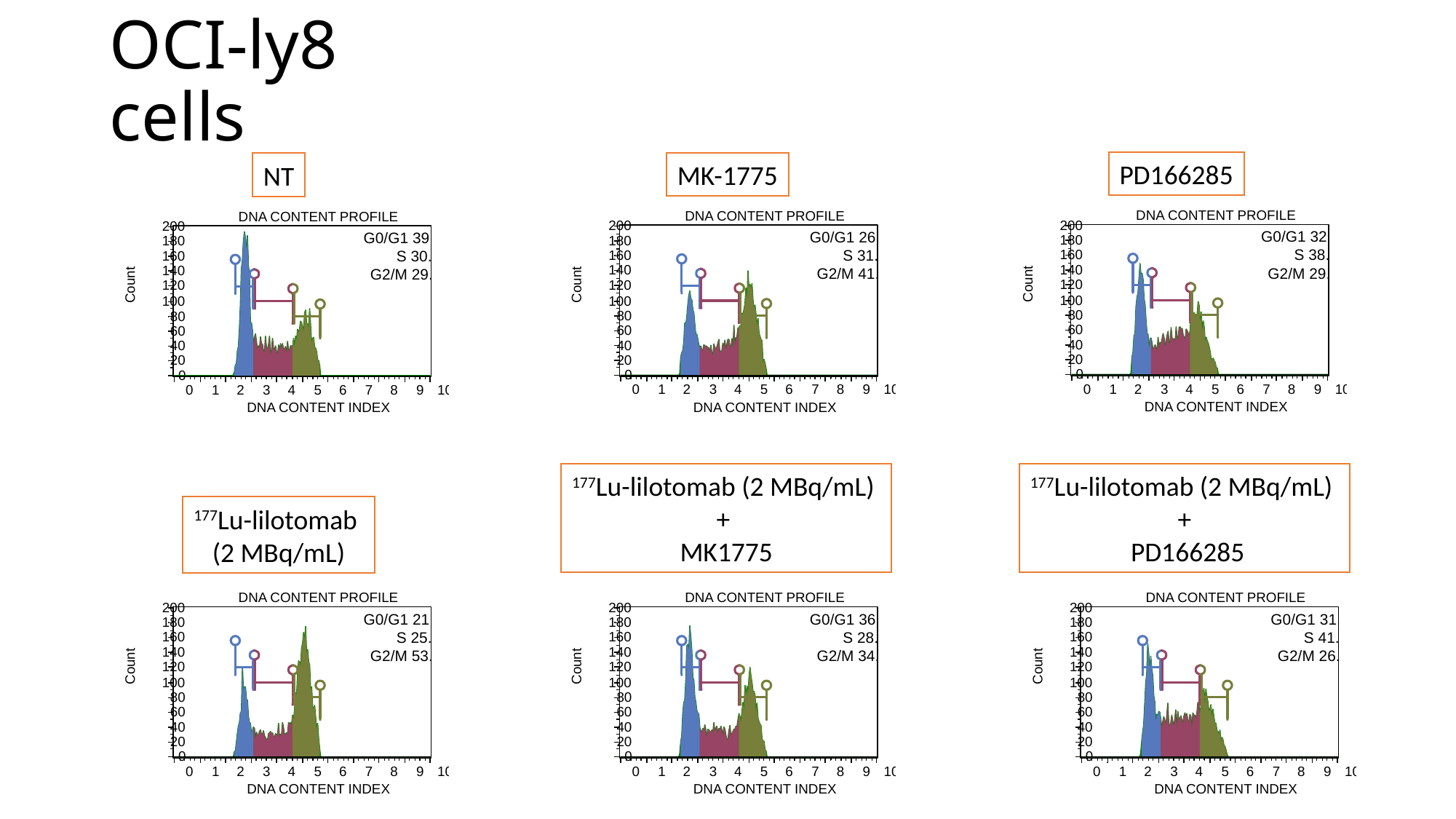

# OCI-ly8 cells
PD166285
MK-1775
NT
177Lu-lilotomab (2 MBq/mL)
+
MK1775
177Lu-lilotomab (2 MBq/mL)
+
 PD166285
177Lu-lilotomab
(2 MBq/mL)

## Slide 6
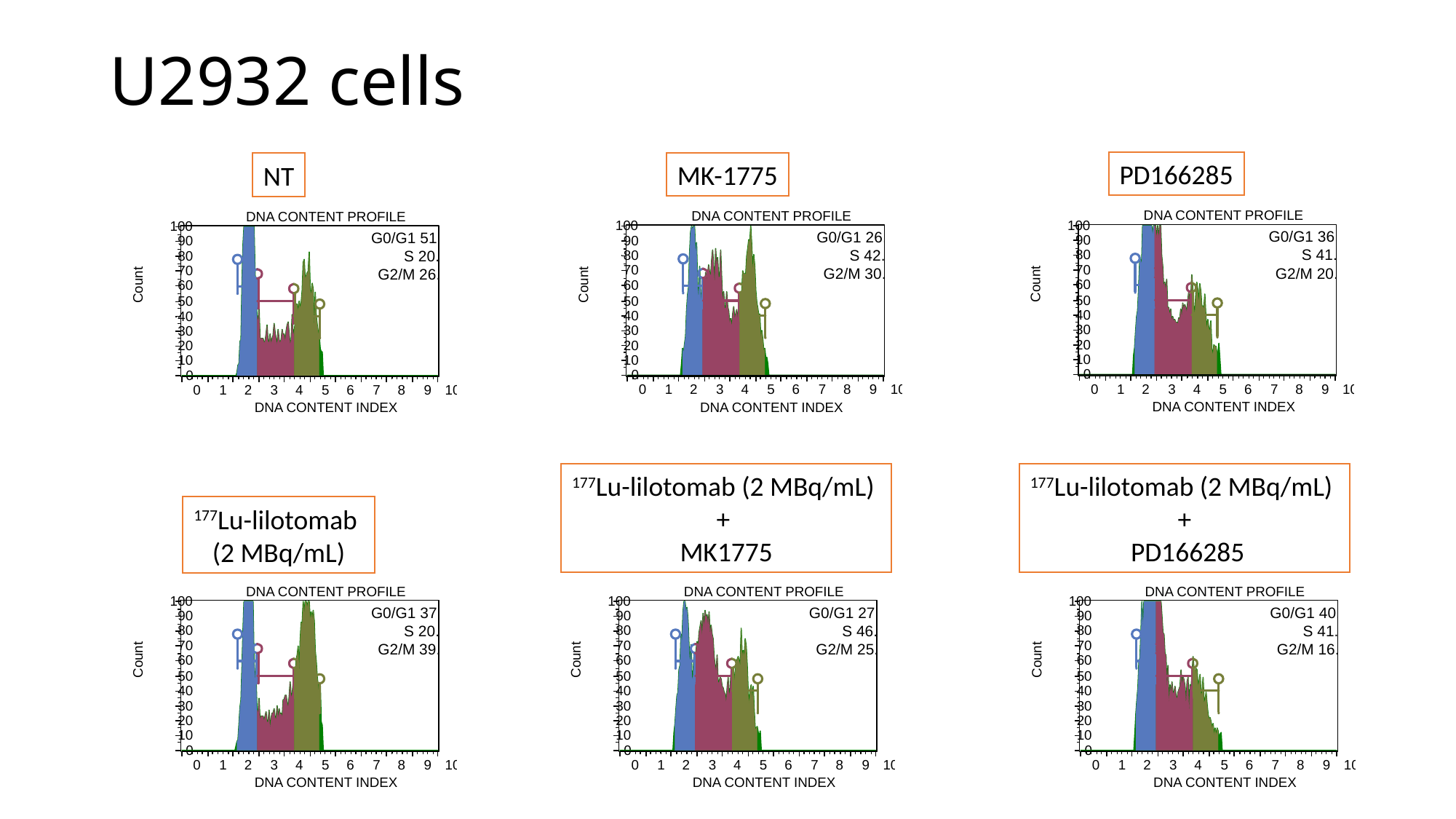

# U2932 cells
PD166285
MK-1775
NT
177Lu-lilotomab (2 MBq/mL)
+
MK1775
177Lu-lilotomab (2 MBq/mL)
+
 PD166285
177Lu-lilotomab
(2 MBq/mL)
